# *Calosota* Curtis (Hymenoptera, Chalcidoidea, Eupelmidae) – review of the New World and European fauna including revision of species from the West Indies and Central and North America

**DOI:** 10.3897/zookeys.55.490

**Published:** 2010-09-10

**Authors:** Gibson Gary A.P.

**Affiliations:** Agriculture and Agri-Food Canada, Canadian National Collection of Insects, K. W. Neatby Bldg., 960 Carling Avenue, Ottawa, Ontario, Canada, K1A 0C6

**Keywords:** Agrilus coxalis, goldspotted oak borer, Chalcidoidea, parasitoid

## Abstract

Two of three species previously classified in Calosota Curtis (Hymenoptera: Eupelmidae) from the Neotropical region are transferred to Eupelminae. Calosota eneubulus (Walker) from Galapagos Islands is transferred to Eupelmus Dalman as Eupelmus (Eupelmus) eneubulus (Walker), **comb. n.**, and Calosota silvai (Brèthes) from Chile is transferred to Brasema Cameron as Brasema silvai **comb. n.** Calosota cecidobius (Kieffer) from Argentina is retained in Calosota, with reservation, as an unrecognized species. The species of Calosota from the New World excluding South America are revised. Eleven species are recognized, including the seven newly described species Calosota albipalpus **sp. n.** (Costa Rica, Mexico, USA, Venezuela), Calosota bicolorata **sp. n.** (USA), Calosota elongata **sp. n.** (USA), Calosota longivena **sp. n.** (USA), Calosota panamaensis **sp. n.** (Panama), Calosota setosa **sp. n.** (Bahamas, Dominican Republic, USA), and Calosota speculifrons **sp. n.** (Costa Rica, USA). The 11 regional species and the Palaearctic species Calosota vernalis Curtis are keyed and illustrated. Calosota vernalis is not known to occur in the New World but is included in the key and diagnosed because it has been intercepted in quarantine in Canada. Calosota pseudotsugae Burks is placed in synonymy under Calosota acron (Walker), **syn. n.**,and Calosota kentra Burks, Calosota montana Burks and Calosota septentrionalis Hedqvist are placed in synonymy under Calosota aestivalis Curtis **syn. n.** Calosota modesta Bolívar y Pieltain is removed from synonymy under Calosota viridis Masi, stat. rev., and Calosota viridis, Calosota matritensis Bolívar y Pieltain, and Calosota coerulea Nikol’skaya are placed in synonymy under Calosota metallica (Gahan), **syn. n.** Calosota grylli Erdös is confirmed as a separate species from Calosota metallica based on features of both sexes. It is suggested that Calosota ariasi Bolívar y Pieltain may be a synonym of Calosota aestivalis, Calosota bolivari Askew may be a synonym of Calosota agrili Nikol’skaya, Calosota dusmeti Bolívar y Pieltain may be a synonym of Calosota violascens Masi, and Calosota lixobia Erdös likely is not a junior synonym of Calosota obscura Ruschka, but formal nomenclatural changes are not proposed prior to a comprehensive Palaearctic revision. Previous interpretation of the mesoscutum in Calosota and Balcha Walker is also modified to include the presence of anteroadmedian lines in addition to notauli and parapsidal lines.

## Introduction

Noyes (2003) listed five species of Calosota Curtis (Hymenoptera: Eupelmidae: Calosotinae) from the Nearctic region and three from the Neotropical region. The three Neotropical species have never been treated in a modern study except for the names being included in regional catalogs (e.g. De Santis 1967, 1979), whereas the five Nearctic species were revised by Burks (1973). Recently, both sexes of a species of Calosota reared in Arizona, USA, from the goldspotted oak borer, Agrilus coxalis Waterhouse (Coleoptera: Buprestidae), were sent to me for identification. This species of metallic wood-boring beetle was described originally from Mexico and Guatemala and is not known as a pest there or in Arizona, but in 2004 it was discovered in southern California causing extensive mortality to several oak species (Coleman and Seybold 2008a, 2008b). The female parasitoids did not key reliably to any of the North American species of Calosota recognized by Burks (1973) and subsequent study verified that they represented an undescribed species. However, study also revealed that there were several other undescribed species of Calosota in North America, some of which are more widely distributed south of the USA, and that at least two of the three Neotropical species were incorrectly classified to genus and subfamily. I do not describe new species restricted to South America because the five specimens I have seen are too few to evaluate species limits reliably, but I do include specimens from the West Indies and Central America because they provide important information concerning species distributions and sex associations for some of the North American species. Study also revealed that three of the five species Burks (1973) recognized in North America occur in Europe under different names. The objective of this study is not a revision of European Calosota, but study to clarify species limits and nomenclature for North American species resulted in new observations concerning most of the 13 species of Calosota recognized in Europe. These observations are included to help clarify species concepts and stimulate future studies in the region. During the study I also realized that my previous (Gibson 1989) interpretation of mesoscutal structure relative to the notauli in Calosotinae was inaccurate and it is corrected here.

## Materials and methods

Collections containing unexamined type material (indicated by an asterisk) and those containing specimens that were examined for this study are listed below. Collection acronyms are used in the text and the names of individuals who assisted in the loan of material are given in parentheses.

AEICAmerican Entomological Institute, Gainesville, FL, USA (D. Wahl).

BMNHThe Natural History Museum, London, England (S. Ryder).

CASCCalifornia Academy of Sciences, Department of Entomology, San Francisco, CA, USA (R. Zuparko).

CDFACalifornia State Collection of Arthropods, California Department of Food & Agriculture, Sacramento, CA, USA (S. Gaimari, J. Kishmirian).

CNCCanadian National Collection of Insects, Arachnids and Nematodes, Agriculture and Agri-Food Canada, Ottawa, ON, Canada.

EMECEssig Museum of Entomology, University of California, Berkeley, CA, USA (R. Zuparko).

FSCAFlorida State Collection of Arthropods, Division of Plant Industry, Gainesville, FL, USA (J. Wiley).

HNHMHungarian Natural History Museum, Department of Zoology, Budapest, Hungary (S. Csősz).

LACMLos Angeles County Museum of Natural History, Insect Collection, Los Angeles, CA, USA (B. Brown).

MACN*Museo Argentino de Ciencias Naturales “Bernardino Rivadavia”, e Instituto Nacional de Investigación de las Ciencias Naturales, Buenos Aires, Argentina (J. José Martinez).

MCSN*Museo Civico di Storia Naturale “Giacomo Doria”, Genoa, Italy.

NHMW*Natural History Museum, Vienna, Austria.

MNCNMuseo Nacional de Ciencias Naturales, Madrid, Spain (M. París).

MVMA*National Museum of Victoria, Melbourne, Australia.

NMPCNational Museum, Natural History, Prague, Czech Republic (P. Janšta).

RLZCR. Zuparko private collection, Berkeley, CA, USA (R. Zuparko).

TAMUTexas A&M University, College Station, TX, USA (E. Riley).

UCDCThe Bohart Museum of Entomology, University of California, Davis, CA, USA (S. Heydon).

UCFOUniversity of Central Florida Collection of Arthropods, Department of Biology, Orlando, FL, USA (S. Fullerton).

UCRCUCR Entomological Teaching and Research Collection, University of California, Riverside, CA, USA (S. Triapitsyn).

USNMUnited States National Entomological Collection, U.S. National Museum of Natural History, Washington, DC, USA (M. Gates).

ZMAS*Zoological Museum, Academy of Science, St. Petersburg, Russia.

ZSMCThe Bavarian State Collection of Zoology, Munich, Germany (S. Schmidt).

Observations and descriptions were made with a Nikon SMZ-U stereomicroscope illuminated with a halogen light source. A piece of translucent Mylar tracing acetate was taped to the objective between the light source and specimen to reduce glare (see “viewing specimens” in Gibson 2000). Color images were obtained with a Leica DC500 digital camera attached to a Leica Z16 APO macroscope; specimens were illuminated by fiber light sources shone through a Styrofoam diffuser. The serial images obtained were combined with AutoMontage and these and the scanning electron microphotographs obtained with a Philips XL30 environmental scanning electron microscope were digitally retouched using Adobe Photoshop CS4 to enhance clarity. Specimens that were used for macrophotography or scanning electron photomicrography are labeled with a “CNC Photo 2009-no.” or “CNC SEM 2009-no.” label, respectively. The photo or SEM number is included along with the collection data of the specimen under the respective species, and is included between parentheses in the figure captions for specimens other than holotypes and allotypes.

Terms for fore wing regions and folds follow Gibson (2004). Other terms for structure follow Gibson (1989, 1997) except for interpretation of anterior lines on the mesoscutum. In Calosotinae, parapsidal lines (= parapsides sensu Vilhelmsen et al. 2010) are present as slender regions of effaced or minute sculpture anterolaterally on the mesoscutum at the level of the lateral limit of the pronotum (Fig. 12: pl; Gibson 1989, fig. 72). Vilhelmsen et al. (2010) stated that parapsides are absent from Chalcidoidea; however, I hypothesized that presence of anteriorly rather than posteriorly positioned parapsidal lines is a possible homoplastic synapomorphy for Calosotinae in Eupelmidae (Gibson 1989, character 14: 3, fig. 1). I also stated that paramedial parallel lines of similar effaced or minute sculpture that extend from the posterior margin of the pronotum were the notauli and hypothesized that parallel rather than convergent notauli is a synapomorphy for Calosota, Balcha Walker and Tanythorax Gibson (Gibson 1989, character 7: 4; figs 35, 72). However, I did not appreciate that in Balcha and Calosota lateral to these straight lines of effaced sculpture are often very shallow furrows or slender lines of a different color and/or sculpture that curve from either spiracle to converge and posteriorly closely parallel or sometimes merge with the paramedial parallel lines of sculpture. Gibson (2005, fig. 33) provided a good SEM image of the complete complement of the three sets of lines, whereas a slightly incorrect position of the notaulus was indicated in Gibson (2005, fig. 36: not) and Gibson (1989, figs 35, 72: not). The straight, paramedial lines of effaced sculpture were indicated as the notauli in the latter images, but closely approximated shallow furrows lateral to these are the true notauli. Here, I use the term “anteroadmedian lines” sensu Daly (1964) and Gibson (1985) (= anteroadmedian sigma sensu Vilhelmsen et al. 2010) for the inner, straight, paramedial lines of effaced sculpture (Fig. 12: aal) interior to both the notauli (Fig. 12: not) and parapsidal lines (Fig. 12: pl). If the anteriorly positioned parapsidal lines of Calosotinae are truly homologous with those of other Hymenoptera then they are external indication of the lines of attachment of the dorsoventral flight muscles in the pharate pupa (Daly 1964). The anteroadmedian lines are the external indication of the anterior lines of attachment of the dorsolongitudinal flight muscles in the pharate pupa (Daly 1964), and were stated as absent from Chalcidoidea by Vilhelmsen et al. (2010). The anteroadmedian lines are more obvious than the notauli in some species of Balcha and Calosota, whereas in at least some Calosota  only the notauli are evident. Individuals of Tanythorax lack anteroadmedian lines and the lines previously interpreted as the notauli are the true notauli in this genus.

Type data for species described originally outside of North America are given under synonymy for the respective species, but for previously described regional species are included in material examined. Although the sexes of Calosotinae do not differ morphologically as conspicuously as do male and female Eupelminae, an allotype male is designated for new species if males are recognized. Exact label data are given for the holotype and allotype, with a semicolon used to designate data on different labels. Data for other specimens are standardized as in Gibson (2009). For new species, a description of the holotype and allotype is given that includes exact measurements and ratios for the specimens. Measurements were made using a 10 mm ocular grid having 100 divisions. Relative measurements cited in the text were all taken at maximum magnification where 1 unit = 0.054 mm, except for comparison of wing vein lengths where magnification was adjusted so that length of the stigmal vein equaled 10 units. Variation observed for other specimens assigned to the species is noted following the description of the holotype and allotype. Previously described species include separate, more general descriptions of both sexes that are based on regional specimens only. Abbreviations used in the descriptions are: **cc** (costal cell), **fu** (funicular segment) number, **IOD** (minimum distance between inner orbits), **LOL** (minimum distance between anterior and posterior ocellus), **MPOD** (maximum diameter of posterior ocellus), **mv** (marginal vein), **OOL** (minimum distance between ocellus and inner orbit), **pmv** (postmarginal vein), **POL** (minimum distance between posterior ocelli), and **stv** (stigmal vein). Measurements of antennal articles include length and, in parentheses, width. Width of antennal articles does not include the length of projecting setae and therefore measurements are influenced by setal density and the extent to which setae are appressed to the article. Length of the mesosoma is medial length in dorsal view from the anterior margin of the pronotum to the posterior margin of the propodeal foramen, whereas length of the gaster is medial length in dorsal view from the anterior margin of the petiole to the apex of the ovipositor sheaths. Axillar width is the superficial dorsal width, that is, the horizontal dorsal surface measured between its outer and inner margins not including the lateral axillar carina. Width of the scutellum is the maximum width including the lateral scutellar carinae. Apparent length of the scutellum differs depending on whether or not the mesonotum is arched. When arched, the dorsellum (= metascutellum of some authors) overlies the apex of the (meso)scutellum so as to conceal a usually somewhat differently sculptured and colored transverse frenal area that is normally exposed if the mesonotum is not arched. Consequently, the scutellum superficially appears shorter if the mesonotum is arched and for this reason its length to width ratio is based only on specimens with a nonflexed mesonotum that have the frenal area visible. Width of the syntergum is maximum width measured at the level of the cerci (transcercal width). Unless specified otherwise, length of the syntergum is measured in dorsal view between its posterior margin and the level of the carina that delimits each cercus anteriorly. This method of measuring syntergal length is used because the posterior margin of the penultimate tergum extends beyond the level of the cerci in females of most species, but the tergum is moveable to some extent so that the amount it overlaps the base of the syntergum differs in different specimens. When the posterior margin of the penultimate tergum does not extend fully over the base of the syntergum a bare, less coarsely sculptured basal region of the syntergum is exposed. Different methods of drying, such as air or critical-point drying, can also result in the gaster being variably collapsed, shrunken or inflated and therefore measurements and ratios involving the gaster are often quite variable.

For ease of description the head is divided into several general regions, including the vertex (dorsal surface between level of anterior ocellus and posterior orbits of eyes), occiput (convex surface dorsally posterior to outer orbits of eyes), frontovertex (vertex and convex part of frontal surface below anterior ocellus), scrobal depression (more or less ∩-like concave region above level of toruli that includes a smooth and shiny scrobe above each torulus), interantennal region (convex, triangular region between scrobes), parascrobal region (convex region between inner orbit and scrobal depression), and lower face (frontal surface below toruli), which includes a clypeal region (medial region delimited by vertical sulci or carinae) and paraclypeal region (surface between clypeal region and malar sulcus).

The acropleuron usually has a complex pattern of sculpture that most often is differentiated into three regions: a more or less mesal, variably elongate, obliquely angled coriaceous-granular or “microsculptured” region and much larger anterior and posterior regions of different sculpture that intergrade around the microsculptured region. Description of the sculpture is simplified to general sculpture type anterior and posterior of the microsculptured region. An important structural feature not previously used for species recognition in Calosota is whether the lower mesepimeron sensu Gibson (1989) is visible in lateral view as a convex, subtriangular or lunate region between the acropleuron, anteroventral margin of the metapleuron, and basal margin of the metacoxa (e.g. Figs 52, 56: lmp; Gibson 1989, figs 33, 34) or whether the acropleuron extends completely to the metapleuron and base of the metacoxa. For simplicity, the former structure is called an “exposed” lower mesepimeron and the later structure a “reduced” lower mesepimeron. When reduced, only a remnant of the putative lower mesepimeron remains below the level of the convex acropleuron between the posteroventral margin of the acropleuron and the basal margin of the metacoxa (e.g. Figs 51, 54: lmp). The lower mesepimeron can be bare (Figs 55−57) or setose (Figs 52, 53) whether exposed or reduced, though when reduced it usually has only one or a few inconspicuous setae (e.g. Figs 51, 81) that often are best observed from a ventrolateral or posteroventral view.

## Systematics

### Calosota

Curtis

Calosota Curtis 1836: folio 596. Type species: Calosota vernalis Curtis, by original designation.
Calosoter Walker 1837: 358. Type species: Calosoter vernalis Walker (= Calosota aestivalis Curtis), by subsequent designation (Westwood 1839: 72). Designation of Pteromalus eneubulus Walker as type species by Ashmead (1904: 288) incorrect because species not originally included. Synonymy by Gahan and Fagan 1923: 26.
Metacalosoter Masi 1917: 167–168. Type species: Metacalosoter frequens Masi, by monotypy. Synonymy by Gibson 1989: 60.
Calosota (Paracalosota) Masi 1922: 142. Type species: Calosota (Paracalosota) viridis Masi, by monotypy. Synonymy by Bouček 1988: 544.
Calosota (Hylephila) Masi 1927: 330. Type species: Calosota (Hylephila) stenogastra Masi, by monotypy. Synonymy by Bouček 1988: 544. Homonym of Hylephila Bilberg 1820 and Hylephila Rondani 1877, discovered by Ghesquière (1946: 368).
Calosota (Hylephilisca) Ghesquière 1946: 368. Replacement name for Calosota (Hylephila)Masi (1922); synonymy by Bouček 1988: 544.
Calosota (Minaia) Pagliano and Scaramozzino 1990: 5, 128. Nomen nudum, proposed as replacement name for Calosota (Hylephila) Girault 1927, which does not exist.

#### Recognition.

Gibson (1989) provided a key to differentiate Calosota from the other seven described world genera of Calosotinae. Individuals are differentiated from other New World Calosotinae by the following combination of features: axillae widely separated and with straight rather than incurved inner margins (Figs 71–78); mesoscutum without distinctly V-like convergent notauli, but anterodorsally with one or two sets of subparallel lines consisting of notauli and/or anteroadmedian lines between parapsidal lines (e.g. Fig. 12); scutellum truncate anteriorly and carinate laterally posterior to axilla; prepectus extending to tegula when mesonotum not arched; mesoscutum coriaceous to reticulate (Figs 71–78), but not umbilicate.

Gibson (1989) gave a detailed generic description for Calosota. Characteristic features that are not treated in the following descriptions are: dorsellum reticulate to reticulate-strigose and variably expanded over apex of scutellum depending on whether mesonotum flexed or not; female propodeum with foramen incurved to or almost to anterior margin and with lateral longitudinal furrow or carina delineating median, bowtie-like, plical region (Figs 74, 79), but male propodeum much longer medially and therefore plical region more distinctly longitudinally strigose- or carinate-coriaceous (Figs 72, 77); metapleuron setose; mesotarsus ventrally with row of yellowish to reddish tarsal pegs on either side; metatibia with inconspicuous row of spinelike denticles along dorsal length.

#### Previously described Neotropical species.

Noyes (2003) listed Calosota eneubulus (Walker, 1838), Calosota cecidobius (Kieffer, 1910), and Calosota silvai (Brèthes, 1917) as three Neotropical species of Calosota. Calosota chrysideus (Ashmead, 1900) had previously been transferred to Eusandalum Ratzeburg by Gibson (1989: 56).

Calosota eneubulus was described originally in Pteromalus Swederus by Walker (1838: 475) from males collected in Galapagos Islands (Charles’s Island), and subsequently was transferred by him to Calosoter Walker (Walker 1846: 52). I examined two male syntypes in the BMNH (one labeled as lectotype by Z. Bouček in 1979) and determined that they are males of a species of Eupelmus (Eupelmus) Dalman; therefore, I hereby classify the species as Eupelmus (Eupelmus) eneubulus (Walker) comb. n.

Calosota silvai was described originally in Calosoter by Brèthes (1917: 26–27) from an unstated number of females reared from the eggs of Macromphalia dedecora Feisthamel (Lepidoptera: Lasiocampidae) in Chile, and was transferred to Calosota by De Santis (1979: 11, 169). Two female syntypes on one card remain in MACN, plus a microscope slide with legs, a fore wing, and an antenna (J. Martinez, personal communication). Based on photographic images provided to me by J. Martinez, including a dorsal and lateral habitus, propodeal structure, fore wing venation and setal pattern, antennal structure, and mesotibial peg and mesotarsal peg pattern, I hereby transfer Calosota silvai to Brasema Cameron as Brasema silvai (Brèthes) comb. n.

Calosota cecidobius was described originally in Calosoter by Kieffer and Jörgensen (1910: 424) from Argentina and was transferred to Calosota by De Santis (1967: 172). Kieffer at least believed he had both sexes because the structure of the antenna was stated to be alike in both sexes. If so, Calosota cecidobius is very likely a member of Calosotinae because based on the rest of the description there is no indication of strong sexual dimorphism, which characterizes Eupelminae, and no genus of Neanastatinae is suggested by the description. The axillae are described as being separated by about their own width, which could indicate a species of Calosota, though other genera of Calosotinae (see Gibson 1989) and some female Eupelminae also have the axillae noticeably separated (see figs in Gibson 1995). Further, the original description states that the mesotarsus has two rows of short, black, thick spines ventrally. The mesotarsal pegs in Calosota can be somewhat yellowish-brown similar to the color of the tarsus, to reddish distally, but they are not black in distinct contrast to a lighter colored tarsus. Black mesotarsal pegs that contrast distinctly with the tarsus is more indicative of most female Eupelminae. The anal segment of the abdomen is also described as having a transverse row of erect black bristles. Although some female Calosota have quite distinct, longer, dark setae along the curved posterior margin of the syntergum, a transverse row of bristle-like setae is most obvious for females of Brasema that have the syntergal margin slightly emarginate rather than posteriorly curved in dorsal view. Most ambiguous is the statement “Thorax vorn gewölbt, am Mesonotum eingedrückt, Parapsiden furchen fehlen” (Thorax convex in front, impressed/concave at the mesonotum, parapsidal furrows absent). It is possible that Kieffer was referring to the mesoscutum rather than the entire mesonotum being impressed. Females of Calosota often have the mesoscutum quite abruptly, convexly raised behind the pronotum and often low convex or flat to slightly depressed dorsally anterior to a flat or variably convex scutellum (e.g. Fig. 27). The mesoscutum of female Eupelminae is characteristically concave posteromedially behind a convex anteromedial lobe between convex lateral lobes, with the anteromedial lobe being separated from the lateral lobes by variably developed V-like furrows (see figs in Gibson 1995). If Kieffer was in fact describing the mesoscutal structure of a female eupelmine it is likely he would have interpreted these furrows as parapsidal furrows rather than stating that these are absent. Furthermore, even though he described the two small protibial apical spines that are characteristic of both sexes of Eupelmidae (Gibson 1989) he did not mention mesotibial apical pegs. Mesotibial apical pegs, when present, usually are more obvious than protibial apical spines. They are absent from most Calosota but are possessed by females of the most likely eupelmine genera to have been described from South America, such as Brasema. The overall description of Calosota cecidobius therefore supports it as a member of Calosotinae and mostly likely as a member of Calosota, though I am not completely satisfied with this conclusion because of some aspects of the description. Future rearing of Tetradiplosis sexdentatus Kieffer & Jorgensen (Diptera: Cecidomyiidae) galls, the stated host, in Argentina should help resolve the uncertainty because all the coxae were described as whitish in Calosota cecidobius, which is presently unknown for any Calosotinae and is quite unusual for Eupelminae.

#### Key to species of Calosota Curtis in West Indies and Central and North America

.

**Table:** 

1	Fore wing disc either with linea calva (slender, oblique bare band distinctly separated from venation and basal fold by setae, Fig. 58) and/or with broad speculum (bare region contiguous with basal fold and parastigma, Fig. 59)	2
–	Fore wing disc uniformly setose or with only a slender, often arcuate bare band along basal fold and extreme base of mediocubital fold beyond basal fold (Figs 60−62)	4
2(1)	Fore wing disc with broad speculum, though sometimes with a few scattered setae within bare area (Fig. 59)	Calosota metallica Gahan
–	Fore wing disc with linea calva (Fig. 58)	3
3(2)	Maxillary palpus dark brown; scutellum and mesoscutum similarly meshlike reticulate (Figs 12, 28, 74); mesopleuron with distinctly setose, exposed lower mesepimeron (Fig. 52)	Calosota acron (Walker)
–	Maxillary palpus white (Fig. 9); scutellum elongate reticulate-strigose in distinct contrast to meshlike reticulate-coriaceous mesoscutum (Figs 19, 76); mesopleuron with lower mesepimeron reduced, with only a linear horizontal strip below level of acropleuron bearing 1 or 2 setae between posteroventral margin of acropleuron and base of metacoxa (Fig. 81)	Calosota albipalpus sp. n. [part]
4(1)	Head with scrobal depression, excluding smooth and shiny scrobes, reticulate to transversely reticulate-alutaceous and frontovertex finely, meshlike coriaceous or at most extremely shallowly reticulate in small part (Figs 3, 69); mesopleuron with lower mesepimeron reduced, the acropleuron extending to metapleuron and base of metacoxa (Fig. 54) [species intercepted but not known to be established in North America]	Calosota vernalis Curtis
–	Head with scrobal depression almost or entirely smooth and shiny if frontovertex finely, meshlike coriaceous (Figs 9, 10, 66) or both frontovertex and scrobal depression (excluding scrobes) similarly reticulate (Figs 2, 4−6, 11, 70); mesopleuron often with bare or setose exposed lower mesepimeron (Figs 53, 55−57)	5
5(4)	Frontovertex finely, meshlike coriaceous and scrobal depression smooth and shiny or only obscurely coriaceous dorsally (Figs 9, 10, 66); male: flagellum often conspicuously elongate-filiform and setose, more than twice as long as width of head and with outstanding curved setae (Figs 47, 48), though sometimes as below (Fig. 50)	6
–	Frontovertex variably distinctly reticulate or at least most of face above level of interantennal region reticulate, the sculptured region usually V-like tapered toward interantennal region medially (Figs 2, 4−6, 11, 70); male: flagellum more or less clavate, less than twice as long as width of head and with very short setae (Figs 29, 44−46)	9
6(5)	Maxillary and labial palpi dark brown; mesopleuron with exposed, setose lower mesepimeron (Figs 9, 10); male: flagellum clavate with short setae similar to female (cf. Figs 20, 50)	7
–	Maxillary and labial palpi white (Fig. 53); mesopleuron with lower mesepimeron reduced, with only slender band below level of acropleuron above base of mesocoxa, though band posteriorly often with one or more projecting setae in region near intersection of meso- and metacoxae (Figs 51, 81); male: flagellum conspicuously elongate-filiform and with long curved setae (Figs 47, 48)	8
7(6)	Scutellum and mesoscutum bright green with variable reddish-coppery lusters under some angles of light (Fig. 21); mesoscutum strongly, coarsely reticulate, and scutellum with similar though smaller reticulations (Fig. 21); tegula yellow	Calosota panamaensis sp. n.
–	Scutellum and mesoscutum medially comparatively dark relative to at least partly brighter colored lateral lobes (Fig. 20); mesoscutum very shallowly reticulate with flat-bottomed reticulations medially and coriaceous laterally, and scutellum elongate strigose-reticulate (Figs 20, 78); tegula brown	Calosota speculifrons sp. n.
8(6)	Mesoscutum entirely, coarsely, and more or less uniformly reticulate (Fig. 77); legs beyond coxae mostly yellow (Figs 43, 47), the profemur rarely extensively brownish on posterior surface but at least anterior surface yellow; female and often male with base of scape distinctly (about 0.2 length) white (Fig. 43)	Calosota setosa sp. n.
–	Mesoscutum very shallowly reticulate to reticulate-coriaceous laterally, and with conspicuously larger, flat-bottomed reticulations medially compared to sculpture of lateral lobes (Figs 19, 76); legs with at least profemur and often metafemur extensively dark over both anterior and posterior surfaces (Figs 38, 48); both sexes with at most only extreme basal margin of scape white	Calosota albipalpus sp. n. [part]
9(5)	Female	10
–	Male	14
10(9)	Flagellum conspicuously elongate-slender (Fig. 36), combined length of flagellum and pedicel more than twice width of head, all funiculars except fu1 at least 2.5× as long as wide, and fu2 about 4× as long as wide	Calosota elongata sp. n.
–	Flagellum variable, but combined length of flagellum and pedicel distinctly less than twice width of head, at least apical funiculars less than 2× as long as wide, and fu2 less than 3× as long as wide	11
11(10)	Syntergum conspicuously elongate-slender, in lateral view at least 4× as long as high and about as long or longer than metatibia (Fig. 37), and in dorsal view at least 1.75× as long as medial length of penultimate tergum (Fig. 18)	Calosota longiventris Burks
–	Syntergum comparatively short, in lateral view at most about 2.5× as long as high and only about half length of metatibia (Figs 33–35), and in dorsal view only about as long as penultimate tergum (Figs 13, 15, 16)	12
12(11)	Middle leg entirely yellowish-orange beyond coxa, though femur usually somewhat more distinctly orange than lighter tibia (Fig. 34); scutellum almost or entirely dark in distinct contrast to metallic colored mesoscutum (Fig. 16); fore wing infuscate behind marginal and stigmal veins within about anterior half of wing (Figs 16, 34)	Calosota bicolorata sp. n.
–	Middle leg with femur variably conspicuously and extensively dark, but at least darker apically so leg with obviously lighter knee (Figs 33, 35); scutellum and mesoscutum similar in color (Figs 13−15); fore wing sometimes hyaline or virtually so (Figs 35, 60, 62)	13
13(12)	Fore wing with marginal vein usually less than 3× but at most 3.2× length of stigmal vein (Fig. 60) and often at least very slightly infuscate behind marginal vein; face with dark or coppery M-like band between inner orbits below anterior ocellus, the band partly differentiated by distinct green or bluish spot below anterior ocellus (Fig. 2); mesoscutum usually with variably distinct dark to coppery or greenish paramedial longitudinal bands (Fig. 13), though sometimes bands not or only very obscurely differentiated (Fig. 14)	Calosota aestivalis Curtis
–	Fore wing with marginal vein at least 3.5× length of stigmal vein and always hyaline (Fig. 62); face usually without obviously differentiated M-like band between inner orbits (Fig. 4); mesoscutum more or less uniformly dark (Fig. 15) to variably metallic but not longitudinally banded	Calosota longivena sp. n.
14(9)	Flagellum plus pedicel about 1.75× as long as head width, and funiculars, including fu1, all at least about 2× as long as wide	Calosota elongata sp. n.
–	Flagellum plus pedicel at most only about 1.5× as long as head width, and funiculars distinctly less than 2× as long as wide	15
15(14)	Legs dark except knees and often tarsi yellowish (Fig. 46); fu1 usually quite distinctly longer that wide	Calosota aestivalis Curtis
–	Legs sometimes entirely yellowish-orange beyond coxae, but at least middle leg and metatibia yellowish-orange (Figs 45, 49); fu1 slightly widened distally and at most about as long as maximum width	16
16(15)	Legs with profemur extensively, protibia partly, and metafemur variably distinctly infuscate to dark (Fig. 45); basal fold setose and mediocubital fold completely setose or only narrowly bare apical to basal fold; mesoscutum and scutellar-axillar complex uniformly dark or sometimes mesoscutum and extreme anterior margin of scutellum dark bluish, but scutellum otherwise dark and meshlike to longitudinally reticulate (Fig. 31)	Calosota longiventris Burks
–	Legs entirely yellowish-orange beyond coxae (Fig. 49); basal fold and mediocubital fold both basal and apical to its juncture with basal fold extensively bare (Fig. 61); mesoscutum and extreme anterior margin of scutellum bright green to reddish-coppery, the scutellum otherwise dark but more distinctly longitudinally strigose-reticulate than above (Figs 30, 75)	Calosota bicolorata sp. n.

#### Calosota acron

(Walker)

**Figures 1−6.**
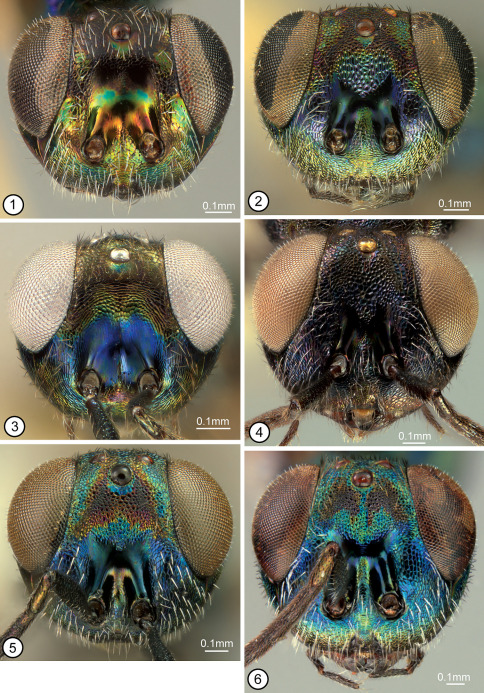
Head, frontal view. **1** Calosota acron ♀ (15) **2** Calosota aestivalis ♂ (17) **3** Calosota vernalis ♀ (43) **4** Calosota longivena ♀ (31) **5** Calosota elongata ♀ (holotype) **6** Calosota longiventris ♀ (22). Numbers in parentheses = CNC Photo 2009-no.

Figs 1 12 28 42 52 58 67 74

Eupelmus Acron Walker 1848: 129, 219. Type data: England. Lectotype ♀ (BMNH, type no. 5.1619; not examined), designated by Graham 1969: 91.
Trigonoderus contractus Walker 1872: 85–86. Type data: England. Holotype ♀ (BMNH, type no. 5.3328; not examined), by monotypy. Synonymy by Graham 1969: 91.
Calosota anguinalis Ruschka 1921: 251. Type data: Austria, Thüringen. Syntypes, ♀ (NHMW; not examined). Synonymy by Bouček 1968: 236.
Calosota acron ; Bouček 1968: 236.
Calosota pseudotsugae Burks 1973: 27–29. Holotype ♀ (USNM, type no. 72481; examined), by original designation. **syn. n.**

##### Description.

###### FEMALE

(Figs 28, 42). Length about 2.8–4.7 mm. Color. Head (Fig. 1) with variably large green spot below anterior ocellus and variably extensively and conspicuously green along inner orbit except parascrobal region always with dark band at about dorsal limit of interantennal region, usually partly green within scrobal depression and on interantennal region under different angles of light, and at least obscurely green (sometimes greenish with coppery luster under some angles of light) on lower face, but otherwise dark from level of posterior margin of eyes to about dorsal level of interantennal region, and parascrobal region usually dark along scrobal depression; posterior surface of head dark to sometimes greenish-blue under different angles of light except more distinctly bluish-purple in variably complete ∩-shaped band along outer orbit and occiput. Maxillary and labial palpi dark. Antenna dark except scape variably extensively yellow basally (usually with about basal quarter to third yellow), and dark part of scape and pedicel sometimes with green luster under some angles of light. Tegula yellow. Mesoscutum (Fig. 28) variably extensively greenish-blue to bluish-purple laterally, but parapsidal line, anteroadmedian line or region between anteroadmedian line and notaulus, and dorsomedially anterior to base of scutellum coppery or greenish with coppery luster; scutellar-axillar complex mostly similar in color to mesoscutum medially except frenal area bluish-purple. Acropleuron (Fig. 52) variably bluish-green except microsculptured region or diffuse region extending obliquely from microsculptured region toward tegula coppery. Legs (Fig. 42) with femur and tibia of front leg variably extensively dark, but trochanter and trochantellus at least distinctly lighter in color and knee, apex of tibia, and tarsus yellow; middle leg often entirely yellow beyond coxa but sometimes with similar color pattern as front leg except femur and tibia much lighter brownish-yellow; hind leg usually yellow beyond coxa though sometimes up to about basal two-thirds of femur brownish or dark with very slight metallic luster. Fore wing hyaline; setae uniformly brown. Gaster (Fig. 42) mostly brown dorsally but syntergum and gaster laterally more bluish-green, similar to mesosoma laterally.

*Structure/setation.* Head in dorsal view about 1.9–2× as wide as long, with IOD about 0.4–0.47× head width, OOL slightly more than half, LOL subequal to, and POL almost twice MPOD; in frontal view about 1.1–1.2× as wide as high, with ventral margin to about middle of torulus at level of lower orbits; malar space about 0.55–0.7× height of eye. Head (Figs 1, 67) with frontovertex finely meshlike coriaceous, the sculpture at least obscurely extended ventromedially within scrobal depression between smooth and shiny scrobes; parascrobal region finely coriaceous dorsally to somewhat more vertically coriaceous-alutaceous ventrally; clypeal region microcoriaceous, but interantennal region and paraclypeal region coriaceous-reticulate except smoother narrowly along lower inner orbit. Head with white setae except for bare scrobal depression. Antenna (Fig. 28) with scape about 3.6–4.2× as long as wide; pedicel about 2–2.3× as long as wide; flagellum clavate with length of flagellum + pedicle about 1.4× head width; combined length of fu1 + fu2 slightly greater than (larger specimens) to slightly less than (smaller specimens) length of pedicel; fu1 obviously longer than wide except in small specimens, but less than 1.5× as long as wide; subsequent funiculars oblong basally to only slightly longer than wide or subquadrate apically with fu2 about 1.5–2× and fu8 at most about 1.2× as long as wide; clava often slightly collapsed (compressed), but about as long as apical three funiculars. Mesoscutum (Figs 12, 28, 74) meshlike-reticulate with somewhat larger flat-bottomed reticulations medially, and with comparatively inconspicuous white setae; usually with quite deep and distinct notauli on inclined anterior surface and with quite distinct parapsidal lines, but with only obscure anteroadmedian lines on anterior inclined surface indicated by longitudinal region of slightly different color or sculpture. Axillae (Figs 28, 74) large, almost equilateral-triangular in smaller specimens, and separated by only about 1–1.5× own width. Scutellum low convex, about 1.3–1.4× as long as wide; similarly reticulate as mesoscutum laterally (Fig. 74); with inconspicuous white setae. Mesopleuron (Fig. 52) with exposed, setose lower mesepimeron; acropleuron variably extensively reticulate anteriorly, becoming more coriaceous to obliquely coriaceous-alutaceous anterior to oblique microsculptured region and very finely, longitudinally to slightly obliquely alutaceous-aciculate posteriorly. Fore wing (Fig. 58) with cc: mv: stv: pmv about 30–35: 21–25: 10: 17–20; basal cell entirely setose; cubital area usually quite extensively setose behind mediocubital fold and/or apically, and closed by setae along posterior margin over about apical half; disc basally with oblique bare band separated from basal fold, parastigma and base of marginal vein, and with short region of mediocubital fold bare just beyond basal fold. Metacoxa setose along dorsal and ventral margins and outer surface usually extensively though less densely setose basally. Propodeum with callus setose to posterior margin; bare anteriorly between spiracle and foramen. Gaster (Figs 28, 42) about 1.6–2× as long as mesosoma; more or less uniformly covered with inconspicuous white, hairlike setae; penultimate tergum with posterior margin extending to or slightly beyond level of cerci; syntergum about 1–1.8× as long as transcercal width, uniformly convex, and about 0.8–0.9× as long as penultimate tergum.

###### MALE

(based on single regional specimen). Similar to female except as follows. Antenna with scape more robust, only about 3× as long as wide; fu1 + fu2 about 1.4× length of pedicel, fu1 about 2× as long as wide, fu8 about 1.2× as long as wide, and clava only slightly longer than combined length of apical two funiculars. Fore wing with cc: mv: stv: pmv = 34: 27: 10: 18.

##### Biology.

Burks (1973) stated that the type material of Calosota pseudotsugae was reared from downed Pseudotsuga menziesii (coast Douglas-fir) along with seven other insect species and suggested that it probably was reared from Pseudohylesinus nebulosus (LeConte) (Coleoptera: Curculionidae: Scolytinae). Deyrup (1975) later determined that it was a hyperparasitoid of Pseudohylesinus nebulosus through Spathius sequoiae Ashmead (Hymenoptera: Braconidae), one of the species originally reared with the type material. Two other females were also reared in North America through Spathius sequoiae from the alder bark beetle, Alniphagus aspericollis (LeConte) (Coleoptera: Curculionidae: Scolytinae). In addition to Pseudotsuga menziesii, other regional tree associates are Alnus ruba (red alder), Tsuga heterophylla (western hemlock), and Thuja sp.

Noyes (2003) does not list any associates for Calosota acron, but Graham (1969) reported rearing females in Oxford, England, from “old trellis-work infested with Anobium striatum (Olivier)” (Coleoptera: Anobiidae) (2♀ BMNH) and based on label data another observed female from Oxford (BMNH) was reared as a hyperparasitoid of Tetropium sp. (Coleoptera: Cerambycidae) through Xylonomus [= Xorides Latreille] (Hymenoptera: Ichneumonidae). Fusu (2009) also reared Calosota acron from dry branches of Carpinus (hornbeam) and Fagus (beech) together with Xestobium sp., and Xestobium plumbeum (Illiger) and Anobium fulvicorne Sturm (Coleoptera: Anobiidae), respectively. The host records indicate Calosota acron is at least a facultative hyperparasitoid.

##### Regional material examined

**Map 1.**
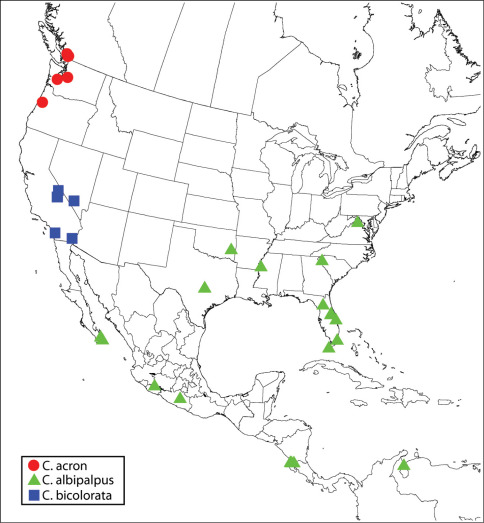
Regional distribution of Calosota acron, Calosota albipalpus and Calosota bicolorata.

(Map 1). **CANADA**. ***British Columbia***: BC Hydro Site, 49°09.3374'N; 122°52.2023'W, 22.VII.08, from Thuja, N. Furness (1♀ CNC). Stanley Park, 3c Pipeline Drive, 49°18.46314'N; 123°08.52413'W, 26.VI.08, 6.VII.08, from Tsuga heterophylla, N. Furness (7♀ CNC, CNC Photo 2009-13, 2009-14, 2009-15, CNC SEM 2009-33, 2009-34). Surrey, Dogwood RV Site, 49°12.5989'N; 122°48.3367'W, 6.VI.08, from Thuja plicata, N. Furness (1♀ CNC). **USA**. ***Oregon***: Benton Co., Mary’s Peak (nr Corvallis), 15.VIII.84, M.E. Schauff & E.E. Grissell, roadside meadow (1♀ USNM). ***Washington***: King Co., Cedar Falls, 12.III.75, M.A. Deyrup, from Spathius sequoiae from Alniphagus aspericollis in Alnus ruba (2♀ CNC, CNC Photo 2009-50). Thurston Co., Maytown, Jct. Rt. 121 & US 5, 24.II.72, M.A. Deyrup, reared from Pseudotsugae menziesii in insectary, 6, 7, 8, 9, 12 (CNC Photo 2009-48) Apr. (♀ holotype, ♂ allotype and 4♀ paratypes of Calosota pseudotsugae).

##### Distribution.

Noyes (2003) listed Calosota acron from several countries in Western Europe; I saw females from Croatia (CNC), England (BMNH), France (CNC Photo 2009-33, USNM) and Sweden (BMNH). The species has only been rarely collected in North America in the lower Fraser Valley in British Columbia and Washington and Oregon (Map 1). This restricted distribution and its host biology strongly indicates Calosota acron is not a naturally occurring Holarctic species but likely was introduced accidentally in wood products relatively recently through the port of Vancouver (Canada) or perhaps Seattle (USA).

##### Recognition.

Although I did not examine the lectotype of Calosota acron, my concept of this name and new synonymy of Calosota pseudotsugae is based on the keys of Graham (1969) and Askew and Nieves-Aldrey (2006), and comparison of North American specimens with authoritatively identified specimens of Calosota acron from Europe in the BMNH. Some females seen from Europe are up to about 7.5 mm, their much larger size suggesting the possibility of different, larger hosts. The larger females have all the legs entirely yellow beyond the coxae, can have up to about the basal half of the scape yellow, typically have a more distinct coppery region on the mesoscutum extending posteriorly from each parapsidal line (Fig. 12), and the syntergum is somewhat more elongate-slender, up to about twice as long as the transcercal width, as indicated by Graham (1969). The larger females also have much more obvious anteroadmedian lines (Fig. 12), which appears to be at least partly correlated with specimen size in Calosota. However, I hereby synonymize the name Calosota pseudotsugae under Calosota acron syn. n. because North American and European specimens do not differ substantially in morphology. Askew and Nieves-Aldrey (2006) suggested that Calosota ariasi Bolívar y Pieltain (1929) might be a synonym of Calosota acron, but this European name more likely is a junior synonym of Calosota aestivalis if not a valid species (see under Calosota aestivalis).

Calosota acron is recognized primarily by the presence of an oblique fore wing linea calva (Fig. 58). Calosota albipalpus sometimes also has a variably developed oblique bare band, though typically there is then also a narrow bare region along the basal fold. Regardless, Calosota acron is readily differentiated from Calosota albipalpus by several features, including dark palpi and an exposed and at least sparsely setose lower mesepimeron (Fig. 52). Askew and Nieves-Aldrey (2006, fig. 14) noted that the outer surface of the metacoxa is mostly setose, but this is variable and smaller specimens typically have the outer surface more extensively bare mediolongitudinally (Fig. 52).

#### Calosota aestivalis

Curtis

Figs 2 13, 14 33 46 55 60 72

Calosota aestivalis Curtis 1836: folio 596. Type data: England: Southgate. Lectotype ♀ (MVMA; not examined), designated by Graham 1969: 90–91.
Calosoter vernalis Walker 1837: 359. Type data: England: near London; Ireland: Holywood. Syntypes, ♀ and ♂ (BMNH, type no. 5.1621; not examined). Synonymy by Graham 1969: 90–91. Homonym of Calosota vernalisCurtis (1836).
Calosota fumipennis Bolívar y Pieltain 1923: 65–67. Type data: Spain: Villaviciosa de Odón. Holotype ♀ (MNCN; examined), by original designation. Synonymy by Askew and Nieves-Aldrey 2006: 89.
Calosota septentrionalis Hedqvist 1956: 96–97. Type data: Sweden: Fundort: Dalekarlien, Älvdalen 1955. Holotype ♀ (K. Hedqvist personal collection; examined). **syn. n.**
Calosota kentra Burks 1973: 30–31. Holotype ♀ (USNM, type no. 72482; examined), by original designation. **syn. n.**
Calosota montana Burks 1973: 31. Holotype ♀ (USNM, type no. 72483; examined), by original designation. **syn. n.**

##### Description.

###### FEMALE

(Figs 13, 33). Length about 3–6.5 mm. Color. Head (Fig. 2) variably dark greenish-blue to purple, including spot below anterior ocellus, but with more or less complete coppery or dark transverse band on vertex between inner orbits (Fig. 14) (sometimes reduced to large spot behind ocellar triangle and adjacent to each upper inner orbit) and with M-like coppery or dark region on upper face (region very rarely narrowly divided medially below anterior ocellus), with lateral arm of region usually extending dorsally to or toward posterior ocellus (sometimes filling ocellar triangle and rarely contiguous with transverse band on vertex) and ventrally abutting inner orbit. Maxillary and labial palpi dark. Antenna dark brown except scape sometimes with metallic luster similar to lower face. Tegula dark. Mesoscutum (Figs 13, 14) variably dark greenish-blue to purple similar to head except almost always with at least obscurely differentiated dark, coppery or greenish paramedial longitudinal bands (Fig. 13) (anteriorly each band sometimes subdivided into band occupying region between parapsidal line and notaulus, and narrower band extending posteriorly from anteroadmedian line, and posteriorly paramedial bands sometimes broadly contiguous); scutellar-axillar complex or at least scutellum mostly same color as mesoscutal paramedial bands, the axillae more commonly and often margins of scutellum similar in color to remaining mesoscutum. Acropleuron (Fig. 55) dark with slight coppery luster to variably greenish-blue or purple similar to most of head and mesoscutum. Legs (Fig. 33) mostly brown with knees, apices of tibiae, and at least basal tarsomeres of meso- and metatarsi yellowish, but usually tarsi more extensively yellowish and meso- and metatibiae sometimes also mostly or entirely yellowish-brown to yellowish. Fore wing hyaline or disc variably extensively and conspicuously infuscate; setae uniformly brown. Gaster (Fig. 33) usually dark brown with slight reddish-coppery luster under some angles of light or laterally partly blue to purple.

*Structure/setation.* Head in dorsal view about 1.8−2.1× as wide as long, with IOD about 0.33−0.45× head width, LOL at least slightly greater than and sometimes up to about 2× OOL and slightly less than to slightly greater than MPOD, and POL about 1.2−1.7× MPOD; in frontal view about 1.2−1.3× as wide as high, with dorsal margin of torulus about at level of lower orbits; malar space about 0.5−0.7× eye height. Head (Fig. 2) with frontovertex and upper parascrobal region meshlike reticulate to about level of dorsal limit of interantennal region, medially the reticulations tapered ventrally between dorsal limits of smooth and shiny scrobes; lower parascrobal region and interantennal region much shallower meshlike reticulate to coriaceous-reticulate; clypeal region microcoriaceous to granular and paraclypeal region obliquely reticulate-alutaceous. Head sometimes with whitish setae except for bare scrobal depression but more commonly with brownish setae on frontovertex and more conspicuous white setae on parascrobal region, interantennal region and lower face. Antenna (Fig. 33) with scape about 4.6−5.3× as long as wide; pedicel about 2.5−3.3× as long as wide; flagellum variably distinctly clavate (funiculars all about same width and clava usually only slightly wider than funicle except if compressed) with length of flagellum + pedicel about 1.3−1.75× head width; combined length of fu1 + fu2 about 1−1.7× as long as pedicel; fu1 about 1.3−2.3× as long as wide; subsequent funiculars all longer than wide with fu2 about 2−3.1× and fu8 at least slightly and sometimes up to about 1.5× as long as wide; clava often collapsed, but about as long as apical 2.5−3.5 funiculars. Mesoscutum (Figs 13, 14, 72) more or less uniformly meshlike reticulate, with inconspicuous white setae; notaulus extending from spiracle as curved furrow on inclined anterior surface, its posterior limit contiguous dorsally with posterior limit of anteroadmedian line; parapsidal line usually quite a distinct region of microsculpture posterior to spiracle. Axillae elongate-triangular, separated by about 3−4× own width (Fig. 72). Scutellum flat to low convex and at least slightly (up to about 1.2x) longer than wide; similarly reticulate as mesoscutum (Fig. 72); with inconspicuous white setae. Mesopleuron (Fig. 55) with exposed, bare lower mesepimeron; acropleuron variably deeply and distinctly meshlike reticulate anterior to oblique microsculptured region and longitudinally coriaceous-alutaceous posteriorly. Fore wing (Fig. 60) with cc: mv: stv: pmv about 40−60: 23−31: 10: 14−16, and perpendicular distance between apex of stigmal vein and anterior margin of wing usually about 0.7−0.8x, only very rarely up to about 9.5x, length of stigmal vein; basal cell entirely setose; cubital area bare except sometimes anteriorly near mediocubital fold, and up to about apical half closed by setae along posterior margin; disc setose except usually for short region of mediocubital fold just beyond basal fold or with variably broad and distinct, often lunate bare region along basal fold, the bare region sometimes continuous with cubital area. Metacoxa setose along dorsal, ventral and usually basal margins, but sometimes up to about basal third of outer surface setose. Propodeum with callus setose to posterior margin; bare anteriorly between spiracle and foramen. Gaster (Figs 13, 33) about 1.8−2.2× as long as mesosoma; sparsely setose dorsally and more densely setose laterally with white to brownish hairlike setae; penultimate tergum with posterior margin extending to or slightly beyond level of syntergum; syntergum about 1.5−2.6× as long as transcercal width, variably distinctly compressed depending on length, and about 0.7−1.3× as long as penultimate tergum.

###### MALE

(Fig. 46). Similar to female except as follows. Length about 2.5−3.8 mm. Color. Legs (Fig. 46) always extensively dark with knees and tarsi often yellowish but tibiae usually only slightly lighter apically; all males examined with M-like region on upper face and with lateral arms extending ventrally to inner orbits (Fig. 2); mesonotum more commonly (particularly smaller individuals) without distinct paramedial bands; fore wing disc often, but at most only very slightly infuscate.

*Structure/setation.* Antenna with scape more robust, only about 3.4−3.9× as long as wide; flagellum of smaller individuals sometimes more distinctly clavate, the funicle evenly widened toward clava; fu1 variably distinctly widened distally and sometimes only about as long as wide, but usually quite obviously (up to about 1.5x) longer than wide; fu2 about 1−1.9× as long as wide or length of fu1; combined length of fu1 + fu2 about 0.65× (smallest specimens) to about 1.25× length of pedicel; and fu8 quadrate to slightly longer than wide. Fore wing venation similar to female with cc: mv: stv: pmv about 38−52: 22−31: 10: 13−14, and perpendicular distance between apex of stigmal vein and anterior margin of wing about 0.8−0.9× length of stigmal vein; basal cell and disc completely setose or sometimes disc with arcuate bare band along basal fold, but bare region only rarely continuous with cubital area. Propodeal callus sometimes setose to posterior margin, and then rarely with one or more setae anteriorly between spiracle and foramen, but more often setose only to level about equal with posterior margin of spiracle, with 1 or 2 setae often behind spiracle but bare anteriorly between spiracle and foramen.

##### Biology.

Burks (1973) stated that the type specimens of Calosota montana were reared from an unidentified gall on Pinus contorta (lodgepole pine). The female from British Columbia labeled as reared from Dioryctia sp. (Lepidoptera: Pyralidae) is a Forest Insect Survey specimen that very likely represents an incorrect host association; however, the California host record of Anthaxia sp. (Coleoptera: Buprestidae) reared from Quercus (oak) is more likely correct even though coniferous trees such as Pinus (pine) and Pseudotsuga (Douglas-fir) are more commonly indicated as tree associates in North America. Noyes (2003) listed several host species in Europe in Anobiidae, Cerambycidae, Cleridae, and Curculionidae including Scolytinae (Coleoptera) emerging from Betulaceae, Fabaceae, Fagaceae, Pinaceae and Tamaricaceae. The cited records of Trichodes leucopsideus (Olivier) (Cleridae) and associated Megachile sp. (Hymenoptera: Apidae) are incorrect because of previous historical misidentification of Calosota vernalis as Calosota aestivalis (see below and under Calosota vernalis). All documented host records indicate Calosota aestivalis is a primary parasitoid (Noyes 2003).

##### Regional material examined

**Map 2.**
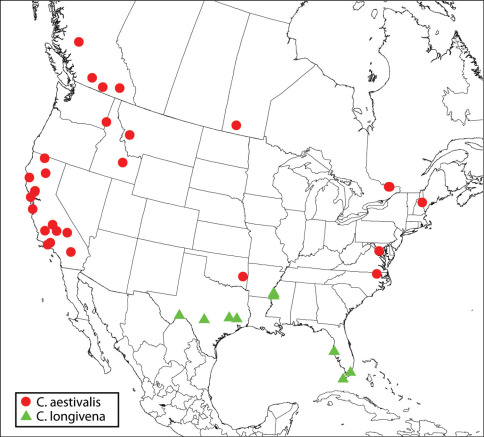
Regional distribution of Calosota aestivalis and Calosota longivena.

(Map 2). **CANADA**. ***British Columbia***: Anahim Lake to Redstone, 1000–1500 m., 17.VII.88, S&J Peck (1♂ CNC). Beaverdell, 7.VII.61, FIS (Forest Insect Survey), 60-8056-01, ex. Dioryctria sp. (1♀ CNC). Brookmere, 21.VII.33, K. Graham (1♀ CNC, CNC Photo 2009-47). Kaslo, 24.VI.03, R.P. Currie (1♂ USNM). O.K. Centre, 2.V.30, 17461 Lot1, A.C. Thrupp (1♀ CNC, CNC Photo 2009-44). ***Manitoba***: Onah, 10.VII.84, R.M. White, Tamarack (1♀ CNC). ***Quebec***: Gatineau Pk, Ridge Rd, 27.V–10.VI.6[?] (1♀ CNC). Pontiac, Eardley, 5.VI.91, S. Laplante (1♀ CNC, CNC Photo 2009-16). **USA**. ***California***: Argus Mts, V.1891 (1♂ paratype of Calosota longiventris, USNM). Santa Cruz Mts, antenna on slide (1♂ USNM). Kern Co., Glennville, em. III, IV.67, J.W. Tilden (1♀ CNC Photo 2009-20, 1♂ EMEC). Marin Co., Lagunitas, 14.VII.28, E.H. Nast (1♀ EMEC). Mendocino Co., NCCRP, 3 mi. E Branscomb, 1400’, 21–23.V.82, C. Besette (1♀ EMEC, CNC Photo 2009-19). Montery Co., Santa Lucia Mts, Junipero Serra Pk, on peak ca. 5800’, 27, 28 (CNC Photo 2009-17).VIII, 4.IX.56, 4.IV, 16.V.57, H.B. Leech, ex. dead branches Pinus coulteri (1♀, 4♂ CASC). Napa Co., 2 mi. NNE Angwin, N side Howell Mt., 1300’, 17, 20.V.76, R.R. Leech, ex. log Pseudotsuga menziessii (2♂ CASC); Butts Cyn Rd, 0.5 mi. S Snell Valley Rd, 11, 15 (CNC Photo 2009-18), 17, 23, 29.IV.78, R.B. Leech, ex. dead branches Pinus sabiniana (1♀, 4♂ CASC). San Bernardino Co., Burns Piñon Ridge Reserve, 1260 m., 34°08'57N; 116°27'11W, 21–23.V.05, K. Will et al. (2♀ RLZC), 23.V.05, R.L. Zuparko, Chilopsis linearis arcuata (2♀ RLZC). San Luis Obispo Co., 6 mi. SE Poso, R16E, T315, sects. 4–5, 1800’, 2.IV–4.V.89 (1♂ CNC), 1500’, 23.IV–4.V.89 (1♀, CNC), W.E. Wahl. Shasta Co., 2 mi. W Shingletown, 2750’, 15-20.VII. 85, R. Miller, damp open meadow with Rush (Juncus) and wild flower along stream in pine forest (1♂ FSCA). Siskiyou Co., Yreka, 4820a’ Hopk. U.S., reared, Quercus, probably ex. Anthaxia (1♀ USNM). Tulare Co., Tulare, 20.III.75, emerged 10.IV.75 (1♂ CDFA). Ventura Co., 25.IV.05, 2771 Hopk. U.S., bred, Pinus (1♀ USNM). ***Idaho***: Craters of the Moon Nat. Mon., summer ‘64, reared from Pinus flexilis (1♀ USNM). Latah Co., Moscow, Paradise Ridge, 3000’, 9.V.31, P. Rice (1♂ USNM). ***Montana***: Granite Co., Rock Creek, 10 (allotype), 11 (holotype).II.69, from unidentified gall on Pinus contorta, by J. G. Bringuel (♀ holotype, ♂ allotype of Calosota montana). ***New Hampshire***: Carroll Co., Albany, 2.VII.58, W.J. Morse (♀ holotype of Calosota kentra). ***North Carolina***: Northhampton Co., 7 km. S Jackson, 23.IX–15.XI.87, bald cypress swamp, BRC Hym. team (1♂ CNC). ***Oklahoma***: Latimer Co., Red Oak, IX.90, K. Stephan (1♀ CNC). ***Virginia***: Fairfax Co., near Annandale, 1–17.IV.90, D.R. Smith (1♂ CNC).

##### Distribution.

Noyes (2003) listed several countries in the Palaearctic region; I saw specimens from Corsica (CNC SEM 2009-48, ZSMC), Cyprus (NMPC), England (BMNH), France (BMNH, CNC), Jordan (CNC), Morocco (CNC), Slovakia (CNC), Spain (CNC) and Sweden (CNC). Its extensive, apparently transcontinental distribution (Map 2) and morphological variability in North America (see further below) suggest that it has been present in the region for a long time as a naturally occurring Holarctic species. Its presence in North America for a long time may also be supported by head sculpture pattern, which is shared with four other similar species in North America (Calosota bicolorata, Calosota elongata, Calosota longivena and Calosota longiventris), but not with any other species in at least Western Europe. This suggests that Calosota aestivalis may be more closely related to the four North American species than it is to any species from Western Europe. This hypothesis needs be tested through phylogenetic or molecular analyses.

##### Remarks.

Burks (1973: 31) inadvertently published the binomen Cecidostiba montana as the name of his new species preceding its description. However, this certainly was an unintentional printing error based on the title of the paper, proper generic placement of the species in the abstract and key, and other typographical errors in the type locality and type sections of the preceding description of Calosota kentra.

##### Recognition.

The concept of the names Calosota aestivalis and Calosota vernalis were incorrectly reversed in the literature beginning with Walker (1837) until corrected by Graham (1969). Although I did not examine the lectotype of Calosota aestivalis, my concept of this name is based on the keys of Graham (1969) and Askew and Nieves-Aldrey (2006) plus authoritatively identified specimens in the collections listed above. I examined the holotype female of Calosota septentrionalis in 1984 and at that time considered that it probably was a synonym of Calosota aestivalis, noting that the vertex was reticulate in both. Hedqvist (1956) compared his new species to Calosota aestivalis (as Calosota vernalis) and differentiated the latter species from Calosota septentrionalis and Calosota fumipennis based on the fore wing being either yellowish or more or less infuscate, respectively. Graham (1969) noted that European females of Calosota aestivalis often have the fore wings partly infuscate, which is the form that Bolívar y Pieltain (1923) described as Calosota fumipennis. Hedqvist (1956) differentiated Calosota septentrionalis from Calosota fumipennis on color differences as well as equally variable differences in the ratios of the marginal, postmarginal and stigmal veins, but stated specifically that Calosota septentrionalis has paramedial longitudinal greenish-bronze bands on the mesoscutum. Based on this color pattern, head sculpture pattern, and other features Hedqvist (1956) used in his key to differentiate species, I hereby synonymize Calosota septentrionalis under Calosota aestivalis syn. n., as was done previously for Calosota fumipennis by Askew and Nieves-Aldrey (2006).

Askew and Nieves-Aldrey (2006) suggested that another European name, Calosota ariasi Bolívar y Pieltain (1929), whose unique female holotype had been lost from its mount in MNCN, might also be a synonym of Calosota acron. They refrained from formalizing the synonymy because of some significant discrepancies between the original description of Calosota ariasi and specimens of Calosota acron. Based on its original description, Calosota ariasi more likely is a synonym of Calosota aestivalis than Calosota acron. The description of Calosota ariasi states that the head had a transverse coppery band behind the ocelli as well as a coppery band extending from each posterior ocellus to surround the anterior ocellus ventrally and widen on the upper face to about its mid height. This color pattern describes very accurately the color pattern of Calosota aestivalis (Fig. 2) but not Calosota acron (Fig. 1), which has the frontovertex mostly dark. The described bluish-black femora and brownish-black tibiae except for the knees and apex of the tibiae and very slightly infuscate fore wings also more strongly suggest Calosota aestivalis (Fig. 33) than Calosota acron. Askew and Nieves-Aldrey (2006) keyed Calosota ariasi along with Calosota vernalis, primarily because the original description states that the distance between the eyes was the same as the width of an eye. Description of head sculpture for Calosota ariasi (weakly shagreened dorsally and face strongly shagreened, almost scale-like) might also be interpreted as descriptive of the head sculpture of Calosota vernalis (see under this species), but the acropleuron is described as being very shagreened over its basal two-fifths, especially dorsally. This is characteristic of Calosota aestivalis (Fig. 55) but not Calosota vernalis (Fig. 54), which suggests description of the head sculpture actually referred to a somewhat coarser reticulate sculpture below than above the anterior ocellus that is also typical of Calosota aestivalis (Fig. 2). As noted by Askew and Nieves-Aldrey (2006), described shape and width of its axillae, which Bolívar y Pieltain (1929) used as key features to differentiate Calosota ariasi and Calosota aestivalis (as Calosota vernalis + Calosota fumipennis) from other Spanish Calosota, also contradicts the possibility of Calosota ariasi being synonymous with Calosota vernalis. The features used to distinguish Calosota ariasi from Calosota aestivalis by Bolívar y Pieltain (1929) (anellus only about one-third longer than wide versus twice as long as wide and syntergum twice as long versus only about as long as wide) certainly are within the range of variation noted for North American specimens I identify as Calosota aestivalis. It seems likely that Calosota ariasi is a synonym of Calosota aestivalis, but I hesitate to formalize the synonymy prior to a more comprehensive revision of Spanish and European Calosota.

When Burks (1973: 31) described Calosota montana he stated that it “greatly resembles the European species vernalis Curtis”, but it is apparent he was still misinterpreting the name Calosota vernalis in the sense of Calosota aestivalis based on other features listed as shared between “Calosota vernalis” and Calosota montana. The presence or absence of longitudinal banding on the mesoscutum (cf. Figs 13, 14), shading on the fore wing, and a bare region on the fore wing disc adjacent to the basal fold were all used by Burks (1973) to differentiate Calosota montana (western USA: Montana) from Calosota kentra (eastern USA: New Hampshire). Based on observation of more material, females with infuscate wings normally have the disc more or less uniformly setose to the basal cell whereas females with hyaline wings typically have a more definite bare band adjacent to the basal fold, but all the features used by Burks (1973) to differentiate Calosota montana and Calosota kentra appear to be present in different combinations, including structure of the stigma and uncus. Burks (1973, fig. 2) partly characterized the holotype of Calosota montana as having an enlarged stigma with a long, slender uncus. The holotypes of Calosota montana and Calosota kentra are both similar to European females of Calosota aestivalis in having the stigmal vein comparatively long and at an acute angle to the postmarginal vein such that the perpendicular distance between its apex and the anterior margin of the wing is only about 0.8× its length (Fig. 60). Both holotypes also have an M-like region differentiated on the upper face (cf. Fig. 2) as well as at least an obscurely differentiated transverse region on the vertex (Fig. 14), and the marginal vein is only about 2.4× as long as the stigmal vein (cf. Fig. 60). I consider the morphological differences between the type material of Calosota kentra and Calosota montana to represent intraspecific variation in one quite variable and widely distributed species in North America and therefore synonymize both Calosota kentra and Calosota montana under Calosota aestivalis syn. n. However, I also describe a new species, Calosota longivena, based on females that are morphologically very similar to some females I include in Calosota aestivalis except for relative length of the marginal vein and, usually, a somewhat differently structured stigmal vein. Furthermore, the single female from Oklahoma (Map 2) that I include in Calosota aestivalis is intermediate in some features between those I identify as Calosota aestivalis and Calosota longivena. In addition to having distinctly infuscate fore wings and an obvious bare band adjacent to the basal cell, the Oklahoma female is unusual in having a comparatively short and straight stigmal vein (perpendicular length from its apex to anterior margin of wing almost equal to its length) and an unusually long costal cell and marginal vein as compared to other females I identify as Calosota aestivalis. The Oklahoma female has the marginal vein about 3.2× and the costal cell about 6× as long as the stigmal vein compared to up to about 2.8× and up to about 4.8x, respectively, for other Calosota aestivalis females. Females of Calosota longivena have a shorter and more obtusely angled stigmal vein such that the marginal vein is at least about 3.6× the length of the stigmal vein and stigmal vein length is subequal to the distance from its apex to the anterior margin of the wing. Although the paramedial bands on the mesoscutum of the Oklahoma female are also somewhat obscurely differentiated it has quite a distinct M-like coppery region on the upper face. The combination of features of the Oklahoma female suggests the possibility of introgression or a morphological cline in what I identify as Calosota aestivalis and Calosota longivena. The southeastern Oklahoma origin of the aberrant female, between the known distribution of Calosota longivena (Florida to southwestern Texas) and what appears to be a more northern transcontinental and further eastern (California) distribution for Calosota aestivalis (Map 2), might also support a hypothesis of introgression or morphological cline. However, if females I describe as Calosota longivena represent only a highly modified southern form of Calosota aestivalis in North America then Calosota aestivalis is far more variable in North America than in Europe. Collection of males with females in the known range of Calosota longivena could help clarify morphological limits and species status, as could molecular analysis of specimens from throughout North America and Europe. Males I identify as Calosota aestivalis are most similar to those of Calosota longiventris (see further under latter species and Calosota bicolorata).

#### Calosota albipalpus sp. n.

urn:lsid:zoobank.org:act:6FCB905B-A05F-4FAA-9223-46D41AE87749

Figs 9 19 38 48 66 76, 81 

##### Etymology.

From the Latin words albus, ‘white’, and palpus, in reference to color of the maxillary and labial palpi, one of the differentiating features of this species.

##### Type material.

HOLOTYPE♀ (CNC type no. 23924). COSTA RICA: Guanacaste Prov., Guanacaste Nat. Pk, 27.IV–11.V.1985, D. Janzen & I. Gauld; pk. hdqts., H-1-O, young scrubby woodland, clearing; Sector Santa Rosa, 10°51'N; 85°37'W, 250–300m; Holotype Calosota albipalpus Gibson. ALLOTYPE♂ (CNC). COSTA RICA: Guanacaste Prov., Guanacaste Nat. Pk, 23.III–13.IV.1986, D. Janzen & I. Gauld; SE-7-O, clearing Bosque San Emilio, deciduous forest; Sector Santa Rosa, 10°51'N; 85°37'W, 250–300m; CNC Photo 2009-10; Allotype Calosota albipalpus Gibson.

Additional paratypes. **COSTA RICA**. Same data as holotype except as follows: SE-5-O, clearing Bosque San Emilio, deciduous forest (1♀ CNC); 23.III–13.IV.86 (1♀ CNC), H-1-O (1♂ CNC), H-4-C, shade (1♂ CNC); 17–27.IV.85, H-4-C, shade (1♀ CNC); 23.III–13.IV.86, SE-7-O, clearing Bosque San Emilio, deciduous forest (1♀ CNC); 4–24.V.86 (1♀ CNC); 24.VIII–14.IX.85 (1♂ CNC). Guan.[acaste], S.[anta] Rosa Park, 8.II.78, D.H. Janzen (1♂ AEIC). **MEXICO**. Baja California, Las Batracas [? Barracas], 1–11.V.89, P. DeBach (1♀ CNC); Sur. Los Barriles, 5–6.V.79, M. Wasbauer (1♀ CDFA). Guerrero, 6 mi. E Xochilapa, 18.VII.84, J.B. Woolley (2♀ CNC). Michoacan, Nueva Italia, 9.VII.85, J. Woolley (1♀ CNC), Woolley & Zolnerowich (1♀ CNC). **USA**. ***Florida***: Alachua Co., Gainesville, Am. Ent. Inst., 2–10.IV.86, M. Sanborne (1♂ CNC, CNC SEM 2009-43). Brevard Co., Malabar, Malabar Rd, Malabar Scrub Sanct., Fire Unit 16, xeric oak scrub, 29.VI–7.VII.01, 22.VII–3.VIII.01 (CNC Photo 2009-8, CNC SEM 2009-35), P.J. Russell, Z. Prusak & S.M. Fullerton (2♀ UCFO). Dade Co., S Miami, Deering Estate Pk For., SW 167 St & 72 Ave, 21.II–1.VI.86, S&J Peck, young hammock, MT-FIT (1♀ CNC, CNC SEM 2009-42). Monroe Co., Big Pine Key, Watsons Hammock, 28.VIII.86, S. Peck (1♀ CNC, CNC Photo 2009-9). Orange Co., Orlando, LLP-Sand Pine Turkey Oak S, 21.VI.96, S.M. Fullerton (1♀ UCFO, CNC SEM 2009-36). Seminole Co., Oviedo, 17.IV.94, rural yard, S.M. Fullerton (1♂ UCFO). ***Oklahoma***: Latimer Co., V.89, K. Stephan (1♀ FSCA). ***Mississippi***: Washington Co., Delta Exp. Forest, Stoneville, 33°28'N; 90°54'W, 16.VIII–5.IX.97, N.M. Schiff (1♂ UCDC). ***South Carolina***: Anderson Co., Pendleton, 225 m., 14.VIII–9.IX.87, BRC Hym. team (1♂ CNC, CNC SEM 2009-41). ***Texas***: Travis Co., vic. Cypress Creek, 30°25'58N; 97°52'01W, 13–14.VII.94, on Ulnus crassifolia, M. Quinn, E. Riley & R. Wharton (1♀ TAMU); Long Hollow Cr., 30°27'43N; 97°52'19W, 8.V.93 (1♀ TAMU), 23.IV.83 (2♂ TAMU), Alexander, Quinn, Riley, Wharton, et al. ***Virginia***: Clarke Co., Blandy Exp. Farm, 2 mi. S Boyce, 25.VII–7.VIII.90, D.R. Smith (1♀ CNC, CNC Photo 2009-7). **VENEZUELA**. Maracaibo, 24.IV.81, H.K. Townes (1♀ AEIC); sea level, 22–24.IV.81, H. Townes (1♀ CNC).

Excluded from type series. **DOMINICA**. W.I., St. John Parrish, Cabrits Natl. Pk, West Cabrits Hill, 15°35'06N; 61°28'37W, 600’, 22–30.V.00, L. Benavides, E. Chavez, J. Dye & E. Kretsch, malaise 2000/015-016 (2♂ USNM).

##### Description.

###### FEMALE

(Figs 19, 38). HOLOTYPE: length 2.3 mm. Color. Head (Fig. 9) with frontovertex and scrobal depression dark brown with slight bluish-green lusters under some angles of light, but smooth part of parascrobal region yellowish to reddish-coppery and interantennal region and lower face distinctly greenish-blue to purple under different angles of light. Maxillary and labial palpi white (Fig. 9). Antenna dark brown except scape and pedicel dorsally with slight bluish-green lusters under some angles of light. Tegula dark. Mesoscutum (Fig. 19) largely dark similar to frontovertex, but under some angles of light posterior region of larger meshlike-reticulations with coppery luster and anteriorly with greenish-blue to bluish-purple lusters; scutellar-axillar complex similar in color to mesoscutum posteriorly. Acropleuron variably reddish-violaceous to bluish-green under different angles of light. Legs (Fig. 38) with femur and tibia of front leg extensively dark brown, but trochanter, trochantellus, knee, about apical quarter of tibia, and tarsus yellowish-white; middle leg yellowish-white beyond coxa except femur with short subapical dark brown band (reduced on dorsal surface) and tibia with short subbasal dark brown band opposite femoral band when appressed to femur; hind leg yellowish beyond coxa except about basal half of femur brown and dorsal margin of tibia with short subbasal brownish region. Fore wing hyaline; setae uniformly brown. Gaster mostly with reddish-coppery lusters dorsomedially, but more yellowish-green paramedially to greenish-blue laterally (Fig. 38), except penultimate tergum reddish-coppery basally to more yellowish-green apically and syntergum and Gt1 basally bluish-purple.

*Structure/setation.* Head in dorsal view about 1.66× as wide as long, with IOD about 0.42× head width; IOD: MPOD: OOL: POL: LOL = 35: 7: 5: 14: 9; in frontal view about 1.3× as wide as high, with about middle of torulus at level of lower orbits; malar space about 0.55× eye height. Head (Figs 9, 66) with frontovertex and upper parascrobal region finely coriaceous; scrobal depression, lower parascrobal region, and paraclypeal region along inner orbit almost or completely smooth and shiny; clypeal region and interantennal region meshlike coriaceous, but lower parascrobal region near scrobal depression and most of paraclypeal region obliquely coriaceous-alutaceous. Head with brownish setae on frontovertex but more whitish setae on interantennal region and lower face. Antenna (Fig. 38) with flagellum clavate; length of flagellum + pedicel about 1.4× head width; scape: pedicel: fu1–fu8: clava = 36(9): 14(6): 4(4), 10(5), 10(5), 10(5), 10(6), 9(7), 9(7), 9(8): 35(12). Mesoscutum with inclined anterior surface and lateral lobes meshlike coriaceous, but dorsal surface anterior to scutellar-axillar complex very shallowly meshlike reticulate, the reticulations large and flat-bottomed, and with sparse, brownish setae; notauli, anteroadmedian lines and parapsidal lines all obscure. Axillae elongate-slender, separated by about 4× own width (Fig. 76). Scutellum low convex, only slightly longer than wide; elongate strigose-reticulate (Fig. 76); with inconspicuous dark setae. Mesopleuron with reduced lower mesepimeron, but narrow vertical surface under convex acropleuron above base of mesocoxa with 2 setae projecting between bases of meso- and metacoxae (Fig. 81); acropleuron without distinct miscrosculptured region, very finely, obliquely coriaceous-alutaceous anteriorly and much more elongate, longitudinally alutaceous-aciculate posteriorly. Fore wing with cc: mv: stv: pmv = 54: 53: 10: 12; basal cell entirely setose; cubital area bare, including along posterior margin to about level of basal fold; disc with oblique bare band behind base of marginal vein (distinct only for left wing) separated from marginal vein and from posteriorly widened bare region along parastigma and basal fold, the latter bare area continuous with cubital area because of short, bare region of mediocubital fold beyond basal fold. Metacoxa setose along dorsal, ventral and basal margins, and outer surface with a few scattered setae within about basal third. Propodeum with callus setose to posterior margin; bare anteriorly between spiracle and foramen. Gaster (Fig. 38) about 1.7× as long as mesosoma; with sparse, inconspicuous white setae dorsally and somewhat more conspicuous, slightly lanceolate white setae laterally; penultimate tergum with posterior margin extending to level of cerci; syntergum only very slightly longer than transcercal width, evenly convex, and about as long as penultimate tergum.

###### MALE

(Fig. 48). ALLOTYPE: length 1.4 mm. Similar to holotype except as follows. Color. Mostly brownish (Fig. 48) with much less distinct metallic lusters, only lower face with distinct greenish luster under some angles of light and propodeum somewhat bluish-violaceous; legs with about medial third of posterior surface of mesofemur brownish, all but about apical quarter of metafemur brown, and dorsal surface of metatibia more extensively brownish.

*Structure/setation.* Head in dorsal view about 1.75× as wide as long, with IOD about 0.48× head width; IOD: MPOD: OOL: POL: LOL = 33: 8: 4: 15: 8; in frontal view about 1.25× as wide as high, with ventral margin of torulus about at level of lower orbits; malar space about 0.56× eye height. Antenna with flagellum conspicuously setose and elongate-filiform, the curved setae somewhat longer than width of respective flagellomere; length of flagellum + pedicel about 2.1× width of head; scape: pedicel: fu1–fu11 = 21(8): 10(7): 2(4), 14(5), 16(5), 16(6), 15(6), 15(6), 13(6), 13(6): 13(6): 10(5): 12(5) (including very small apical subsegment). Fore wing with cc: mv: stv: pmv = 50: 55: 10: 12; oblique bare band behind marginal vein distinct for both wings. Scutellum elongate-reticulate rather than distinctly strigose. Metacoxa with outer surface bare mediolongitudinally except for single row of setae along basal margin.

##### Variation.

Females range in length from about 1.5–3 mm and males from about 1.3–2 mm. The metallic lusters of the body vary in intensity, smaller individuals usually being more brownish with less distinct metallic lusters and larger specimens often having the mesoscutum broadly brown with coppery luster medially and the lateral lobes with variably extensive, though quite dark and comparatively inconspicuous green, bluish and/or purple lusters under some angles of light. Females sometimes also have the extreme base of the scape white, the fore wing setae more whitish, though not obviously lighter behind the submarginal than the marginal vein, and the hind legs less extensively brown or rarely completely yellow beyond the metacoxa. Setal pattern of the fore wing disc varies in both sexes. Some specimens have an essentially uniformly setose disc, including a uniformly setose mediocubital fold that extends somewhat basal of the level of the basal fold, whereas others have quite a distinct, posteriorly open bare band along the parastigma and basal fold and/or quite a distinct linea calva (cf. Fig. 58). When present, the two bare bands are usually separated by setae, but in some specimens the linea calva is continuous with the basal bare band. Some females have the mesoscutum more uniformly, though extremely shallowly meshlike reticulate (Fig. 76) to almost coriaceous, the cells defined by only very slightly raised ridges similar to most males. Some females also have the scutellum more distinctly elongate-reticulate than strigose and smaller females tend to have slightly shorter funiculars such that the apical ones are quadrate rather than slightly elongate.

I exclude from the type series of Calosota albipalpus two males from Dominica that have the mesoscutum mostly smooth and shiny mediolongitudinally anterior to the base of the scutellum (only extremely obscure meshlike coriaceous sculpture under some angles of light), the fore wings more extensively setose (disc without evident bare regions and about apical half of cubital area closed by setae posteriorly) than for other males, and the tegula yellow similar to males of Calosota setosa. These males likely belong to a different species than Calosota albipalpus, but females from Dominica are required to adequately assess variation and species limits.

##### Biology.

Unknown.

##### Distribution.

New World, extending between about 35°N in North America and 10°N in South America (Map 1). Calosota albipalpus is one of four species comprising a species complex that is united both by head sculpture and distribution. All specimens of Calosota seen from the New World south of the USA have a finely coriaceous frontovertex and a mostly smooth and shiny scrobal depression, suggesting that they comprise a species group that possibly evolved in the Neotropical region, of which some of the species (Calosota albipalpus, Calosota speculifrons and Calosota setosa) subsequently expanded their ranges into southern USA.

##### Remarks.

The last (eleventh) flagellomere of the filiform flagellum of males of Calosota albipalpus and Calosota setosa has a tiny, setose, narrower apical region that is variably distinctly differentiated by a suture. When this “subsegment” is distinct it appears like a tiny fourteenth antennomere, but likely is homologous with the ventroapical micropilose sensory region of the apical clavomere of females of the species.

##### Recognition.

Calosota albipalpus, Calosota panamaensis, Calosota setosa and Calosota speculifrons share a finely coriaceous frontovertex and mostly smooth and shiny scrobal depression. Calosota albipalpus more closely resembles Calosota setosa because individuals have white palpi (Figs 9, 10), lack an exposed, convex lower mesepimeron (Fig. 81), and males have a conspicuously setose and elongate-filiform flagellum (Figs 47, 48). Individuals of Calosota albipalpus and Calosota setosa differ primarily in mesoscutal sculpture. The mesoscutum is quite distinctly and more uniformly reticulate in Calosota setosa (Fig. 77) than in Calosota albipalpus, which has the mesoscutum more or less evenly coriaceous-reticulate or coriaceous laterally and only very shallow reticulate with large flat-bottomed cells medially (Fig. 76). Additionally, both sexes of Calosota albipalpus always have a dark scape and the front leg and often the metafemur much more extensively dark (Figs 38, 48) than individuals of Calosota setosa (Figs 43, 47), which also have yellowish tegulae and the mesoscutal lateral lobes often bright green or the mesoscutum more or less uniformly reddish-coppery.

Calosota acron is the only other regional species that has a distinct linea calva (see further under this species).

#### Calosota bicolorata sp. n.

urn:lsid:zoobank.org:act:7DA1C5D0-750E-4395-AB3F-4555681F2E8F

Figs 11 16 30, 34 49 61 75 

##### Etymology.

From the Latin words bi, ‘two’, and color, ‘hue’, in reference to the conspicuously different color of the mesoscutum and scutellum of females.

##### Type material.

HOLOTYPE ♀ (CNC type no. 23925). [USA] CAL. Riverside Co., Menifee Vly (hills on W end), 33°9'N; 117°13'W, 1800’ el., 1–15.VI.1980, John D. Pinto; CNC SEM 2009-38; CNC Photo 2009-11; Holotype Calosota bicolorata Gibson. ALLOTYPE♂ (UCDC). [USA] Batchelder Spr., Cal., Inyo Co., VII.11.1969, R.O. Schuster Colr.; CNC Photo 2009-12; Allotype Calosota bicolorata Gibson.

Additional paratypes. **USA**. ***California***: Imperial Co., Glamis, Hwy 78, 28.III.80, J. Woolley (1♀ CNC). Lone Pine, 2 mi. E, 19.V.70, E.E. Grissell (1♀ UCDC); Movie Rd, 1390 m., on Eriogonum fasic., 36°35'55N; 118°6'57W, 30.V.04, D. Yanega, UCR Ent. Res. Mus. 97429 (1♀ UCRC). ***Nevada***: Nye Co., Mercury, N.T.S., B.Y.U. - A.E.C., Code CS-A-5, Coll. No. 36, 3.IX.59, Ref. No. 778 (1♂ USNM, CNC Photo 2009-46, CNC SEM 2009-45).

##### Description.

###### FEMALE

(Figs 16, 34). HOLOTYPE: length 4.2 mm. Color. Head (Fig. 11) bluish-green, including spot below anterior ocellus, or with limited purplish luster under some angles of light, but vertex with coppery region between posterior ocelli extending from ocelli to level of outer orbits (Fig. 16), and upper face with M-like coppery region below anterior ocellus, the lateral arm of region inconspicuously tapered to outer margin of ocellus dorsally and slightly separated from inner orbit ventrally (Fig. 11). Maxillary and labial palpi dark. Antenna dark brown except scape with slight bluish-purple luster under some angles of light. Tegula dark. Mesoscutum (Fig. 16) bluish-green similar to head except anteroadmedian and parapsidal lines dark with slight coppery luster; scutellar-axillar complex black but reticulations shiny with slight coppery luster under some angles of light. Acropleuron primarily reddish-brown except for slight greenish luster anteriorly. Legs (Fig. 34) with front leg extensively brown but trochantellus, knee, tibia apically and tarsus yellowish; middle leg almost uniformly yellowish-orange beyond coxa though femur somewhat more orange and tibia and tarsus more yellowish; hind leg with most of femur brown and tarsus and most of tibia yellowish, but about apical one-fifth of femur yellowish and tibia brownish dorsomedially. Fore wing hyaline except for brownish region behind marginal and stigmal veins anterior to mediocubital fold; setae uniformly brownish within hyaline regions. Gaster (Fig. 34) mostly brown except terga with slight bluish-purple lusters laterally.

*Structure/setation.* Head in dorsal view about 2.1× as wide as long, with IOD about 0.36× head width; IOD: MPOD: OOL: POL: LOL = 57: 13: 7: 18: 13; in frontal view about 1.25× as wide as high, with dorsal margin of torulus at level of lower orbits; malar space about 0.52× eye height. Head (Fig. 11) with frontovertex and parascrobal region very shallowly meshlike reticulate to about level of dorsal limit of interantennal region, medially the reticulations tapered ventrally between dorsal limits of smooth and shiny scrobes; lower parascrobal region coriaceous-alutaceous; interantennal region finely coriaceous-alutaceous dorsally to meshlike coriaceous ventrally; clypeal region microcoriaceous and paraclypeal region obliquely coriaceous to very shallowly reticulate-alutaceous toward inner orbit. Head with inconspicuous, mostly brownish setae on frontovertex and more conspicuous white setae on parascrobal region, interantennal region and lower face. Antenna (Fig. 34) with flagellum clavate; length of flagellum + pedicel about 1.4× head width; scape: pedicel: fu1–fu8: clava = 75(13): 28(10): 13(8), 17(9), 21(10), 20(10), 17(10), 16(10), 15(10), 15(10): 50(13). Mesoscutum (Figs 16, 75) with inclined anterior surface alutaceous-coriaceous, inclined lateral surface shallowly meshlike reticulate, and dorsal surface more deeply meshlike reticulate, with quite conspicuous and dense, very slightly lanceolate white setae; notauli obscure, but anteroadmedian lines and parapsidal lines more distinct as lines of differentiated sculpture and color. Axillae very small, separated by about 5× own maximum width (Fig. 75). Scutellum convex, quadrate; elongate reticulate-strigose with deep, much smaller reticulations than on mesoscutum (Fig. 75); with inconspicuous dark setae. Mesopleuron with exposed, bare lower mesepimeron; acropleuron meshlike coriaceous or coriaceous-reticulate anterior to oblique microsculptured region and very finely, slightly elongate meshlike coriaceous posteriorly. Fore wing with cc: mv: stv: pmv = 50: 22: 10: 13; basal cell entirely setose; cubital area bare but closed by setae along posterior margin over about apical quarter; disc setose except for slender, arcuate bare region along basal fold, the region separated from cubital area by setose mediocubital fold. Metacoxa quite densely and conspicuously setose dorsally and ventrolaterally, the ventrally setose region broadening basally to restrict longitudinal bare band on outer surface within dorsal half. Propodeum with callus densely and conspicuously setose to posterior margin; anteriorly with single white setae midway between spiracle and foramen. Gaster (Figs 16, 34) about 1.7× as long as mesosoma; with hairlike to very slightly lanceolate white setae, the basal terga more sparsely setose dorsally but apical terga and tergal laterally relatively densely and uniformly setose; penultimate tergum with posterior margin extending distinctly beyond level of cerci (partly an artifact of abnormally inflated gaster as a result of critical-point drying); syntergum about 1.7× as long as transcercal width, uniformly convex, and subequal in length to penultimate tergum (measurements approximate because of inflated gaster).

###### MALE

(Fig. 49). ALLOTYPE: length about 3.1 mm. Similar to holotype except as follows. Color. Head (Fig. 30) mostly reddish-coppery, though under some angles of light interocellar region indistinctly and scrobes more distinctly greenish, and interantennal region with bluish-purple spot between toruli; mesoscutum (Fig. 30) extensively reddish-coppery similar to head except convex, inclined lateral surface of lateral lobes more greenish under most angles of light and parapsidal lines dark; scutellar-axillar complex narrowly reddish-coppery anteriorly, but scutellum mostly dark except for reddish-coppery frenal area; acropleuron irregularly reddish-coppery with slight greenish lusters under some angles of light; legs (Fig. 49) entirely yellowish-orange beyond coxae; gaster dark brown.

*Structure/setation.* Head in dorsal view (Fig. 30) about 1.9× as wide as long, with IOD: MPOD: OOL: POL: LOL = 62: 16: 8: 18: 12; in frontal view about 1.15× as wide as high, with ventral margin of torulus about at level of lower orbits; malar space about 0.58× eye height. Antenna with flagellum robust, about same width throughout and with setae much shorter than width of flagellomere, length of flagellum + pedicel about 1.3× head width, and fu1 distinctly widening apically; scape: pedicel: fu1–fu8: clava = 60(20): 32(12): 9(10), 15(13), 17(13), 17(12), 16(12), 13(12), 14(12), 13(12): 42(12). Fore wing (Fig. 61) with cc: mv: stv: pmv = 43: 24: 10: 10; basal cell mostly bare with only a few scattered setae, the basal fold completely and mediocubital fold broadly bare apical to basal fold and almost completely bare basal to basal fold, hence slender arcuate bare band on disc continuous with basal cell and cubital area. Propodeum (left side) with 2 setae anteriorly between spiracle and foramen.

##### Variation.

Females range in length from about 2.5–3.7 mm and the two known males from 2.9–3.1 mm. All females are quite similar in color except the coppery band behind the ocelli (Fig. 16) varies from being almost completely absent to forming a transverse band behind the posterior ocelli between the inner orbits, the M-like band on the upper face usually does not extend to the anterior ocellus, the interantennal region usually is not differentiated in color ventrally, the mesoscutum sometimes has an obscure coppery medial band extending posteriorly, and the scutellar-axillar complex is often entirely dark. Smaller females are somewhat less conspicuously setose than the holotype. Although the head of the non-allotype male is extensively reddish-coppery, it is quite distinctly green within the interocellar region and under some angles of light lateral to the anterior ocellus as well as ventrally on the parascrobal region, and the mesoscutum dorsally is also somewhat more extensively greenish. The fore wing of the non-allotype male is somewhat more extensively setose than for the allotype, but the basal fold and mediocubital folds are extensively bare so that the bare region of the disc along the basal fold is broadly continuous with the cubital area (Fig. 61).

##### Biology.

Unknown.

##### Distribution.

Nearctic: southwestern USA (Map 1) and likely northern Mexico.

##### Recognition.

As indicated in the key, females are most similar to those of Calosota aestivalis. The key also uses leg color as the primary feature to differentiate males of Calosota bicolorata from those of Calosota aestivalis and Calosota longiventris, but fore wing setal pattern may be a better feature. Both known males of Calosota bicolorata have the fore wing much less setose basally than those of Calosota aestivalis and Calosota longiventris, the basal fold and the mediocubital fold basal and apical to the basal fold being extensively bare (Fig. 61). However, additional males are required to determine whether this setal pattern is truly diagnostic for males of the species.

#### Calosota elongata sp. n.

urn:lsid:zoobank.org:act:6F85C2BA-DEF3-4A12-9EAC-303F4423FB68

Figs 5 17 36 44 70, 71 

##### Etymology.

From the Latin word elongatus, ‘prolonged’, in reference both to the elongate-slender flagellum and syntergum of females.

##### Type material.

HOLOTYPE♀ (UCDC). USA AZ Pima Co., Santa Rita Mtns., Coronado Natl. For., S of Box Cyn Rd nr jct. with Forest Service Road 231; 31°47.9'N; 110°45.6'W, T12S, R15E, N1/2 of section 12, coll. 2.V.2.2009, em. 6–7.VII.2009, TW Coleman, A Cippilone; ex. Quercus emoryi, bark & phloem in trunk, associated with Agrilus coxalis (goldspotted oak borer); CNC Photo 2009-25; Holotype Calosota elongata Gibson. ALLOTYPE♂ (UCDC). [USA] AZ: Pima Co., Santa Rita Mountains, Coronado Nat. Forest, S. of Box Cyn Rd, near X with FS [Forest Service] Road 231, 31.79961°N; 110.75921°W, V.2.2009, coll. T.W. Coleman, A. Cippilone; ex. bark and phloem of main stem of Emory oak, Quercus emoryi associated with the goldspotted oak borer, Agrilus coxalis Waterhouse (Coleoptera: Buprestidae), T19S, R15E, N 1/2 of Sec. 12; CNC Photo 2009-49; Allotype Calosota elongata Gibson.

Additional paratypes. **USA**. ***Arizona***: Cochise Co., Chiricahua National Monument, nr horse trailer parking lot, 32.00816°N; 109.3736°W, 10.III.10, T.W. Coleman, ex. bark and phloem of main stem of Emory bark, Quercus emoryi, associated with goldspotted oak borer, Agrilus coxalis Waterhouse (Coleoptera: Buprestidae) (1♀ CNC; 1♀ UCRC; 2♀, 1♂ USNM). Pima Co., same data as holotype (5♀ UCDC, CNC Photo 2009-24, CNC SEM 2009-49); same data as holotype except em. 12–14.VII.09 (4♀, 1♂ CNC, CNC SEM 2009-50); same data as allotype (1♀ UCDC).

##### Description.

###### FEMALE

(Figs 17, 36). HOLOTYPE: length 6.5 mm. Color. Head (Fig. 5) primarily dark bluish-green, including spot below anterior ocellus, but with transverse coppery band on vertex between inner orbits and more or less M-like coppery region on upper face, the lateral arm of region extending dorsally contiguous with anterior ocellus to posterior ocellus and ventrally almost touching inner orbit; lower face broadly dark along oral margin, including clypeal region; posterior surface of head dark or greenish under some angles of light except more distinctly bluish-purple in ∩-shaped band along outer orbit and occiput. Maxillary and labial palpi dark. Antenna dark brown except scape with slight greenish luster under some angles of light. Tegula yellowish-brown. Mesoscutum (Fig. 17) with inclined, convex part of lateral lobe bluish-purple except margin above prepectus darker or somewhat greenish under some angles of light, and dorsally with slender greenish band medially over about anterior half, but otherwise dark dorsally with yellowish or reddish-coppery lusters under some angles of light, the dark region posteriorly about as wide as base of scutellum and widening anteriorly to include parapsidal lines; scutellar-axillar complex with axillae and frenal area bluish purple, but most of scutellum similar in color to mesoscutum medially. Acropleuron bluish-purple to more greenish or coppery under some angles of light, particularly microsculptured region. Legs (Fig. 36) extensively brown with knees, tibiae apically, and tarsi mostly yellowish, the mesotibia somewhat more extensively yellowish. Fore wing hyaline; setae uniformly brown. Gaster (Figs 17, 36) mostly dark brown but dorsally with slight coppery sheen under some angles of light and first gastral tergum distinctly bluish-purple laterally.

*Structure/setation.* Head in dorsal view about 2× as wide as long, with IOD about 0.4× head width; IOD: MPOD: OOL: POL: LOL = 62: 15: 10: 18: 10; in frontal view about 1.16× as wide as high, with dorsal margin of torulus distinctly below level of lower orbits; malar space about 0.65× eye height. Head (Figs 5, 70) with frontovertex and parascrobal region meshlike reticulate to about level of dorsal limit of interantennal region, medially the reticulations tapered ventrally between dorsal limits of smooth and shiny scrobes and laterally parascrobal region more transversely reticulate-rugulose; interantennal region finely meshlike coriaceous and clypeal region microcoriaceous; parascrobal region obliquely coriaceous-alutaceous below rugulose region and paraclypeal region meshlike to obliquely reticulate. Head with brownish setae on frontovertex and more conspicuous white setae on parascrobal region, interantennal region and lower face. Antenna (Fig. 36) with flagellum conspicuously elongate-slender; length of flagellum + pedicel almost 2.3× head width; scape: pedicel: fu1–fu8: clava = 84(17): 31(11): 21(9), 47(10), 45(10), 40(10), 33(10), 32(10), 30(10), 30(11): 57(13). Mesoscutum (Fig. 71) meshlike reticulate, the reticulations somewhat larger medially than laterally, and with inconspicuous white setae; notaulus extending from spiracle as curved furrow on inclined anterior surface, its posterior limit dorsally contiguous with posterior limit of anteroadmedian line and together extending posteriorly as obscure line of smaller reticulations; parapsidal line a distinct region of microsculpture posterior to spiracle. Axillae elongate-triangular, separated by about 3× own width (Fig. 71). Scutellum low convex, about 1.2× as long as wide; meshlike reticulate similar to mesoscutum, the reticulations about same size as on lateral lobe (Fig. 71); with inconspicuous white setae. Mesopleuron with exposed, bare lower mesepimeron; acropleuron very shallowly meshlike reticulate near tegula but mostly meshlike coriaceous anterior to oblique microsculptured region, and longitudinally coriaceous-alutaceous posteriorly. Fore wing with cc: mv: stv: pmv = 54: 33: 10: 13; basal cell entirely setose; cubital area bare except near mediocubital fold and closed by setae along posterior margin over about apical half; disc uniformly setose except for short region along mediocubital region just beyond basal fold. Metacoxa with relatively short and quite sparse setae along dorsal and ventral margins, with outer surface broadly bare except for line of setae along basal margin. Propodeum with callus comparatively sparsely setose to posterior margin; bare anteriorly between spiracle and foramen. Gaster (Figs 17, 36) about 2.7× as long as mesosoma, with inconspicuous hairlike setae dorsally and laterally, the setae whitish basally but dark apically; posterior margin of penultimate tergum clearly not extending to level of cerci, the precercal portion equal in length to about half distance between cerci; syntergum with medial length measured to apex of penultimate tergum almost 6× transcercal width, conspicuously compressed posterior of level of cerci, and almost 1.5× as long as penultimate tergum.

###### MALE

(Fig. 44). ALLOTYPE: length about 4.1 mm. Similar to holotype except as follows. Color. Head with arm of M-like coppery region on upper face ventrally contiguous with inner orbit, and lower face more distinctly coppery; tegula dark; mesoscutal lateral lobe above prepectus more reddish-coppery similar to dorsomedial region; legs (Fig. 44), including tarsi, much more extensively dark, with only knees of front and middle legs narrowly, apex of metafemur narrowly, and base of basal segment of pro- and metatarsus yellowish.

*Structure/setation.* Head in dorsal view about 1.8× as wide as long, with IOD about 0.44× head width; IOD: MPOD: OOL: POL: LOL = 60: 15: 10: 18: 11; in frontal view about 1.2× as wide as high, with dorsal margin of torulus at level of lower orbits; malar space about 0.57× eye height. Antenna (Fig. 44) with flagellum less conspicuously elongate-slender, with length of flagellum + pedicel about 1.78× width of head; scape: pedicel: fu1–fu8: clava = 69(20): 30(10): 15(8), 28(10), 28(10), 26(10), 22(10), 22(10), 20(10), 20(10): 50(10). Fore wing with cc: mv: stv: pmv = 57: 31: 10: 12. Propodeal callus setose only near anterior margin except for a couple of setae lateral to and posterior to spiracle.

##### Variation.

Females vary in length from about 4.5–6 mm and males from about 3.8–4.2 mm. Females and males have a similar color pattern to the holotype and allotype though extent and intensity of the brown regions of the middle and hind legs are variable, the metafemur sometimes with up to about the apical half yellowish-orange and the metatibia sometimes being more or less entirely yellowish-orange. Intensity of the coppery region on the upper face in females is also somewhat variable, the upper arms sometimes not quite extending to the posterior ocelli or sometimes almost filling the interocellar triangle. Some females have the dorsomedial mesoscutal region dark without a distinct coppery luster, but the general color pattern is similar to that described for the holotype. Also, the combined notauli/anteroadmedian lines are often slightly depressed as well as having smaller reticulations and therefore are visible, though relatively obscurely so, as parallel paramedial lines over about the anterior two-thirds of the mesoscutum.

##### Biology.

Primary parasitoid of Agrilus coxalis Waterhouse (Coleoptera: Buprestidae) associated with Quercus emoryi (Emory oak).

##### Distribution.

**Map 3.**
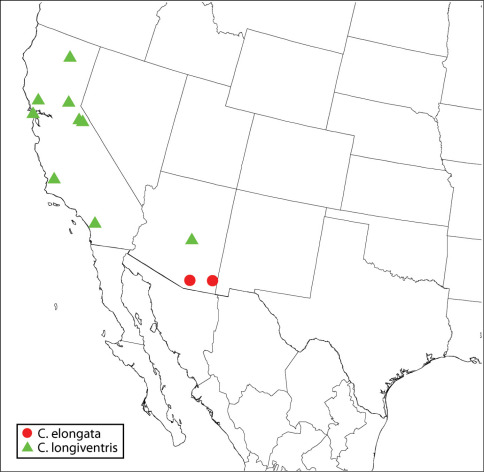
Distribution of Calosota elongata and Calosota longiventris.

Southwestern USA (Arizona)(Map 3), but undoubtedly also at least Mexico and possibly south to Guatemala along with its known host.

##### Recognition.

Because of a conspicuously elongate-slender gaster and syntergum, females of Calosota elongata are most similar to those of Calosota longiventris. However, the syntergum of Calosota elongata always has a distinct precercal portion, whereas this is usually lacking from Calosota longiventris because the penultimate tergum normally extends to or slightly posterior to the level of the cerci. Females of Calosota elongata also have much longer flagellomeres, a more distinctly bicolored mesoscutum (cf. Figs 17, 18), the middle and hind legs always partly brown (cf. Figs 36, 37), and the fore wings hyaline (cf. Figs 36, 37). Males of Calosota longiventris differ from those of Calosota elongata most conspicuously by their shorter flagellomeres and more uniformly colored mesoscutum, but also by a more uniformly yellowish-orange middle leg (cf. Figs 44, 45).

#### Calosota longivena sp. n.

urn:lsid:zoobank.org:act:1CC22E89-9B97-4373-A5FA-E5679A15D298

Figs 4 15 35 62 

##### Etymology.

From the Latin words longus, ‘long’ and vena, ‘pipe’, in reference to the long marginal vein relative to the stigmal vein of females compared to Calosota aestivalis.

##### Type material.

HOLOTYPE ♀ (CNC type no. 23926). USA: TX [Texas]; Brazos Co., Lick Creek Park, College Sta., 18.XII [“I” added in ink after printed XII].1987, J.B. Woolley; CNC Photo 2009-26; Holotype Calosota longivena Gibson.

Paratypes. **USA**. ***Florida***: Dade Co., Homestead, IFAS Exp. Sta., 9.XI.73, W.H. Pierce (1♀ FSCA). Monroe Co., Middle Torch Key, 1–30.XI.86, S&J Peck, hammock forest edge (1♀ CNC). Pinellas Co., St. Petersburg, 18.XI.64, E.R. Simmons (1♀ FSCA). ***Mississippi***: Boliver Co., Dahomey Natl. Wildlife Refuge, Hwy 446, 19 km. W Boyle, 33°42'N; 90°56'W, 1–15.IX.98, N.M. Schiff (1♀ UCDC, CNC Photo 2009-42); 12 mi. W Boyle on 446, 27.VIII–20.IX.09, N. Schiff & E. Green (1♀ CNC). Washington Co., nr Stoneville Delta Exp. Forest, 33°28'N; 90°54'W, 17–31.VIII, 1–15, 5–26.IX.98 (3♀ UCDC), 33°27'N; 90°55'W, 1–15.IX.98 (1♀ UCDC). Texas: Brazos Co., College Station, Lick Creek Pk, 11–18.X.1987, R. Wharton, Mal. tr. (1♀ CNC, CNC Photo 2009-31). Kerr Co., Kerrville, 12.V.1988 H. & M. Townes (1♀ AEI). Montgomery Co., Sam Houston National Forest, 10.X.65 (1♀ TAMU). Terrell Co., Sanderson, 27.IV.59, Becker & Howden (1♀ CNC).

##### Description.

###### FEMALE

(Figs 15, 35). HOLOTYPE: length about 5.1 mm. Color. Head (Fig. 4) primarily dark but with slight purple, bluish or greenish lusters under some angles of light, including obscure greenish spot below anterior ocellus. Maxillary and labial palpi, antenna, and tegula dark. Mesoscutum and scutellar-axillar complex dark (Fig. 15) with slight purple, bluish or greenish lusters under different angles of light similar to head. Acropleuron dark with slight metallic lusters similar to mesonotum under some angles of light. Legs (Fig. 35) with femur and tibia of front leg extensively dark brown, but trochantellus, knee, tibia apically, and tarsus mostly yellowish; middle leg with trochantellus yellow, femur brown except apically, tibia mostly yellowish with more brownish tinge subbasally and more distinctly yellow basally and apically, and tarsus yellow; hind leg similar to middle leg except trochantellus yellow only ventrally and femur darker brown. Fore wing hyaline; setae uniformly brown. Gaster (Figs 15, 35) dark brown.

*Structure/setation.* Head in dorsal view about 2× as wide as long, with IOD about 0.35× head width; IOD: MPOD: OOL: POL: LOL = 60: 14: 8: 18: 15; in frontal view about 1.2× as wide as high, with dorsal margin of torulus at level of lower orbits; malar space about 0.57× eye height. Head (Fig. 4) with frontovertex and parascrobal region meshlike reticulate to about level of dorsal limit of interantennal region, medially the reticulations tapered ventrally between dorsal limits of smooth and shiny scrobes; lower parascrobal region and interantennal region more meshlike coriaceous-reticulate; clypeal region microcoriaceous and paraclypeal region obliquely alutaceous-reticulate. Head with brownish setae on frontovertex and more conspicuous white setae on parascrobal region, interantennal region and lower face. Antenna (Figs 15, 35) with flagellum clavate; length of flagellum + pedicel about 1.6× head width; scape: pedicel: fu1–fu8: clava = 78(17): 30(11): 13(9), 22(10), 22(10), 23(10), 22(10), 19(10), 19(11), 18(12): 52(16). Mesoscutum (Fig. 15) meshlike reticulate, the reticulations somewhat larger medially than laterally where more similar to vertex, and with whitish, hairlike setae; anteroadmedian lines and notauli obscurely developed on inclined anterior surface and not extending posterodorsally; parapsidal lines more distinct lines of microsculpture. Axillae elongate-triangular, separated by about 3.5× own width. Scutellum low convex, about 1.15× as long as wide (dorsellum covering frenal area); meshlike reticulate, the reticulations anteromedially similar to lateral lobes but more elongate-reticulate laterally and posteriorly; with white setae. Mesopleuron with exposed, bare lower mesepimeron (cf. Fig. 55); acropleuron very shallowly and inconspicuously reticulate anterodorsally near tegula, but mostly meshlike coriaceous to obliquely coriaceous-alutaceous anterior to oblique microsculptured region and longitudinally coriaceous-alutaceous posteriorly. Fore wing (Fig. 62) with cc: mv: stv: pmv = 61: 41: 10: 14, and perpendicular distance between apex of stigmal vein and anterior margin of wing 1.1× length of stigmal vein; basal cell entirely setose; cubital area bare except anteriorly along mediocubital fold, including along posterior margin; disc uniformly setose, without distinct bare band adjacent to basal fold. Metacoxa setose along dorsal, ventral and basal margins, but outer surface broadly bare mediolongitudinally. Propodeum with callus setose to posterior margin; bare anteriorly between spiracle and foramen. Gaster (Figs 15, 35) about 2.2× as long as mesosoma; with white setae, the setae denser and more conspicuous laterally than dorsally; penultimate tergum with posterior margin extending to level of cerci; syntergum with medial length about 2.6× transcercal width, obviously compressed beyond cerci, and subequal in length to penultimate tergum.

###### MALE.

Unknown.

##### Variation.

Females vary in length from about 3.7−5.1 mm. The head sometimes has a more distinct green spot below the anterior ocellus and/or a reddish-coppery luster along the inner orbit ventral to the posterior ocellus, but only two specimens from Texas (Kerr, Sanderson) of 14 females have an obvious M-like coppery region below the anterior ocellus. The scape sometimes has a slight greenish luster, the mesoscutum has variably distinct reddish, bluish-green or purple lusters, and the meso- and metatibiae are sometimes more extensively dark brown medially than for the holotype. The marginal vein varies from about 3.6−4.2× the length of the stigmal vein, and fu1 from about 1.3−1.8× as long as wide. The penultimate tergum sometimes extends quite distinctly beyond the level of the cerci, and the syntergum varies from about 1.8−2.7× the transcercal width.

##### Biology.

Unknown.

##### Distribution.

Nearctic: southern USA east of New Mexico (Map 2).

##### Recognition.

Except for a comparatively longer marginal vein (cf. Figs 60, 62) and usually the absence of a differentiated M-like region on the upper face (cf. Figs 2, 4), females of Calosota longivena are very similar to females of Calosota aestivalis that do not have obviously differentiated paramedial longitudinal bands on the mesoscutum (e.g. Fig. 14). The fore wing (Fig. 62) is also hyaline and setose such that a distinct bare band adjacent to the basal fold is lacking, the stigmal vein is obtusely angled relative to the marginal vein so that the perpendicular distance from the apex of the stigmal vein to the wing margin is subequal to its length, the stigma is elongate-slender and evenly curved from the stigmal vein, fu1 is always less than twice as long as wide, and the syntergum is moderately elongate-slender. However, all of these latter characteristic features of female Calosota longivena appear to be variable among females I identify as Calosota aestivalis (see further under latter species).

#### Calosota longiventris

(Ashmead)

Figs 6 18 31, 37 45

Calosoter longiventris Ashmead 1896: 12–13. Lectotype ♀ (USNM, type no. 3463; examined), designated by Burks 1973: 30.
Calosota longiventris ; Peck 1951: 474.

##### Description.

###### FEMALE

(Figs 18, 37). Length about 4.6–8.2 mm. Color. Head dark with limited green luster to largely green dorsally to entirely greenish or bluish-purple, except usually with variably distinct, dark to coppery region on vertex (when apparent region forming complete band between inner orbits or reduced to variably large and conspicuous regions adjacent to upper inner orbit and behind ocellar triangle), and usually with more or less M-like dark or coppery region (region sometimes narrowly divided below anterior ocellus, Fig. 6), the lateral arm of region sometimes extending dorsally to posterior ocellus lateral to anterior ocellus and ventrally toward but not distinctly merging with inner orbit; lower face often with slight coppery luster toward oral margin under some angles of light; back of head dark or greenish with slight coppery luster to bluish-purple under some angles of light but at most only obscurely differentiated ∩-shaped band along outer orbit and occiput. Maxillary and labial palpi dark. Antenna dark brown except scape often and pedicel sometimes with slight metallic luster. Tegula dark. Mesoscutum (Fig. 18) uniformly dark to dark greenish or bluish-green similar to head except notauli, anteroadmedian lines and parapsidal lines sometimes coppery or at least differentiated by a slightly different color and/or mesoscutum with a relatively obscure longitudinal dark or coppery band adjacent to combined notauli/anteroadmedian line; scutellar-axillar complex similar in color to most of mesoscutum or, more commonly, most of scutellum other than frenal area with more distinct coppery luster than any longitudinal bands of mesoscutum. Acropleuron dark brown to greenish or bluish-purple, the microsculptured region without distinct coppery luster. Legs sometimes dark except knees, extreme apices of tibiae, and tarsi extensively yellowish, but middle and hind (less commonly front leg) sometimes almost uniformly yellowish-orange (Fig. 37), though mesofemur often more distinctly orange compared to somewhat lighter tibia and metafemur sometimes with up to about basal half brownish or with slight metallic luster. Fore wing hyaline or variably distinctly brownish behind venation from about level of parastigma to apex of stigmal vein, often only anterior to mediocubital fold or also with longitudinally on convex portion of fold, but sometimes completely to hind margin excluding longitudinal folds; setae uniformly brown. Gaster (Figs 18, 37) sometimes entirely dark brown, more commonly brown dorsally with basal terga basolaterally and apical terga more extensively bluish-purple.

*Structure/setation.* Head in dorsal view about 1.9–2.1× as wide as long, with IOD about 0.36–0.40× head width, LOL usually slightly greater than OOL with OOL about 0.75× and LOL about 0.75–1× MPOD, and POL about 1.25–1.7× MPOD; in frontal view about 1.2–1.3× as wide as high, with dorsal margin of torulus at level of lower orbits; malar space about 0.6–0.7× height of eye. Head (Fig. 6) with frontovertex and parascrobal region meshlike reticulate to about level of dorsal limit of interantennal region, medially the reticulations tapered ventrally between dorsal limits of smooth and shiny scrobes; lower parascrobal region and interantennal region much shallower meshlike reticulate to coriaceous-reticulate; clypeal region microcoriaceous and paraclypeal region obliquely reticulate-alutaceous. Head with dark setae on frontovertex and more conspicuous white setae on parascrobal region, interantennal region and lower face. Antenna with scape about 4.7–5.2× as long as wide; pedicel about 2.9–3.3× as long as wide; length of flagellum + pedicel about 1.45–1.6× head width; flagellum not conspicuously clavate, the funiculars subequal in width and clava only slightly wider than funicle; combined length of fu1 + fu2 about 1–1.2× as long as pedicel; fu1 about 1–1.9× as long as wide; subsequent funiculars all longer than wide with fu2 about 2–2.5× and fu8 about 1.2–1.6× as long as wide; clava often slightly collapsed, but about as long as apical 2–2.75 funiculars. Mesoscutum (Fig. 18) meshlike reticulate, the reticulations usually somewhat larger medially than laterally, with inconspicuous white setae; notaulus extending from spiracle as curved furrow on inclined anterior surface, its posterior limit contiguous dorsally with posterior limit of anteroadmedian line and together extending posteriorly (sometimes for almost entire length of mesoscutum) as very slightly concave parallel lines of much smaller reticulations (usually obscure unless differentiated also by color); parapsidal line usually quite a distinct region of microsculpture. Axillae elongate-triangular, separated by about 3.5–5× own width. Scutellum low convex, about 1.2× as long as wide; meshlike reticulate, the reticulations similar in size or smaller than on mesoscutum laterally and more elongate, at least medially; with inconspicuous white setae. Mesopleuron with exposed, bare, lower mesepimeron (cf. Fig. 55); acropleuron of smaller individuals sometimes meshlike coriaceous anteriorly but usually variably extensively though shallowly meshlike reticulate anteriorly, the sculpture becoming more coriaceous to obliquely coriaceous-alutaceous anterior to oblique microsculptured region and very finely, longitudinally coriaceous-alutaceous posteriorly. Fore wing with cc: mv: stv: pmv about 41–50: 22–25: 10: 12–15; basal cell entirely setose; cubital area bare except sometimes anteriorly along mediocubital fold and closed by setae along posterior margin over about apical third to half; disc setose except for short region of mediocubital fold just beyond basal fold or more distinct, often arcuate bare band along basal fold and mediocubital fold, the bare region sometimes separated from costal cell by line of setae but usually contiguous with costal cell. Metacoxa setose along dorsal, ventral and basal margins, and sometimes outer surface with up to about basal half sparsely setose. Propodeum with callus setose to posterior margin; bare anteriorly between spiracle and foramen. Gaster (Figs 18, 37) about 2.3–3× as long as mesosoma; more or less uniformly covered with white, hairlike setae; posterior margin of syntergum usually extending to or slightly beyond level of syntergum; syntergum about 3.9–5.3× as long as transcercal width, obviously compressed beyond level of cerci, and about 1.6–2.5× as long as penultimate tergum.

###### MALE

(Fig. 45). Similar to female except as follows. Length about 2.6–4.1 mm. Color. Fore wing hyaline and middle leg usually more or less uniformly yellowish-orange beyond mesocoxa, the mesofemur sometimes slightly brownish and metafemur extensively brown with only knee or at most up to about apical quarter yellowish (Fig. 45); mesoscutum distinctly bluish or bluish-green except sometimes with more greenish or greenish-coppery paramedial bands in region of notauli/anteroadmedian lines, and scutellum dark with coppery luster.

*Structure/setation.* Antenna with scape and pedicel both about 3× as long as wide; length of flagellum + pedicel about 1.3–1.4× as long as head width; fu1 slightly widened distally and only about as long as maximum width; fu2 about 1.3–1.5× as long as wide; fu8 about 1.2–1.3× as long as wide; and clava about equal in length to combined length of apical 2.5 funiculars. Fore wing with cc: mv: stv: pmv = 43–47: 22–24: 10: 10–11. Propodeum sometimes setose anteriorly only to level about equal with posterior margin of spiracle, and then often with 1 or 2 setae behind spiracle.

##### Biology.

Unknown, but very probably a parasitoid of some wood-boring beetle.

##### Material examined.

**USA**. ***Arizona***: Parker Cr., Sierra Ancha, 20.IV.47, H&M Townes (1♀ AEIC). ***California***: Santa Cruz Mts (♀ lectotype, 1♂ paralectotype). Marin Co., Mt. Tamalpais Watershed Area, Pine Mountain Fire Rd, 1100’, 11.VII.04, R.L. Zuparko, ex. Quercus agrifolia (1♀ RLZC). Mono Co., Sonora Pass, 11.VIII.73, L. Lacey (1♀ FSCA). Napa Co., E slope Mt. St. Helena, 1.4 mi. NE summit Hwy 53, VIII.66, H.B. Leech, emerged from cone picked from living Pinus attenuata (7♀, 1♂ CASC, CNC Photo 2009-40, 2009-23, CNC SEM 2009-44). Placer Co., 17.5 mi. E of Foresthill, Mosquito Ridge Rd, 4150’, 11, 12, 14.IV, 20.V, 1, 29, 30.VI, 7, 13, 15, 21.VII, VII.66 (21♀, 3♂ CASC), 15.II, 16.IV, 24.VI.67 (2♀, 1♂ CASC, CNC Photo 2009-21, 2009-22), H.B. Leech, emerged from cone picked from living Pinus attenuata. Riverside Co., Menifee Valley, hills on W end, 33°39'N; 117°13'W, 1800’, 1.VII–1.VIII.95, J.D. Pinto (1♀ UCRC). San Luis Opisbo Co., 6 mi. SE Pozo, R16E, T315, sects. 4–5, 1500’, 2.IV–4.V.89, W.E. Wahl (1♀ CNC). Shasta Co., Cayton, 14.VII.18, E.P. VanDuzee (1♀ EMEC). Tuolumne Co., Stanislaus Natl For., Clark Fork Rd, 1 mi. N Hwy 106, 38°21'50N; 119°52'20W, 556’, 12.VII.06, S. Fullerton, E. Zoll, S. Kelly, R.P. Russell, Manzatia scrub (21♂ UCFC).

##### Distribution.

Nearctic: southwestern USA (Map 3).

##### Remarks.

As stated by Burks (1973), the lectotype of Calosota longiventris is “fragmentary”, including about the apical half of the syntergum broken off and glued to the plastic point, to which separately are glued the remainder of the metasoma, leg parts, and the mesosoma plus head. The head lacks the left antenna beyond fu2 and the right antenna beyond fu6. However, the body parts remaining are sufficient to readily place the name, including the relatively short funiculars [fu1–fu6 = 13(9), 26(10), 27(10), 29(10), 29(10), 21(10)], conspicuously long syntergum, and yellowish-orange middle and hind legs. As noted by Burks (1973), the remaining fore wing is hyaline; additionally, the lectotype is one of few females observed with a line of setae separating the cubital area from the short bare region of the convex part of the mediocubital fold (most setae abraded but setal pattern indicated by sockets). The male paralectotype from the same locality has the same setal pattern, but I identify the male paralectotype from Argus Mts as a male of Calosota aestivalis based on leg color.

##### Recognition.

Females of Calosota longiventris are most similar to those of Calosota elongata, as discussed under the latter species. A few females of Calosota longiventris do not have the penultimate tergum extending to the level of the cerci so that there is a visible precercal portion of the syntergum similar to Calosota elongata, but this likely is an artifact of preservation in these very few females. The single female from Arizona (Map 3) is unusually dark, lacking distinct metallic lusters except for on the interantennal region and scape, and having the legs except the tarsi mostly dark, but in other features is similar to typical Calosota longiventris from California. If males of Calosota longiventris from Arizona also have mostly dark legs, unlike those from California, they will closely resemble males of Calosota aestivalis. However, males of Calosota aestivalis have the ventrolateral arm of the coppery region below the anterior ocellus quite obviously contiguous with the inner orbit (Fig. 2), whereas typical males of Calosota longiventris from California have the arm separated from the inner orbit (cf. Fig. 5) and it is possible that dark males from Arizona may even lack the coppery region. Males of Calosota aestivalis also commonly have fu1 quite distinctly elongate, whereas fu1 is quadrate to slightly transverse in males of Calosota longiventris.

#### Calosota metallica

(Gahan)

**Figures 7−12.**
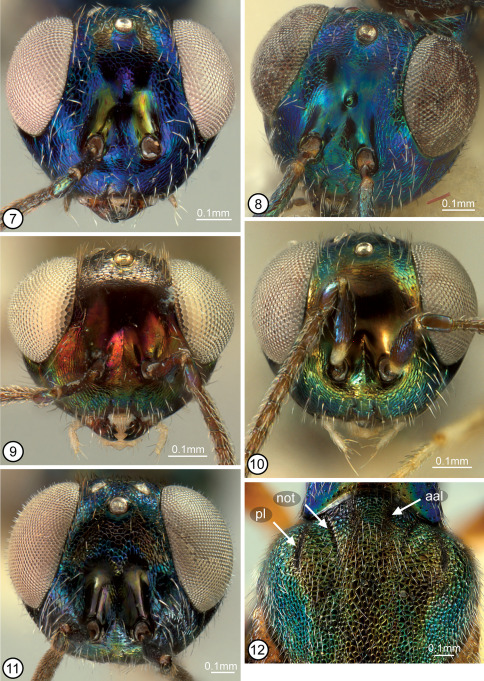
**7-11** Head ♀, frontal view: **7** Calosota metallica (27) **8** Calosota grylli (lectotype) **9** Calosota albipalpus (9) **10** Calosota setosa (holotype) **11** Calosota bicolorata (holotype) **12** Calosota acron ♀ dorsal mesoscutum (33). Numbers in parentheses = CNC Photo 2009-no. Abbreviations: **aal** = anteroadmedian line, **not** = notaulus, **pl** = parapsidal line.

**Figures 13−21.**
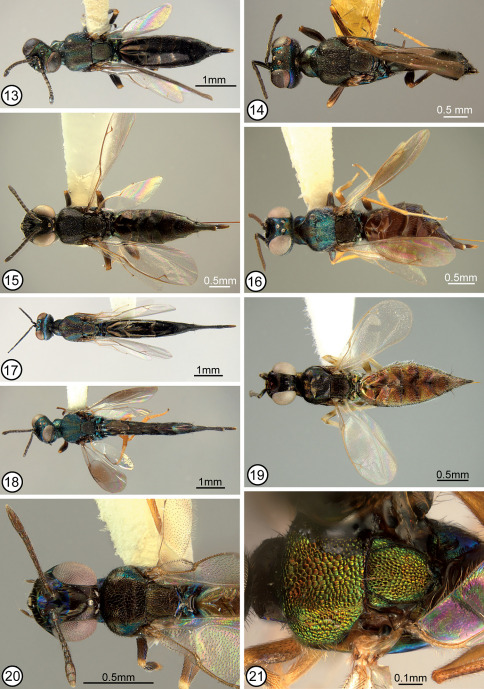
**13−19** dorsal ♀ habitus: **13** Calosota aestivalis (20) **14** Calosota kentra (holotype) **15** Calosota longivena (holotype) **16** Calosota bicolorata (holotype) **17** Calosota elongata (24) **18** Calosota longiventris (40) **19** Calosota albipalpus (8) **20** Calosota speculifrons ♀, head and dorsal mesosoma (5) **21** Calosota panamaensis ♀, dorsal mesosoma (holotype). Numbers in parentheses = CNC Photo 2009-no.

Figs 7 22 41 59, 63 68 79, 80

Calosoter metallicus Gahan 1922 (May 25): 16–17. Holotype ♀ (USNM, type no. 24988; examined, antenna mounted on slide), by original designation.
Calosota (Paracalosota) viridis Masi 1922 (November 30): 142–144. Type data: Italy: Tuscany, Giglio Is. Syntypes, ♀ (MCSN; not examined). **syn. n.**
Calosota metallica ; Packard 1928: 14.
Calosota matritensis Bolívar y Pieltain 1929: 140–142. Type data: Spain: Madrid Prov., Chamartín. Holotype ♀ (MNCN; examined), by original designation. Synonymy with Calosota viridis by Askew and Nieves Aldrey 2006: 95. **syn. n.**
Calosota coerulea  Nikol’skaya, 1952: 483/497. Type data: USSR: Tadzhikistan; parasitic on Harmolita species in wheat stems. Holotype ♀ (ZMAS; not examined), by monotypy. Synonymy with Calosota viridis by Bouček 1970: 79. **syn. n.**

##### Description.

###### FEMALE

**Figures 22−29.**
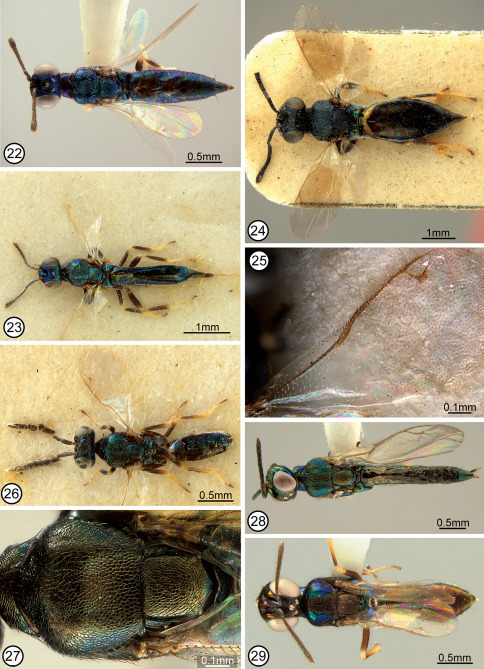
**22−24** dorsal ♀ habitus: **22** Calosota metallica (29) **23** Calosota grylli (lectotype) **24** Calosota lixobia (paralectotype 6581). **25−26** Calosota modesta ♂ (holotype): **25** right fore wing **26** dorsal habitus **27** Calosota vernalis ♀, dorsal mesosoma (43) **28−29** dorsal habitus: **28** Calosota acron ♀ (13) **29** Calosota speculifrons ♂ (allotype). Numbers in parentheses = CNC Photo 2009-no.

**Figures 30−38.**
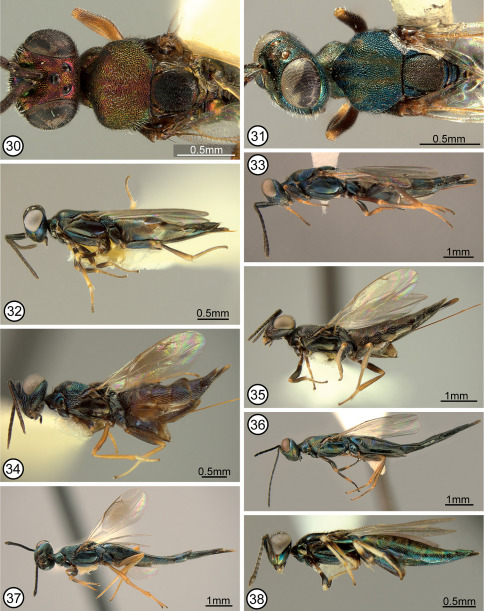
**30−31** head and dorsal mesosoma ♂: **30** Calosota bicolorata (allotype) **31** Calosota longiventris (23) **32−38** lateral ♀ habitus: **32** Calosota vernalis (43) **33** Calosota aestivalis (16) **34** Calosota bicolorata (holotype) **35** Calosota longivena (holotype) **36** Calosota elongata (24) **37** Calosota longiventris (21) **38** Calosota albipalpus (7). Numbers in parentheses = CNC Photo 2009-no.

(Figs 22, 41). Length about 1.5–3.7 mm. Color. Head of small specimens sometimes brownish with variably extensive metallic lusters, but normally (Fig. 7) bright green to blue or purple except usually with dark, nonmetallic or slight coppery band extending from each scrobe dorsally to side of anterior ocellus and sometimes to posterior ocellus and/or sometimes narrowly contiguous around posterior margin of anterior ocellus. Maxillary and labial palpi dark. Antenna dark brown with scape and pedicel usually with similar metallic luster as head, and scape often with extreme base or rarely up to about basal two-thirds yellowish. Mesosoma (Figs 22, 41) similar in color to head, including entire scutellum, the frenal area not differentiated from rest of scutellum by color. Legs usually (Fig. 41) with femora and tibiae extensively dark except knees, tibiae variably extensively apically, and all but one or two apical tarsomeres yellowish-orange to white, but middle and hind legs sometimes more extensively, rarely entirely, yellowish-orange except knees, tibiae apically and tarsi more distinctly white. Fore wing (Fig. 59) hyaline; setae uniformly colored, sometimes white but usually at least slightly yellowish to brown. Metasoma (Figs 22, 41) often similar in color to head and mesosoma but more commonly more or less brown, at least dorsally, with variable metallic lusters.

*Structure/setation.* Head in dorsal view about 1.7–2× as wide as long, with IOD about 0.37–0.47× head width, OOL about 0.5–1.2x, LOL about 1.3–1.8x, and POL about 1.6–2.5× MPOD; in frontal view about 1.1–1.2× as wide as high, with dorsal margin of torulus about at level of lower orbits; malar space about 0.56–0.64× height of eye. Head (Figs 7, 68) more or less uniformly sculptured except for smooth and shiny scrobes and microcoriaceous clypeal region, the frontovertex and often parascrobal region meshlike coriaceous in smaller specimens to distinctly meshlike reticulate or alutaceous-reticulate in larger specimens, and lower face more obliquely coriaceous-alutaceous to alutaceous-reticulate. Head with white setae except for bare scrobal depression. Antenna (Figs 41, 80) with scape about 3.9–5.5× as long as wide; pedicel about 2–2.3× as long as wide, at least a little longer than combined length of fu1+fu2 and sometimes about as long as fu1–fu3; flagellum conspicuously clavate, with length of flagellum + pedicel about 1–1.1× as long as width of head; smaller specimens sometimes with fu1 strongly transverse (ringlike) and fu2 quadrate to only slightly longer than wide, but larger specimens with fu1 quadrate to slightly longer than wide, subsequent funiculars oblong basally and shortened apically to slightly transverse fu8; clava usually quite distinctly bulbous, at least 2.5× as wide as fu2 even when not compressed, and about as long as apical 3–4 funiculars. Mesoscutum (Figs 22, 79) meshlike reticulate at least medially, usually somewhat more shallowly reticulate to coriaceous laterally on lateral lobes, with variably conspicuous white setae; notauli variably distinct but often obscure, anteroadmedian lines extremely obscure or not apparent, and parapsidal lines more distinct microsculptured regions. Axilla very slender, separated by at least 5× own width (Fig. 79). Scutellum usually quite conspicuously convex, about 1–1.2× as long as wide; elongate reticulate to reticulate-strigose except frenal area meshlike reticulate (Fig. 79); with white setae. Mesopleuron with exposed, though sometimes very small and inconspicuous, bare lower mesepimeron; acropleuron sometimes shallowly meshlike reticulate near tegula but more coriaceous-alutaceous anterior to oblique microsculptured region and very finely, longitudinally coriaceous-alutaceous posteriorly. Fore wing (Fig. 59) with cc: mv: stv: pmv about 52–67: 41–50: 10: 10–13; basal cell entirely setose; cubital area bare and open along posterior margin at least to level of posterior margin of basal fold; disc setose except for comparatively broad, oblique bare band contiguous with basal fold and with parastigma to base of marginal vein, though often with one or a few scattered setae within bare region and sometimes closed by setae posteriorly, though usually bare region at least narrowly contiguous with cubital area. Metacoxa setose dorsally and ventrally but broadly bare mediolongitudinally. Propodeum (Fig. 79) with callus setose to posterior margin; usually bare but rarely with single white seta anteriorly between spiracle and foramen (Fig. 79). Gaster (Figs 22, 41) about 1.4–1.7× as long as mesosoma; with white setae, the setae somewhat denser and more conspicuous laterally than dorsally; posterior margin of penultimate tergum extending to or slightly beyond level of cerci; syntergum a more or less equilateral triangle in dorsal view, about 0.8–1× transcercal width, uniformly convex, and about 0.7–0.9× as long as penultimate tergum.

###### MALE

(based on only 4 individuals). Similar to female except as follows. Length about 2.2–2.8 mm. Color. Head and body quite bright green to bluish-purple except for legs; legs with femora and tibiae extensively dark, but knees and apices of tibiae distinctly yellow and tarsi mostly yellow.

*Structure/setation.* Scape more robust, about 3.5–3.6× as long as wide; pedicel shorter, about 1.5–2× as long as wide; combined length of flagellum + pedicel about 1.2× as long as width of head; flagellum (Fig. 63) robust-filiform, with differentiated clava but at most only very slightly widened distally (clava sometimes appearing distinctly wider than flagellum if collapsed and compressed), and conspicuously, densely setose with curved setae much shorter than width of flagellomere; fu1 strongly transverse (ringlike); fu2 sometimes only about as long as wide but at least twice as long as fu1; subsequent funiculars usually all clearly longer than wide but one or two apical funiculars sometimes subquadrate; clava almost as long as combined length of apical 3 funiculars. Fore wing with cc: mv: stv: pmv about 47–56: 35–40: 10: 09–12. Propodeal callus setose anteriorly only to level about equal with posterior margin of spiracle.

##### Biology.

A primary parasitoid of Cecidomyiidae (Diptera), including the Hessian fly, Mayetiola destructor (Say) (Gahan 1933), and species of Tetramesa (Hymenoptera: Eurytomidae), including the wheat strawworm, Tetramesa grandis (Riley) and the wheat jointworm, Tetramesa tritici (Fitch), or a secondary parasitoid through Eurytoma parva Phillips (Eurytomidae) and Ditropinotus aureoviridis Crawford (Hymenoptera: Torymidae) (Chamberlin 1941) in Poaceae and Tamaricaceae (Noyes 2003). Based on comparison of the number of males seen relative to females, the species appears to be primarily parthenogenetic in North America but not in Europe.

The strawberry leaf roller, Ancylis comptana (Frölich), and the oblique banded leaf roller, Choristoneura rosaceana (Harris) (Lepidoptera: Tortricidae) were also listed as hosts in Noyes (2003), but both of these are incorrect. The records are based on Peck (1963) who cited Knowlton and Harmston (1939) as the source, but these authors listed only “wheat jointworms” as the host of Calosota metallica and the former two host names were listed for Catolaccus aeneoviridis (Girault) (Pteromalidae), which follows Calosota metallica in their list of chalcid species.

##### Regional material examined

**Map 4.**
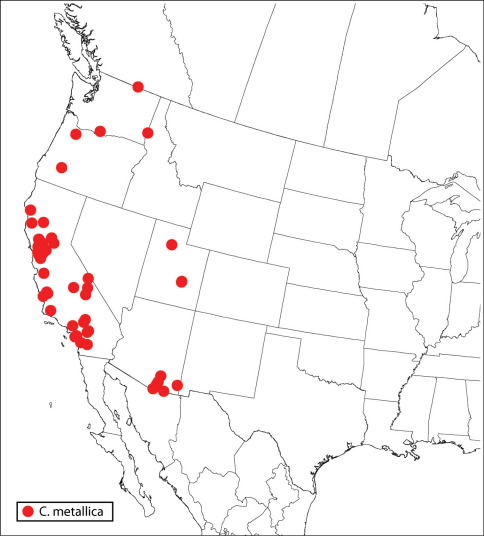
Regional distribution of Calosota metallica.

(Map 4). **CANADA**. ***British Columbia***: 10 mi. E Osoyoos, 30.VII.80, G. Gibson, sweeping Pinus ponderosa forest meadow (1♀ CNC). **USA**. ***Arizona***: Cochise Co., Huachuca Mts, 5354 Ash Cyn Rd, 5 m. NW Hwy 92, 5100’, 1–30.VI.94, N. McFarland (1♀ CNC); Entrance to Pinery Cyn, Chiricahua Mts, 4500’, 22.VIII.83, M. Sharkey (1♀ CNC, CNC SEM 2009-30). Pima Co., Madera Cyn, Bog Springs Campground, 28.VIII.82, J. LaSalle (1♀ UCRC); Santa Catalina Mtns., Molino Basin, 4300’, 2–4.VIII.82, G.A.P. Gibson (1♀ CNC). Santa Cruz Co., Pena Blanca Lk., 1.0 mi. S, 4100’, 6.VIII.82, G.A.P. Gibson (8♀ CNC), 9 mi. W, 4100’, 12.VIII.83, R. Anderson (3♀ CNC); Sycamore Cyn, Hank and Yank Springs, 4200’, 7–8.VIII.82, G.A.P. Gibson (38♀ CNC, 2♀ BMNH, 5♀ AEIC). ***California***: Alameda Co., Sunol, 5.VI.93, R.L. Zuparko, swept from low vegetation (2♀ RLZC); Oakland, Anthony Chabot Regional Pk near Parkridge Gate, 12.VII.02, R.L. Zuparko, ex Umbellularia californica (1♀ RLZC). Contra Costa Co., Bear Creek Rd at Happy Valley Rd, 17.VI.93, R.L. Zuparko, swept from grass and low vegetation (5♀ RLZC); E of Clayton, Morgan Territory Rd at Shale Cliff Court, 26.V.03, R.L. Zuparko, ex. grass and low forbs (1♀ RLZC); Concord, 9, 20.VII.19 (2♀ USNM), 31.VII.19 (1♀ paratype of Calosota metallica), ex. Mayetiola destructor Say, M.C. Lane; Estrella, 12.VII.16 [other data same as holotype] (♂ allotype of Calosota metallica); Moraga, 14.I, 19.VII, 30.VIII.80 (8♀ CNC), D.C. Denning; Moraga, 19.VI.93, R.L. Zuparko, swept from grass and low vegetation (6♀ RLZC); Tilden Reg. Pk, 15.VIII.82, J.B. Whitfield (1♀ EMEC); Walnut Cr., 5 mi. SE, 3.VIII.60, J. Powell, Foeniculum vulgare (1♀ EMEC). Glenn Co., 5 mi. N Elk Creek, 7.VI.84, J.D. Pinto (1♀ UCRC). Humboldt Co., Garberville, 3.VI.87, R.H. Velton (1♀ CNC). Inyo Co., Eureka Valley, Joshua Flat, 24.V.94, S.L. Heydon, off Encoelia (1♀ UCDC); 31 km. ENE Big Pine, 25.V.94, S.L. Heydon off Encoelia (1♀ UCDC); Saline Valley, Lake, 1060’, 7.VII.76, D. Giuliani (1♀ LACM). Los Angeles Co., Altadena, 22.VIII.89, R.H. Crandall (1♀ LACM). Mendocino Co., 1 mi. W Willits, 1.VIII.92, L.S. & R.B. Kimsey (1♀ UCDC). Napa Co., Lake Hennessey, 11 km. ESE St. Helena, 27.X.90, S. Heydon (1♀ CNC, CNC Photo 2009-29), 10 km. E St. Helena, 7.IX.91, S.L. Heydon, off Heracleum (2♀ UCDC); 6 mi. S of Napa, Duhig Rd, 13.V.81, M.E. Schauff, sweeping streamside veg. (1♀ USNM); Pope Valley, 0.25 km. S Aetana Springs, 5–15.VIII.93, L.S. Kimsey (1♀ USDC); 0.5 km. S Aetna Springs, 26.IX–10.X, 3–14.X.93, L.S. Kimsey, blue oak woods (6♀ UCDC). Orange Co., Laguna Cyn., 12.VII.83 (2♀ UCRC), 31.VII.84 (4♀ UCRC), H. Anderson; Orange City, ElToro Rd, 2 mi. E 133, H. Anderson (1♀ UCRC). Sacramento Co., Citrus Heights, 14.VI.67, A.D. & G.J. Keuter (1♀ EMEC). San Benito Co., Tres Pinos, 23.V.18, wheat containing Mayetiola destructor (1♂ USNM). San Bernardino Co., Adelanto, 3.X.33, C.N. Ainslie (1♀ USNM); 1 mi. N Helendale, 13.VI.79, J. LaSalle (1♀ UCRC); Oak Glen, 5–15.VIII.85, R.E. Wagner (1♀ UCRC); San Bernardino, Mts, 23.V.82, J. Huber (1♀ CNC, CNC Photo 2009-28, CNC SEM 2009-31). San Diego Co., 1 mi. W Bonsall, 8.VIII.79, J. LaSalle (1♀ UCRC); Warner Springs, Agua Caliente Cr., 3100’, 26–28.VIII.80, M. Wasbauer & P. Adams (1♀ CDFA). San Luis Obispo Co., Old Creek Rd, 2.3 mi. E Hwy 1, 30.IV.96, H. Anderson, grasses (3♀ UCRC); San Miguel, 1, 8, 12, 13, 17, 26.VII, 8.VIII.16, C.M. Packard, reared from wheat stems containing Isosoma sp., 1, 8, 13 (holotype), 17, 26.VII, 8.VIII.16, Webster No. 13368, Pasadena No. 16135 (♀ holotype, 6♀ paratypes of Calosota metallica). Santa Barbara Co., 45 km. NW Santa Barbara, Sedgewick Ranch Res., 34°44'N; 120°02'W, 14, 21, 24.V, 24.VI–8.VIII, 13.VIII, 19.IX, 1.X.97, R. Schlinger (17♀ UCDC). Solano Co., Birds Landing, 4.VIII.19, M.C. Lane (1♀ USNM); 21.VI.24, M. Marshall, wheat containing Phytophaga destructor (1♀ USNM); Cold Cyn Rsrv., 11 km. W Winters, 7–17.VI, 20.VI–4.VII, 4–18.VII, 30.VIII–12.IX.90, 29.V–12.VI, 11–24.VII.91, 29.VI–10.VII.92, D. Carmean (20♀ UCDC, CNC Photo 2009-41), 1–15.VIII.94, L.S. Kimsey, live oak woods (1♀ UCDC), 18, 22.VIII.90, 15.V, 24.VII–5.VIII, 2–18.X.91, 17.VII.93, S.L. Heydon (8♀ UCDC), 17.VII.93, S.L. Heydon, Dacus (1♀ UCDC), 13.VIII.91, S.L. Heydon, ex. Dacus pusillus (2♀ UCDC), 15.V, 13, 17.VII.91, 17.V.92, S.L. Heydon, sweeping Dacus pusillus (2♀, 3♂ UCDC, CNC Photo 2009-34), Spring ‘96, S.L. Heydon, ex. dead grass (2♀ UCDC); Rio Vista, 20.IX.20, B.G. Thompson, ex. Isosoma grandis (1 ♀ USNM). Sutter Co., 30 km. N Sacramento, Babelaine Audubon Sanct., Feather R., 38°57'N; 121°35'W, 15.VII.98, L.S. Kimsey (1♀ UCDC). Tulare Co., Ash. Mtn. Pwr. Sta., 18.X.83, J.A. Halstead (2♀ CNC). ***Idaho***: Latah Co., Moscow, 3.IX.39, T.A. Bradley (1♀ USNM). ***Oregon***: Clackamas Co., Mollala, 27.VII.27, Forest Grove No. 26-31Q, T.R. Chamberlin, ex. Harmolita tritici gall (1♀ USNM). Douglas Co., 20 mi. NE Tiller, Umpqua Falls, 16.VII.88, J. LaSalle (1♀ CNC, CNC Photo 2009-27, CNC SEM 2009-32). ***Utah***: Emery Co., Buckskin Spring, nr Goblin Valley, 26.VIII.81, E.E. Grissell, vegetation at spring (1♀ USNM); 3 mi. N Goblin Valley, 30.VIII.81, pondside vegetation (2♀ USNM). Tooele Co., Lake Point, Spring 1930, ex. Harmolita, G.F. Knowlton & M.J. Janes (6♀ USNM). ***Washington***: Klickitat Co., Goldendale, 21.VII.88, J.D. Pinto (2♀ CNC). Pullman, 1908, G.I. Reeves (1♀ USNM).

##### Distribution.

Noyes recorded Calosota viridis from North Africa and several countries in the Palaearctic region; I saw specimens that I identify as Calosota metallica from Bulgaria (BMNH, NMPC), Corsica (ZSMC), Cyprus (USNM), France (BMNH, CNC), Hungary (HNHM), Iran (CNC), Italy (BMNH, CNC, NMPC), Kyrgyzstan (UCRC), Romania (CNC), Sardinia (BMNH, NMPC), Slovakia (NMPC), Spain (BMNH, MNCN, NMPC) and Turkey (CNC). In North America, Calosota metallica occurs in southernmost British Columbia and throughout the USA west of the Rocky Mountains (Map 4), but undoubtedly extends also into northern Mexico. Its restricted distribution west of the Rocky Mountains and apparent close relationship with morphologically similar species in Europe (see below) suggest that it is of European origin and is not naturally Holarctic. Rather, its distribution and biology suggest that it likely was introduced accidentally into the USA, possibly California, by early settlers in straw.

##### Recognition.

Calosota metallica is easily distinguished from other regional species based on its color and the presence of a large, broad, fore wing speculum that is completely bare or has at most a few widely separated setae within an obviously broad bare region that extends to the basal fold and parastigma (Fig. 59).

The apparent similarity between Calosota metallica and Calosota viridis was first noted by Bouček (1970) when he synonymized Calosota coerulea under Calosota viridis after examining type material of the latter two names. The synonymy of Calosota metallica and Calosota viridis was also proposed to me by Dr. Lucian Fusu, Faculty of Biology, “Al. I. Cuza” University, Iasi, Romania, who sent me a female from Romania that he had identified as Calosota viridis. Although I have not examined the syntypic series of Calosota viridis I am confident in formally synonymizing Calosota viridis under Calosota metallica syn. n. based on the original description and figure of the fore wing given for Calosota viridis by Masi (1922, fig. 1), which shows the characteristic broad speculum of Calosota metallica. My synonymy of Calosota coerulea under Calosota metallica syn. n. follows Bouček (1970).

Askew and Nieves-Aldry (2006) synonymized Calosota modesta and Calosota matritensis Bolívar y Pieltain, both described from Spain, under Calosota viridis. Calosota modesta was based on a unique male holotype, which I examined (MNCN Cat. No. 42574), whereas Calosota matritensis was based on a female holotype and male allotype collected in Chamartín and seven female paratypes collected in Vaciamadrid. Askew and Nieves-Aldry (2006) stated that they located the holotype, allotype, and six paratypes of Calosota matritensis in MNCN, but Martín Albaladejo and Izquierdo Moya (2006) reported only two female paratypes remained in MNCN. The MNCN curator of entomology was also able to find only two paratype females in response to my request to examine type material of Calosota matritensis (Mercedes Paris, personal communication). These two paratypes (MNCN Cat. No. 42521 and 42522) and the male holotype of Calosota modesta bear identical data labels with “Vaciamadrid, G. Mercet”. Female Calosota matritensis paratype 42522 is about 2.1 mm in length and has a broad fore wing speculum typical of Calosota metallica. It also has quite a bulbous clava that is about 1.6× as long as wide, about twice as wide as fu8, and about 3.2× as wide as fu2; fu2 is quadrate and the combined length of fu1 + fu2 is only slightly more than 0.6× the length of the pedicel. The second paratype (no. 42521) is slightly larger, about 2.3 mm in length, and has a more or less uniformly tapered (lanceolate) clava that is about twice as long as wide, about 1.4× as wide as fu8, and only about 2.3× as wide as fu2 (measurements taken from uncollapsed left clava); fu2 is about 1.3× as long as wide and the combined length of fu1 + fu2 is about 0.9× the length of the pedicel. This paratype also has the discal setae extending to the parastigma and basal fold, though this is not obvious because the setae below the submarginal vein are lighter in color than more apically and both fore wings beyond the level of the basal cell are glued to the card along with the ventrally mounted specimen. Otherwise, the two paratypes closely resemble each other and typical Calosota metallica females, including having a finely sculptured acropleuron (though largely concealed by glue and overhanging wings), about the apical one-fifth of the metatibiae yellowish, and the scape brownish-violaceous with the violaceous luster varying in strength depending on the angle of light.

Similar to Calosota matritensis female paratype 42521, the male holotype of Calosota modesta (Fig. 26) and another MNCN male with the same data but slightly different label than the holotype lack a broad fore wing speculum. The discal setae are yellowish-white and more difficult to differentiate below the submarginal vein, but they extend virtually to the basal cell with only quite a slender lunate bare region along the basal fold and extreme base of the mediocubital fold (Fig. 25). The metatibiae are more widely yellowish in the holotype of Calosota modesta (Fig. 26) than in the non-type male or two Calosota matritensis paratypes. Askew and Nieves-Aldry (2006: 95) noted that the male holotype of Calosota modesta has “unusually coarse mesoscutal sculpture and rather dark coloration” compared to typical Calosota viridis males.

I saw 62 females from the European countries listed above that I assign to Calosota metallica based on a distinct fore wing speculum and other features typical for the species, though a few have the scape yellowish basally near the radicle and seven females collected from four different localities in Spain in 1973 and 1974 (NMPC) have a mostly yellowish scape that is slightly darkened only apically. The females I identify as Calosota metallica range in length from about 2−3.3 mm and all have quite a distinctly bulbous clava varying from about 1.4−1.9× as long as wide, about 1.6−2.3× as wide as the basal width of fu8, and about 2.8−3.8× as wide as fu2, and usually also have the apical subsegment angled to form quite a distinct oblique ventroapical margin compared to a more uniformly convex dorsal margin (Fig. 80). Furthermore, fu2 is about 1−1.3× as long as wide and the combined length of fu1 + fu2 is about 0.6−0.8× the length of the pedicel. Consequently, Calosota matritensis paratype 52522 falls within the range of what I identify as Calosota metallica from Europe. Another 13 females from Hungary (5♀ HNHM), Portugal (1♀ BMNH), Spain (1♀ BMNH, 1♀ MNCN), Sardinia (4♀ BMNH) and Yugoslavia (USNM) with a finely sculptured (coriaceous-alutaceous) acropleuron similar to Calosota metallica more closely resemble Calosota matritensis paratype 42521 because they have the discal setae extending variably conspicuously to the basal fold and parastigma. Individuals vary in length from about 3.2−5 mm and five of the 13 have at least the basal half of the scape yellowish. The five females with a broadly yellow scape vary in length from about 2.75−5 mm, and four of the five have entirely hyaline fore wings. Seven of the females have an entirely dark scape, including Calosota lixobia Erdös (1946) paralectotype no. 6581 (Fig. 24) and 6583 (var. hyperparasita) (HNHM) that I examined. Females with a dark scape are about 4−5.8 mm in length and have the fore wing disc partially infuscate. The remaining female, the smallest (about 2.5 mm), has the outer surface of the scape dark but the inner surface more obviously brownish-yellow basally and the fore wings hyaline. The different females typically have uniformly yellowish to brown discal setae, though often the setae are at least partly whitish below the submarginal vein or at least the parastigma (Fig. 24). These females are usually much larger and/or have a darker body than those of Calosota metallica (cf. Figs 22, 24), and sometimes have at least the basal half of the scape yellow and/or variably extensively infuscate fore wings (Fig. 24), but other than lacking a distinct fore wing speculum the only feature that appears to differentiate them consistently from Calosota metallica is flagellar structure. The clava ranges from about 1.5−1.9× as long as wide, but is less distinctly clavate than for Calosota metallica females, being about 1.2−1.5× as wide as fu8 and only about 1.5−2.4× as wide as fu2. Furthermore, fu2 ranges from about 1.3−1.8× as long as wide and the combined length of fu1 + fu2 is about 0.9−1.7× as long as the pedicel. Relative to smaller females, larger females tend to have the length of fu2 and the combined length of fu1 + fu2 compared to the pedicel longer. Except for its comparatively slender clava, most relative antennal dimensions for Calosota matritensis paratype 42521 are intermediate between that of typical Calosota metallica and the other larger females discussed above, which likely is correlated with its relatively small body size.

I also saw 23 European males (BMNH, MNCN, UCRC) that I assign to Calosota metallica because they have an obvious fore wing speculum. The visibility of the bare region is partly because it is quite broad but also because the discal setae are yellowish to brown rather than white. Another 16 males from Bulgaria, Sardinia, Yugoslavia (BMNH), Hungary (HNHM), and Spain (BMNH, MNCN) are similar to these males, including having the acropleuron finely sculptured, the scape entirely dark, the propodeal callus setose only anteriorly, and the flagellum robust-filiform (cf. Fig. 63). However, they have the discal setae extending variably densely to the basal fold and most have white (hyaline) and therefore comparatively inconspicuous fore wing setae.

In describing Calosota matritensis, Bolívar y Pieltain (1929) did not mention size of the specimens or the presence of a bare region below the base of the marginal vein and parastigma, but did describe fu1 (anellus) as being about two-thirds as long as fu2, fu2 about 1.25× as long as wide, the ultimate funicular almost quadrangular, and the clava distinctly wider than the funicle and slightly more than 1.5× as long as wide. All of these features are attributable to females of Calosota metallica and I therefore follow the conclusion of Askew and Nieves-Aldry’s (2006), who purportedly examined the holotype, that Calosota matritensis is a junior synonym of Calosota viridis and hence Calosota metallica syn. n. However, I believe that Calosota matritensis female paratype 42521 is the opposite sex of the male holotype of Calosota modesta and remove Calosota modesta from under synonymy with Calosota viridis stat. rev. Additional specimens collected in Spain and throughout Europe are needed to determine whether quite conspicuous differences in size, scape color, and other features of the 13 females discussed above represent intra- or interspecific variation and whether Calosota modesta and Calosota lixobia are conspecific or represent distinct species.

Askew and Nieves-Aldrey (2006) synonymized Calosota lixobia under Calosota obscura Ruschka (1921) after examining the lectotype of the former name. In their key to females they differentiated Calosota obscura and Calosota dusmeti Bolívar y Pieltain (1929) from Calosota viridis partly by females having a “relatively strongly reticulately sculptured” acropleuron. As noted above, the acropleuron appears to be finely sculptured in the two female paratypes of Calosota lixobia that I examined, though the acropleura are largely concealed by the wings and glue on the card-mounted specimens (Fig. 24). A minutien-pinned male paralectotype (no. 6585) of Calosota lixobia I examined has both acropleura completely exposed and they are very shallowly reticulate to reticulate-coriaceous anterior to the microsculptured region and elongate coriaceous-alutaceous without any reticulations posteriorly (Fig. 57). I did not examine the remaining fragmentary type of Calosota dusmeti (MNCN) or that of Calosota obscura (NHMW), but was sent a dorsolateral habitus image of the holotype of Calosota obscura. I saw 15 females from France (CNC), Hungary (CNC, HNHM), Italy (BMNH), Spain (MNCN), and South Korea (CNC) that I identify as Calosota obscura based on absence of a fore wing speculum and presence of a meshlike reticulate acropleuron, including posterior to the microsculptured region (Fig. 56), as well as a completely dark scape and almost completely dark legs excluding the tarsi (knees and apices of at least pro- and metatibiae very narrowly yellow). Another 16 females from Italy (BMNH), Hungary (BMNH, HNHM), Libya (BMNH), Portugal (BMNH), Sardinia (BMNH), and Spain (BMNH, MNCN) are similar except for usually having the scape yellowish or at least quite obviously brownish basally rather than entirely dark, and the knees distinctly yellow if not the metatibia and often the mesotibia extensively light colored. The latter females would key to Calosota dusmeti using Askew and Nieves-Aldrey (2006). Similar to the females discussed above with a finely sculptured acropleuron, those with a more reticulate acropleuron and dark or partly yellowish scape can have entirely hyaline or partly infuscate fore wings. In addition to the females I saw 27 males from Bulgaria, France, Italy, Sardinia (BMNH) and Spain (BMNH, MNCN) that have the acropleuron finely reticulate anteriorly and meshlike reticulate-coriaceous posteriorly, and at least some of the flagellomeres noticeably separated by a short pedicel as keyed by Askew and Nieves-Aldry (2006) for males of Calosota obscura. All the males have a dark scape and I was unable to satisfactorily differentiate more than one species based on males. Askew and Nieves-Aldrey (2006) suggested that Calosota dusmeti might be nothing more than a color form of Calosota obscura. The color variation observed for females, and all males being quite similar may support this hypothesis. If the “light colored” morphotype is a separate species from Calosota obscura, then its senior synonym likely is Calosota violascens Masi (1922) rather than Calosota dusmeti based on the original description of Calosota violascens.

Interestingly, females of both the coriaceous and reticulate acropleuron morphotypes discussed above that resemble Calosota metallica to greater or lesser extent except for the absence of a broad speculum vary quite conspicuously in size and can have an entirely yellow to entirely dark scape and hyaline or partly infuscate fore wings. A comprehensive revision of Palaearctic Calosota is required to determine whether color and/or acropleural sculptural differences reflect intra- or interspecific variation and establish correct nomenclature. Because of acropleural sculpture I suspect that Calosota lixobia is not a synonym of Calosota obscura as proposed by Askew and Nieves Aldrey (2006), but I do not reestablish Calosota lixobia because I did not examine the lectotype of Calosota lixobia or the holotype of Calosota obscura and because the validity of acropleural sculpture as opposed to color differences for differentiating species within the complex requires further study. Certainly, more than one species in a Calosota lixobia, Calosota modesta, Calosota violascens, Calosota dusmeti, Calosota obscura complex is indicated. For example, I saw three females from Greece (CNC) that have a coriaceous-alutaceous acropleuron and a basally yellow scape, but which have the propodeum very densely setose laterally and behind the spiracle to its inner margin so that the cuticle is largely obscured. Females discussed above with either a finely sculptured or reticulate acropleuron, regardless of scape color, have the propodeum setose ventrolaterally only to about the outer margin of the spiracle and more sparsely setose so that the cuticle is clearly visible. Four males from Cyprus (BMNH) likely represent the opposite sex of the females from Greece because they also have the propodeum quite densely setose to the posterior margin of the callus and medially to the level of the inner margin of the spiracle, whereas Calosota lixobia and Calosota obscura males are similar to those of Calosota metallica in having the callus less densely setose and only over about its anterior half to two-thirds. However, one of the females I identify questionably as Calosota lixobia from Spain (BMNH) has the propodeal callus quite densely setose, though only to the outer margin of the spiracle.

Although Askew and Nieves-Aldrey (2006) did not formally synonymize the names, in the list of synonymy for Calosota viridis they included Calosota grylli Erdös (1955) as a questionable synonym. Calosota grylli was described and subsequently keyed by Erdös (1960) as having a fore wing speculum similar to Calosota viridis, but the gaster almost twice as long as the combined length of the head and mesosoma (“3:5” according to original description) as compared to only slightly longer than the head and mesosoma in Calosota viridis. Because they considered relative gastral length to be variable, Askew and Nieves-Aldrey (2006) listed Calosota grylli as a questionable synonym. I examined the lectotype female of Calosota grylli (NMPC, Fig. 23)plus 12 other females that I identify as this species from Bulgaria and Yugoslavia (5 BMNH, 7 NMPC). Females of Calosota grylli are differentiated from those of Calosota metallica by having the metasoma about 1.9–2.1× as long as mesosoma, the syntergum obviously longer than wide (about 1.2–1.7× transcercal width) and, as noted by Erdös (1960), have white setae on the fore wing so that the setation is inconspicuous (cf. Figs 22, 23).Females of Calosota metallica typically have more obvious setation because the setae usually are yellowish to brown, though rarely white. Furthermore, females of Calosota grylli differ quite conspicuously from those of Calosota metallica by having a yellowish tegula and quite a deep and distinct, circular pit within the scrobal depression at the dorsal level of the interantennal region (Fig. 8) (normally concealed by the scapes). Females of Calosota metallica have at most a very shallow, vertical depression on the upper face (Figs 7, 68). I also saw eight males from Bulgaria and Yugoslavia (1 BMNH, 7 NMPC) that I identify as Calosota grylli. These males have dark tegulae and the propodeal callus setose only anteriorly similar to those of Calosota metallica, but similar to Calosota grylli females have a circular depression on the upper face and white discal setae. Furthermore, the flagellum is quite distinctly clavate (Fig. 64), widening toward an obviously wider clava, with both fu1 and fu2 slightly longer than wide and fu8 somewhat transverse, and with the funiculars less conspicuously setose with less strongly curved setae than for males of Calosota metallica (cf. Figs 63, 64),as originally illustrated for the male paralectotype of Calosota grylli by Erdös (1955, fig. 4e).

Within the European material I examined I also saw 11 females from Spain (10 BMNH, 1 NMPC) that have a broad fore wing speculum, lack a pit on the upper face, and have a comparatively short gaster and syntergum similar to Calosota metallica. However, similar to Calosota grylli they have white discal setae and the tegula comparatively bright yellow except brown apically. The females also have at least the extreme base of the scape, but usually about its basal quarter and sometimes up to about its basal half yellow, and differ from both Calosota metallica and Calosota grylli in having the mesoscutum quite densely and conspicuously setose (setae slightly lanceolate) compared to the scutellum, the head comparatively transverse (about twice as wide as long), the ocelli forming a more or less equilateral triangle (POL and LOL subequal), and the length of the flagellum + pedicel slightly shorter or at most equal to the width of the head. Females of Calosota metallica and Calosota grylli have a somewhat more transverse quadrangular head (about 1.75–1.9× as wide as long), a flatter ocellar triangle (POL about 1.4–1.5× LOL) and a slightly longer flagellum + pedicle (about 1–1.1× width of the head). I also saw five males from Spain collected at the same time and place as some of the unidentified females. These males are similar to those of Calosota grylli in having white and therefore comparatively inconspicuous discal setae. However, unlike either Calosota metallica or Calosota grylli males, the propodeal callus is setose to its posterior margin at least laterally and at least about the basal half of the tegula is bright yellow. The flagellum is also only about 0.9× the width of head, fu1 and fu2 are both slightly transverse and of similar length, and fu8 is slightly but obviously transverse (Fig. 65). I consider these males as the opposite sex of this apparently undescribed species. Unlike females, the males do not have a basally yellow scape and though the mesoscutum is not as conspicuously setose as females the slightly lanceolate setae are more conspicuous than for males I identify as Calosota metallica and Calosota grylli.

#### Calosota panamaensis sp. n.

urn:lsid:zoobank.org:act:71E096D6-ABAB-4F99-9EEF-F891043BC308

Figs 21 40 

##### Etymology.

Based on the country of origin of the only known specimen.

##### Type material.

HOLOTYPE ♀ (AEIC). Panama, nr Hato Del Volcan, 4700 feet, Jul. 1982, B. Gill; CNC Photo 2009-37; Holotype Calosota panamaensis Gibson.

##### Description.

###### FEMALE

(Fig. 40). HOLOTYPE: length about 3.5 mm (mesonotum arched). Color. Head with frontovertex, parascrobal region and paraclypeal region dark brown with obscure reddish-violaceous sheen under some angles of light, scrobes and interantennal region to ventral margin of toruli dark or variably extensively bright violaceous-purple under some angles of light, and most of clypeal region and transverse band below toruli bluish-green. Maxillary and labial palpi dark. Antenna with scape brownish-yellow over almost basal quarter, otherwise dark brown with bluish-green lusters under some angles of light, pedicel with similar bluish-green luster, but flagellum dark brown. Tegula yellow. Mesonotum (Fig. 21) bright green with variable reddish-coppery reflections under different angles of light (frenal area concealed by dorsellum). Acropleuron greenish anteriorly similar to mesonotum, but grading to bluish-violaceous posteriorly. Legs (Fig. 40) with front leg extensively light brownish-yellow with knee and apex of tibia more distinctly yellow, but tibia subbasally and dorsally except for apex dark brown with greenish luster under some angles of light; middle leg yellowish beyond coxa except dorsal surface of tibia somewhat darker brownish-yellow at least subbasally; hind leg mostly yellowish beyond coxa, but femur more brownish, particularly ventrally, over about basal half and dorsal surface of tibia slightly brownish subapically. Fore wing hyaline; setae uniformly brown. Gaster (Fig. 40) mostly brown dorsally, but apex of penultimate tergum, syntergum more obscurely, and terga laterally bluish-green to violaceous.

*Structure.* Head in dorsal view about 1.9× as wide as long, with IOD about 0.4× head width; IOD: MPOD: OOL: POL: LOL = 55: 13: 8: 16: 10; in frontal view about 1.25× as wide as high, with about middle of torulus in line with lower orbits; malar space about 0.47× eye height. Head with frontovertex and parascrobal region meshlike coriaceous to about level of interantennal region, the sculpture more obscurely tapered ventrally between smooth and shiny scrobes and interantennal region dorsally, though interantennal region becoming increasingly more conspicuously coriaceous ventrally; clypeal region microcoriaceous and paraclypeal region mostly obliquely coriaceous-alutaceous, though paraclypeal region and lower parascrobal region smooth and shiny along inner orbit. Head with brownish setae except for bare scrobal depression. Antenna (Fig. 40) with flagellum clavate; length of flagellum + pedicel about 1.5× as long as width of head; scape: pedicel: fu1–fu8: clava = 61(12): 23(10): 11(7), 16(8), 17(9), 18(9), 18(9), 15(9), 15(10), 15(13): 52(21). Mesoscutum (Fig. 21) strongly reticulate, the reticulations obviously larger medially than on lateral lobes but even largest reticulations very slightly concave rather than distinctly flat-bottomed, and with inconspicuous brownish setae; notaulus extending from spiracle as curved furrow on inclined anterior surface, without distinct anteroadmedian line and neither line extending posteriorly on dorsal surface; parapsidal line a more distinct microsculptured region. Axilla elongate-slender, separated by about 4× own width. Scutellum (Fig. 21) low convex, about 1.25× as long as wide; meshlike reticulate with concave reticulations similar in size to that on lateral lobes; with inconspicuous brownish setae. Mesopleuron with exposed, setose lower mesepimeron (cf. Fig. 53); acropleuron finely sculptured, obliquely coriaceous-alutaceous anterior to oblique microsculptured region and very finely longitudinally coriaceous-alutaceous to coriaceous-aciculate posteriorly. Fore wing with cc: mv: stv: pmv = 36: 34: 10: 20; basal cell entirely setose; cubital area extensively bare but setose anteriorly along mediocubital fold and closed by setae along posterior margin over about apical half; disc uniformly setose except for slightly developed bare band at juncture of basal and mediocubital folds. Metacoxa setose along dorsal and ventral margins and over about basal half of outer surface. Propodeum with callus setose to posterior margin; bare anteriorly between spiracle and foramen. Gaster (Fig. 40) about 2.3× as long as mesosoma; with brownish setae dorsally but more whitish setae laterally; posterior margin of penultimate tergum not extending to level of cerci, the precercal portion equal to about one-third distance between cerci; syntergum with medial length measured to apex of penultimate tergum about 1.6× transcercal width and about 0.9× as long as penultimate tergum.

###### MALE.

Unknown.

##### Biology.

Unknown.

##### Distribution.

**Map 5.**
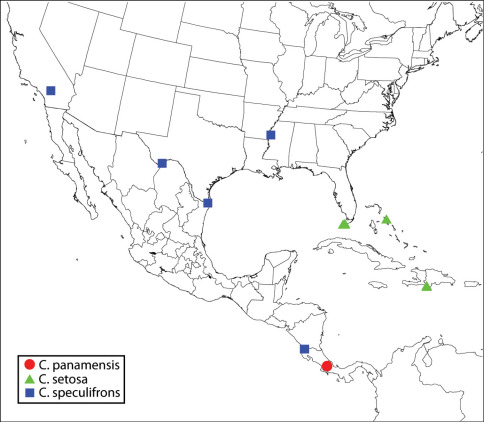
Distribution of Calosota panamaensis, Calosota setosa and Calosota speculifrons.

Known only from Panama (Map 5).

##### Recognition.

Among the four species having a coriaceous frontovertex and extensively smooth and shiny scrobal depression (see under Calosota albipalpus), Calosota panamaensis is most similar to Calosota speculifrons (see further under latter species). It is quite possible that females collected in the future may have more extensively and conspicuously brown legs than the holotype. The short precercal portion of the syntergum of the holotype does not appear to be an artifact of preservation because it is as strongly and uniformly sculptured as the remainder of the syntergum.

#### Calosota setosa sp. n.

urn:lsid:zoobank.org:act:B4AE6AAE-8687-42B9-81D8-1BA6CED60E19

Figs 10 43 47, 51 77 

##### Etymology.

From the Latin word setosus, ‘bristly’, in reference to the comparatively conspicuously setose body of females, including the metacoxae.

##### Type material.

HOLOTYPE ♀ (CNC no. 23927). BAHAMAS, W.I. [West Indies], Eleuthera, Rainbow Bay, 16–26.X.85, J.R. Wiley, MT; CNC Photo 2009-32; Holotype Calosota setosa Gibson. ALLOTYPE♂ (CNC). Same data as holotype except collected 1.X.86; CNC Photo 2009-3; Allotype Calosota setosa Gibson.

Additional paratypes: **BAHAMAS**. Same data as holotype (1♀, 1♂ CNC); same data except 1.VII.87, D.B. & R.W. Wiley (1♀ FSCA). **DOMINICAN REPUBLIC**. Prov. Pedernales, Sierra Bahoruco, 9.5 km. N Cabo Rojo, VII.90, L. Masner, desert (1♂ CNC); 21 km. N Cabo Rojo, 19–20.VI.76, R.E. Woodruff & E.E. Grissell (1♀ FSCA). **USA**. ***Florida***: Monroe Co., Big Pine Key, S1, T67S, R29E, 30.VIII–17.XI.85, S&J Peck, Cactus Hammock, malaise & FIT, forest (1♀ CNC, CNC Photo 2009-1); Middle Torch Key, 1–30.XI.86, S&J Peck, hammock forest edge, malaise, 86-95 (1♀ CNC, CNC SEM 2009-39); No Name Key, 23.II–3.VI.86, S&J Peck, hammock forest, malaise & FIT (1♂ CNC, CNC Photo 2009-2); Sugarloaf Key, Kitching’s, NW 1/4, SE 1/4, S25, R27E, T66S, 14.XI.85–25.II.86, S&J Peck, FIT malaise (1♂ CNC, CNC SEM 2009-40).

##### Description.

###### FEMALE

(Fig. 43). HOLOTYPE: length 2.25 mm. Color. Head (Fig. 10) greenish to greenish-blue under most angles of light except scrobal depression and parascrobal region brown or with coppery luster under some angles of light, and with less distinct coppery-brown band extending dorsally from scrobal depression on either side of anterior ocellus. Maxillary and labial palpi white (Fig. 10). Antenna with about basal fifth of scape white (Fig. 10), otherwise dark brown except scape with bluish-purple lusters under some angle of light. Tegula whitish-yellow. Mesoscutum mostly green similar to head but with reddish-coppery medial region anterior to scutellum, the region fading with ill-defined margins anteriorly but extending to about level of posterior margin of notauli; scutellar-axillar complex mostly green but with very slight and limited indication of reddish-coppery luster under some angles of light. Acropleuron (Fig. 51) brown with variably distinctly greenish to purple lusters under different angles of light. Legs (Fig. 43) with front leg whitish-yellow beyond coxa except posterior surface of femur partly light brown and tibia more distinctly brown subbasally; middle leg whitish-yellow beyond coxa except anterodorsal margin of femur with tiny brown spot and tibia subbasally with tiny, almost complete brown band; hind leg whitish-yellow beyond coxa except anterior surface of femur very slightly darker, brownish-yellow basally. Fore wing hyaline; setae uniformly brown. Gaster variably extensively brown to slightly greenish to blue dorsally under different angles of light, but extreme base of Gt1, syntergum except apically, and terga laterally more distinctly bluish-green.

*Structure/setation.* Head in dorsal view about 1.75× as wide as long, with IOD about 0.4× head width; IOD: MPOD: OOL: POL: LOL = 38: 7: 6: 14: 10; in frontal view about 1.2× as wide as high, with dorsal margin of torulus slightly above level of lower orbits; malar space about 0.5× eye height. Head (Fig. 10) with frontovertex, dorsal most part of scrobal depression and most of parascrobal region finely meshlike coriaceous; scrobal depression mostly and interantennal region dorsally smooth and shiny, interantennal region ventrally finely coriaceous-alutaceous; clypeal region microcoriaceous and paraclypeal region obliquely coriaceous-alutaceous, the sculpture only slightly finer narrowly along inner orbit. Head with whitish seta except for bare scrobal depression. Antenna (Fig. 43) with flagellum clavate; length of flagellum + pedicel about 1.5× head width; scape: pedicel: fu1–fu8: clava = 37(10): 14(7): 5(4), 14(4), 14(5), 13(6), 12(6), 11(6), 10(6), 9(7): 36(10). Mesoscutum (Fig. 77) more or less uniformly reticulate, the flat-bottomed, meshlike reticulations slightly but not conspicuously larger medially than laterally, and with sparse, white setae; notaulus extending from spiracle as shallow furrow on inclined anterior surface; anteroadmedian line not evident; parapsidal line inconspicuous, a short microsculptured region. Axillae elongate-slender, separated by about 5× own width (Fig. 77). Scutellum low convex, about 1.2× as long as wide; elongate reticulate-strigose (Fig. 77); with inconspicuous white setae. Mesopleuron with reduced lower mesepimeron, but narrow vertical surface under convex acropleuron above base of mesocoxa with 2 setae projecting between bases of meso- and metacoxae (cf. Fig. 81); acropleuron without distinct microsculptured region, coriaceous to obliquely coriaceous-alutaceous anteriorly longitudinally elongate coriaceous-alutaceous to coriaceous-aciculate over about posterior half. Fore wing with cc: mv: stv: pmv = 42: 46: 10: 14; basal cell entirely setose; cubital area bare except closed by setae along posterior margin over about apical third; disc setose except for small bare region at juncture of base of basal and mediocubital folds. Metacoxa with outer surface completely setose (Fig. 51). Propodeum with callus setose to posterior margin (Fig. 51); bare anteriorly between spiracle and foramen. Gaster (Fig. 43) about 1.7× as long as mesosoma, with sparse, inconspicuous white setae dorsally and much denser and more conspicuous, slightly lanceolate white setae laterally; penultimate tergum with posterior margin extending to level of cerci; syntergum about 1.5× as long as transcercal width, evenly convex, and about 1.4× length of penultimate tergum.

###### MALE

(Fig. 47). ALLOTYPE: length 2.1 mm. Similar to female except as follows. Color. Head with brownish-coppery region of scrobal depression extending completely to anterior ocellus and mesoscutum and scutellar-axillar complex more extensively though diffusely reddish-coppery, including scutellum and mesoscutal lateral lobes anteriorly; legs almost completely whitish-yellow beyond coxae except for distinct brown region dorsally on protibia and slight brownish tinge posteriorly on profemur and subbasal brownish spot on mesotibia.

*Structure/setation.* Head in dorsal view about 1.9× as wide as long, with IOD about 0.5× head width; IOD: MPOD: OOL: POL: LOL = 41: 11: 5: 15: 9; in frontal view about 1.28× as wide as high, with ventral margin of torulus about at level of lower orbits; malar space about 0.5× eye height. Antenna (Fig. 47) with flagellum elongate-filiform and conspicuously setose, the setae somewhat longer than width of respective flagellomere and curved; length of flagellum + pedicel about 2.2× width of head; scape: pedicel: fu1–fu11 = 31(10): 16(8): 2(5): 24(7): 25(7): 25(7): 23(7): 20:(7): 19(7): 18(7): 16(7): 14(7): 15(6) (including very small apical subsegment). Fore wing cc: mv: stv: pmv = 55: 60: 10: 18. Mesopleuron without projecting setae from slender region below acropleuron above base of mesocoxa. Metacoxa with outer surface more extensively bare mediolongitudinally.

##### Variation.

Females range in length from about 2.1–3.4 mm and males from about 2.1–2.6 mm. One exceptionally large female (FL: Cactus Hammock, Fig. 51) has the head mostly dark brown except for slight coppery or greenish lusters under some angles of light and the mesosoma and gaster more or less uniformly reddish-coppery except for small greenish regions on the gastral terga dorsolaterally. The legs are almost completely yellow beyond the coxae, though with a similar color pattern to the holotype, and the outer surface of the metacoxa is much more densely and conspicuously setose. The mesoscutum is also more strongly reticulate with smaller reticulations so that they are more concave than flat-bottomed. The acropleuron is very shallowly reticulate-coriaceous anteriorly below the tegula and five curved setae project from under its posteroventral margin between the meso- and metacoxae (Fig. 51). The head also has the scrobal depression much more deeply concave; this apparently is because the face is collapsed below the anterior ocellus, but the vertex is not collapsed and the IOD is only one-third the width of the head. The ocelli are also relatively larger, with the IOD: MPOD: OOL: POL: LOL = 43: 13: 5: 14: 10. Additional specimens from Florida are necessary to better access variation, but I currently consider the Cactus Hammock female to be conspecific with the other female from Florida and those from the West Indies. Also, the single female and male from Dominican Republic differ from other type specimens in having a strongly bluish-green to purple mesosoma. The female has about the anterior third of the mesoscutum bright greenish (region narrowly blue posteriorly) and the posterior two-thirds violaceous to purple, the regions abruptly delimited posteriorly, whereas the scutellar-axillar complex is greenish-blue. The male has a more extensively bluish-green mesoscutum, including medially and very narrowly posteriorly, but the lateral lobes are partly violaceous to purple as for the female. The female has only the extreme base of the scape white, whereas the male lacks a distinct basal white region from the scape; the male also has the posterior surface of the profemur darker than for other males and with a slight purple luster. I interpret these differences as intraspecific variation, but additional specimens from Dominican Republic are required to better evaluate the color differences.

##### Biology.

Unknown.

##### Distribution.

Florida Keys and West Indies (Map 5). One of four New World species that may have had a Neotropical origin (see further under distribution for Calosota albipalpus).

##### Recognition.

Individuals of Calosota setosa are most similar to those of Calosota albipalpus, as discussed under the latter species.

#### Calosota speculifrons sp. n.

urn:lsid:zoobank.org:act:4A76C42F-DB8F-4806-90BE-01B8E0F77404

Figs 20 29 39 50, 53 78 

##### Etymology.

From the Latin words speculum, ‘mirror’, and frons, ‘forehead’, in reference to the smooth and shiny scrobal depression that is one of the differentiating features of this species.

##### Type material.

HOLOTYPE ♀ (UCDC). USA: MS [Mississippi], Washington Co., nr Stoneville Delta Exp. Forest, 33°28'N; 90°54'W, 3–17.V.99, N.M. Schiff; CNC Photo 2009-30; Holotype Calosota speculifrons Gibson. ALLOTYPE♂ (CNC). COSTA RICA: Guanacaste Prov., Guanacaste Nat. Park, 23.III–13.IV.1986, D. Janzen & I. Gauld; sector Santa Rosa, 10°51'N; 85°37'W, 250–300m; pk. hdqts., H-2-C, young scrubby woodland, shade; CNC Photo 2009-6; Allotype Calosota speculifrons Gibson.

Additional paratypes. **COSTA RICA**. Same data as allotype except as follows: 2–23.III.86, H-1-O, clearing (1♂ CNC); 13.IV–4.V.86, SE-6-C, Bosque San Emillio, deciduous forest (1♂ CNC); 31.I–21.II.87, H-3-O, clearing in scrubby woodland (1♂ CNC). **USA**. ***California***: San Bernardino Co., Oak Glen, 1500 m. el., 34°2'N; 116°57'W, Malaise trp into ETOH, 27.VI–5.VII.85, Robert E. Wagner (1♀ CNC, CNC Photo 2009-5, CNC SEM 2009-37). ***Texas***: Brewster Co., Big Bend Nat. Pk, Window Trail, 29.V.83, W. Suter, #83-46, moldy leaf litter stream-side (1♀ CNC, CNC Photo 2009-4). Brnsvlle [Brownsville], bred, Insectary No.4, ‘04, H.S. Barber (1♀ USNM).

Excluded from type series. **BRAZIL**. Nova Teutonia, 6.III.41, F. Plaumann (1♀ USNM); Nova Teutonia, 27°11'S; 52°23'W, 300–500 m., VI.72, F. Plaumann (1♂ CNC).

##### Description.

###### FEMALE

**Figures 39−46.**
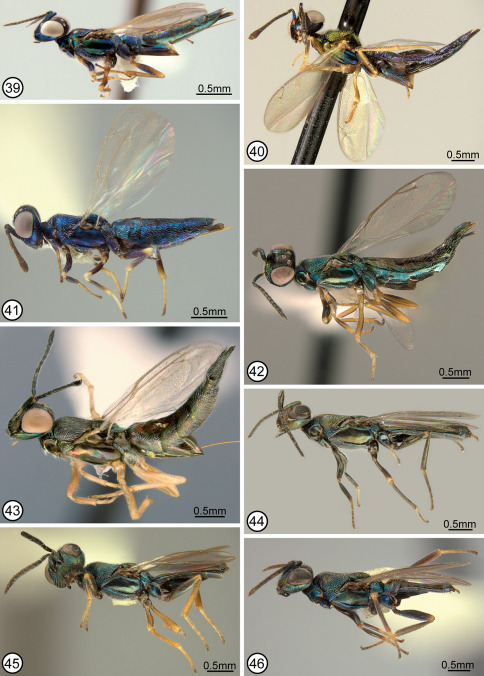
**39−43** lateral ♀ habitus: **39** Calosota speculifrons (5) **40** Calosota panamaensis (holotype) **41** Calosota metallica (28) **42** Calosota acron (13) **43** Calosota setosa (1). **44−46** lateral ♂ habitus: **44** Calosota elongata (allotype) **45** Calosota longiventris (23) **46** Calosota aestivalis (18). Numbers in parentheses = CNC Photo 2009-no.

(Fig. 39). HOLOTYPE: length 4.9 mm. Color. Head (Fig. 20) with frontovertex dark except for ventrally tapered greenish-blue spot below anterior ocellus, scrobal depression, interantennal region, lower parascrobal region and smooth area of paraclypeal region along inner and lower orbits distinctly purple, and with narrow greenish band below level of toruli separating purple region from broad dark band along oral margin having slight coppery luster under some angles of light. Maxillary and labial palpi dark. Antenna dark brown with scape, pedicel, and fu1 under some angles of light with bluish-green to purple lusters. Tegula brown. Mesoscutum (Fig. 20) dorsally with somewhat bell-shaped coppery or slightly greenish-brown region, the region extending across width of scutellar-axillar complex posteriorly but narrowed to subparallel longitudinal margins between obscure notauli anteriorly, with surface lateral to region anteriorly more blue to greenish, and dorsally lateral lobe extensively violaceous-purple though more greenish-blue along lateral margin under some angles of light; scutellar-axillar complex similar in color to dorsomedial region. Acropleuron anteriorly greenish with slight coppery luster under some angles of light, but grading to bluish-purple posteriorly. Legs (Fig. 39) with trochantellus, knee, apex of tibia, and tarsus of front leg yellow, femur otherwise dark brown except posterior surface bluish-purple under some angles of light, and tibia otherwise dark dorsally and ventrally with anterior and posterior surfaces lighter brownish-yellow; middle leg extensively brownish-yellow beyond coxa except tibia somewhat darker subbasally, but knee, tibia apically, and tarsus more distinctly yellow; hind leg extensively yellowish to brownish-yellow beyond metacoxa except anterior surface of femur mesally dark with purplish luster, the lighter region of femur broader dorsoapically. Fore wing hyaline; setae uniformly brown. Gaster mostly coppery or reddish-brown dorsally, but extreme base of Gt1, penultimate tergum over about apical half, and syntergum except posteriorly, greenish-blue.

*Structure/setation.* Head in dorsal view about 2× as wide as long, with IOD about 0.36× head width; IOD: MPOD: OOL: POL: LOL = 63: 16: 9: 19: 16; in frontal view about 1.15× as wide as high, with dorsal margin of torulus slightly above level of lower orbits; malar space about 0.54× eye height. Head (cf. Figs 9, 10) with frontovertex and upper parascrobal region finely meshlike coriaceous, the sculpture medially tapered ventrally between smooth and shiny scrobes; interantennal region and lower parascrobal region narrowly along scrobal depression coriaceous-alutaceous, but lower parascrobal region broadly smooth and shiny along inner orbit; clypeal region microcoriaceous and paraclypeal region more obliquely granular to reticulate. Head with dark setae except for bare scrobal depression. Antenna (Fig. 20) with flagellum clavate; length of flagellum + pedicel about 1.35× head width; scape: pedicel: fu1–fu8: clava = 85(16): 30(12): 15(8), 20(9), 20(10), 21(10), 19(10), 18(10), 17(10), 16(11): 68(17). Mesoscutum (Figs 20, 78) with inclined, convex part of lateral lobes coriaceous to slightly coriaceous-alutaceous, the cells similar in size to those of frontovertex, but dorsomedially with large, shallow, flat-bottomed, meshlike reticulations, and with dark setae dorsally and more whitish setae over inclined surface of lateral lobe; notaulus and anteroadmedian line apparently obscure (covered by head), but at least parapsidal line a distinct microsculptured region. Axillae elongate-slender, separated by about 4× own width (Fig. 78). Scutellum low convex, about 1.2× as long as wide; elongate strigose-reticulate (Fig. 78); with inconspicuous dark setae. Mesopleuron (Fig. 53) with exposed, setose lower mesepimeron; acropleuron obliquely coriaceous-alutaceous anterior to oblique microsculptured region and longitudinally coriaceous-aciculate over about posterior half. Fore wing with cc: mv: stv: pmv = 44: 35: 10: 20; basal cell entirely setose; cubital area bare except closed by setae along posterior margin for about one-third length; disc setose except for slender, arcuate bare band along basal fold and short region of mediocubital fold, but bare region separated from cubital area by line of setae. Metacoxa (Fig. 53) with outer surface setose except within more distinctly concave apical portion. Propodeum with callus setose to posterior margin (Fig. 53); bare anteriorly between spiracle and foramen. Gaster about 1.7× as long as mesosoma, with sparse, inconspicuous whitish setae dorsally and much denser and more conspicuous, slightly lanceolate white setae laterally; penultimate tergum with posterior margin extending to level of cerci; syntergum about 1.6× as long as transcercal width, evenly convex, and very slightly shorter than penultimate tergum.

###### MALE

(Figs 29, 50). ALLOTYPE: length 2.75 mm. Similar to holotype except as follows. Color. Head (Fig. 29) without metallic spot below anterior ocellus and face brownish with reddish-coppery luster; mesoscutum (Fig. 29) with coppery-brown region occupying about medial third of mesoscutum and with more or less subparallel margins over most of length, and lateral lobes more conspicuously bright bluish-green anteriorly and purple posteriorly; middle leg (Figs 29, 50) with femur and subbasal band on tibia darker brown; hind leg (Fig. 50) with femur somewhat more extensively dark with metallic luster and dorsal surface of tibia dark except narrowly basally and apically.

*Structure/setation.* Head in dorsal view about 1.9× as wide as long, with IOD: MPOD: OOL: POL: LOL = 46: 12: 5: 14: 8; in frontal view about 1.3× as wide as high, with ventral margin of torulus about at level of lower orbits; malar space about 0.43× eye height. Antenna with flagellum clavate (Figs 29, 50) similar to female; length of flagellum + pedicel about 1.2× head width; scape: pedicel: fu1–fu8: clava = 49(10): 18(8): 9(5), 11(6), 13(7), 14(7), 13(6), 12(9), 12(10): 35(10). Fore wing with cc: mv: stv: pmv = 43: 36: 10: 20. Metacoxa with outer surface more extensively bare mediolongitudinally.

##### Variation.

Females range in length from about 2.5–4.9 mm and males from about 1.25–2.75 mm. Females other than the holotype lack the metallic spot below the anterior ocellus, the purple luster of the face is sometimes less conspicuous though the interantennal region is always purple under some angles of light, and the mesofemur usually is quite dark brown except yellowish basally and apically. Both sexes sometimes have the mesoscutum more extensively coppery-brown (purplish-coppery in one female) with only the inclined surface laterally being partly bluish-purple to entirely greenish-purple. Males sometimes have the palpi light rather than dark brown. The scutellum of larger individuals is more distinctly strigose-reticulate (Fig. 78) than for smaller specimens, which have a more elongate-reticulate sculpture more similar to the mesoscutal sculpture medially, though always with much more elongate and smaller reticulations. Males typically have the metacoxa almost completely bare mediolongitudinally. All females have fu1 and fu8 at least slightly longer than wide, whereas the apical funiculars can be quadrate to slightly transverse in males, and both sexes can have the postmarginal vein shorter relative to the stigmal vein than described for the holotype and allotype.

I exclude from the type series one female and one male from Brazil that are very similar to other specimens I include in Calosota speculifrons except that the posterior, metallic colored part of the lateral lobes is reticulate in the female and the male has the lateral lobes entirely reticulate. The tips of the antennae are missing from the female, but the male has longer flagellomeres than those from Costa Rica such that the length of the flagellum + pedicel is almost 1.65× as long as the width of the head. Additional specimens from Brazil are necessary to better evaluate species limits. Another female from Nova Teutonia not listed above that bears the same data as the male, except it was collected 2.XII.40 (CNC), more certainly represents an undescribed species. It is about 6.2 mm in length and has about the basal half of the scape, tegula and legs yellow; the mesonotal sculpture and color pattern is similar to Calosota speculifrons but the lateral lobes and part of the mesoscutum dorsally is bright green rather than bluish or purple, as is also the face below about the level of the dorsal limit of the interantennal region. Furthermore, the face above the toruli is entirely, finely coriaceous except for separate smooth and shiny scrobes.

##### Biology.

Unknown.

##### Distribution.

Southern USA at least to Costa Rica (Map 5) and possibly Brazil. One of four New World species that may have had a Neotropical origin (see further under distribution for Calosota albipalpus).

##### Recognition.

Among the four regional species having a coriaceous frontovertex and extensively smooth and shiny scrobal depression (see under Calosota albipalpus), Calosota speculifrons is most similar to Calosota panamaensis because they share dark palpi and an exposed, setose lower mesepimeron (Fig. 53). However, the two species are readily differentiated by mesonotal color and, most conspicuously, a different sculpture pattern of the scutellum relative to the mesoscutum. Individuals of Calosota speculifrons have the most darkly colored legs of the four species with a similar head sculpture, the mesofemur at least being extensively brownish-yellow if not dark brown (Figs 20, 29, 39, 50).

**Figures 47−55.**
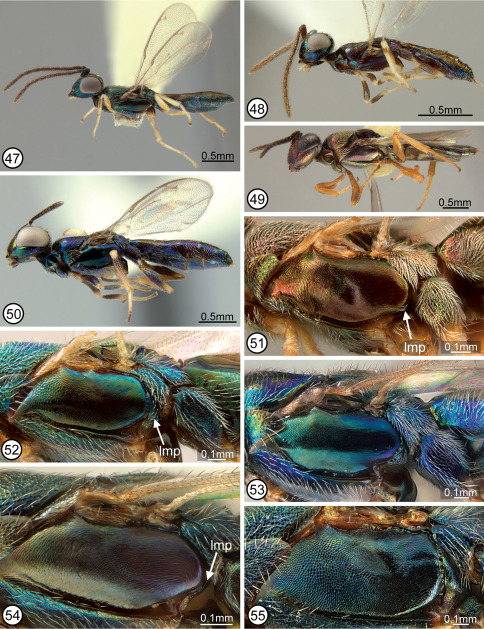
**47−50** lateral ♂ habitus (allotype): **47** Calosota setosa **48** Calosota albipalpus **49** Calosota bicolorata **50** Calosota speculifrons **51−55** lateral ♀ mesosoma/acropleuron **51** Calosota setosa (1) **52** Calosota acron (50) **53** Calosota speculifrons (holotype) **54** Calosota vernalis (43) **55** Calosota aestivalis (44). Numbers in parentheses = CNC Photo 2009-no. Abbreviation: lmp = lower mesepimeron.

**Figures 56−65.**
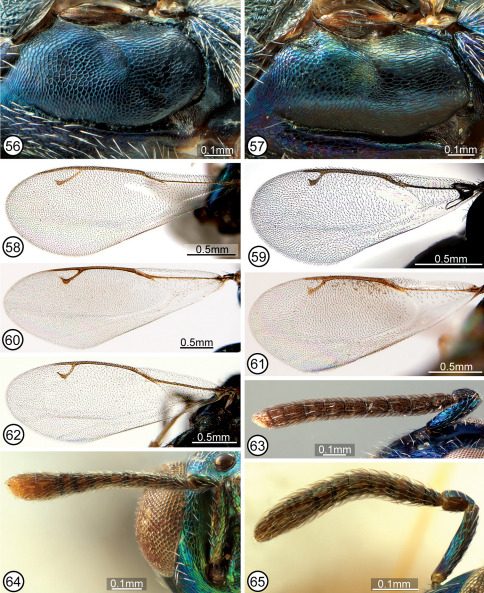
**56−57** lateral mesosoma/acropleuron: **56** Calosota obscura ♀ (45: France, CNC) **57** Calosota lixobia (♂ paratype). **58−62** fore wing: **58** Calosota acron ♀ (48) **59** Calosota metallica ♀ (27) **60** Calosota aestivalis ♀ (47) **61** Calosota bicolorata ♂ (46) **62** Calosota longivena (42). **63−65** ♂ antenna: **63** Calosota metallica (34) **64** Calosota grylli (35: Yugoslavia, NMPC) **65** Calosota sp. (36: Spain, BMNH). Numbers in parentheses = CNC Photo 2009-no. Abbreviation: lmp = lower mesepimeron.

My association of males and females of Calosota speculifrons is based largely on individuals of both sexes having dark palpi and an exposed, setose lower mesepimeron, but this association requires confirmation through rearing or collecting both sexes in either North America or Costa Rica. Unlike other North American species with a similar head sculpture, males I assign to Calosota speculifrons have a clavate (Figs 29, 50) rather than an elongate-filiform, setose flagellum (Calosota albipalpus, Fig. 48 and Calosota setosa, Fig. 47). Furthermore, I saw two males from Dominican Republic (CNC) that I do not assign to any species or describe as new that are similar to males I identify as Calosota speculifrons except they have white palpi and lack an exposed lower mesepimeron, similar to Calosota albipalpus and Calosota setosa.

Similar to Calosota speculifrons and Calosota panamaensis, the nominal European species Calosota agrili Nikol’skaya, Calosota bolivari Askew, and Calosota nitens Askew have a finely coriaceous frontovertex and dark palpi, but among other features differ in having an exposed lower mesepimeron that is bare (cf. Fig. 55, see further under Calosota vernalis).

#### Calosota vernalis

Curtis

Figs 3 27 32 54 69, 73

Calosota vernalis Curtis 1836: folio 596. Type data: England: Southgate. Lectotype ♀ (MVMA; not examined), designated by Graham 1969: 90.
Calosoter aestivalis Walker 1837: 359–360. Type data: England: near London. Syntypes, ♀ and ♂ (BMNH, type no. 5.1620; not examined). Synonymy by Graham 1969: 90. Homonym of Calosota aestivalisCurtis (1836).

##### Diagnosis.

(Fig. 32) and MALE. Head (Figs 3, 69) with frontovertex finely, meshlike coriaceous or at most only very shallowly reticulate in small part, but scrobal depression quite distinctly reticulate to transversely reticulate-alutaceous excluding smooth and shiny scrobes; ocelli in acute triangle with POL subequal to or only slightly greater than OOL; mesonotum (Figs 27, 73) meshlike reticulate and uniformly dark to bluish-green or depressed medial part of mesoscutum and scutellum dark or sometimes with slight coppery luster under some angles of light; axilla elongate-slender, sculptured surface usually mostly obliquely angled rather than a flat dorsal surface and separated by at least about 6× own width (Figs 27, 73); fore wing hyaline, without linea calva or speculum; acropleuron finely, obliquely coriaceous-alutaceous anteriorly and longitudinally coriaceous-alutaceous to very finely alutaceous-aciculate posteriorly (Fig. 54); lower mesepimeron reduced (Fig. 54).

Male with at least fl2−fl6 longer than wide and quite densely setose with short, only slightly curved setae projecting at about a 45° or lesser angle relative to the flagellomere.

##### Biology.

A gregarious ectoparasitoid of Trichodes Herbst (Coleoptera: Cleridae) in Megachilidae and Sphecidae nests (Thompson 1955 (as Calosota aestivalis); Askew and Nieves-Aldrey 2006). The host species in Buprestidae and Curculionidae, and possibly Scolytidae (Coleoptera), listed by Noyes (2003) almost certainly refer to Calosota aestivalis. All the original publications cited by Herting (1973) for the various host species were published prior to Graham (1969), who corrected the previously reversed concepts of Calosota aestivalis and Calosota vernalis. Furthermore, even the more recent publications listing Blastophagus minor (Hartig) (Scolytidae) as a host cited Hedqvist (1963), who interpreted Calosota vernalis in the sense of Calosota aestivalis. However, a single male from France (BMNH) is labeled “ex Pinus sylvestris with scolytids”.

##### Regional material examined.

None, but one female (CNC Photo 2009-43, CNC SEM 2009-47) and male labeled: Canada: BC [British Columbia], Surrey, Westcoast Granite, CFS [Canadian Forest Service] 1988-0035-07, quarantine interception, ex log bolt #24 from Norway, em. 20.VII.1988.

##### Distribution.

Noyes (2003) listed Calosota vernalis from several countries in the Palaearctic region; I saw specimens from England (BMNH), France (BMNH), Italy (CNC, CNC SEM 2009-46), Spain (CNC, MNCN), Sweden (CNC), and Yugoslavia (BMNH).

##### Recognition.

**Figures 66−73.**
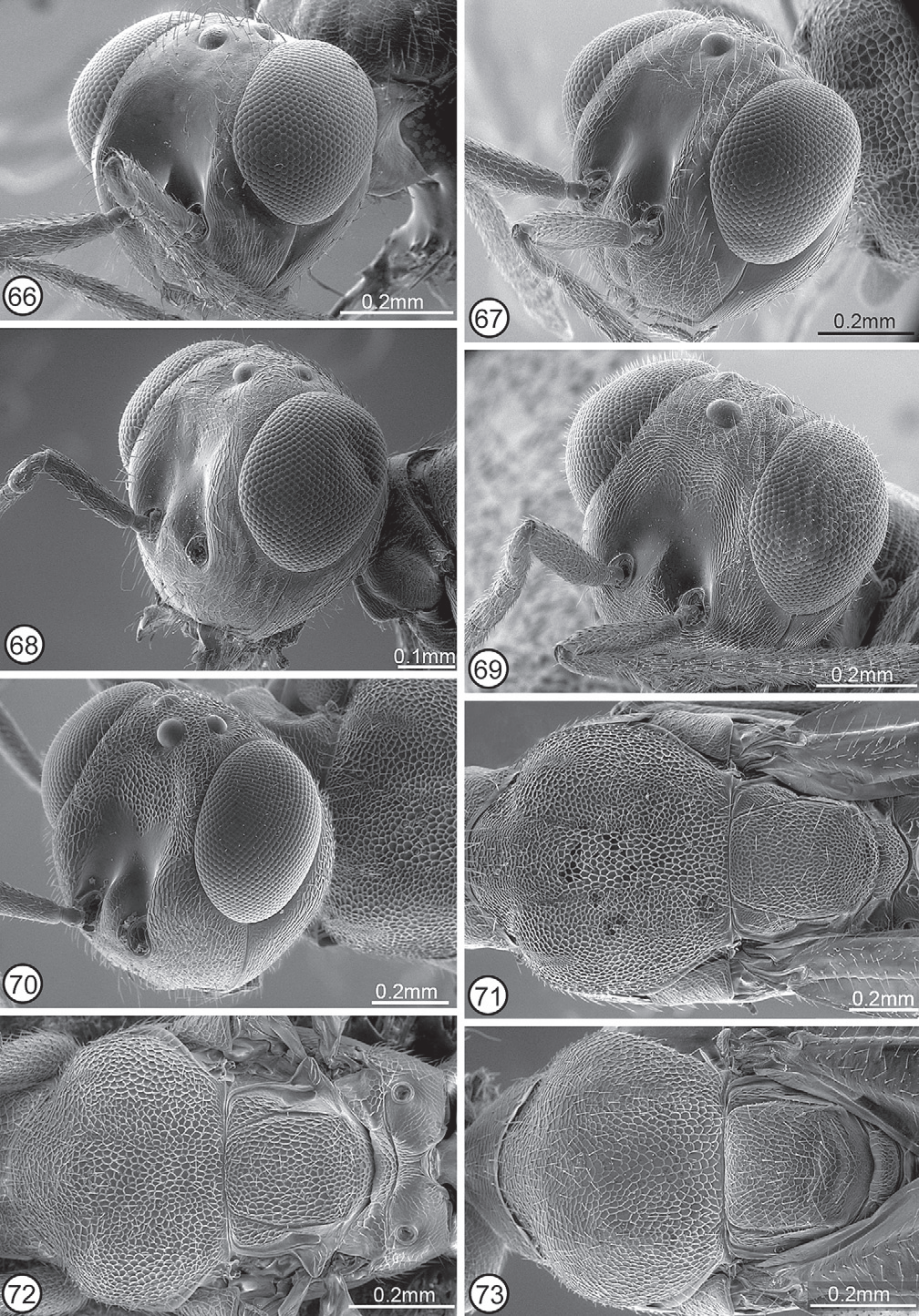
**66−70** head, frontolateral view: **66** Calosota albipalpus ♀ (35) **67** Calosota acron ♀ (34) **68** Calosota metallica (30) **69** Calosota vernalis ♂ (46) **70** Calosota elongata ♀ (50). **71−73** dorsal mesosoma: **71** Calosota elongata ♀ (49) **72** Calosota aestivalis ♂ (48) **73** Calosota vernalis ♀ (47). Numbers in parentheses = CNC SEM 2009-no.

**Figures 74−81.**
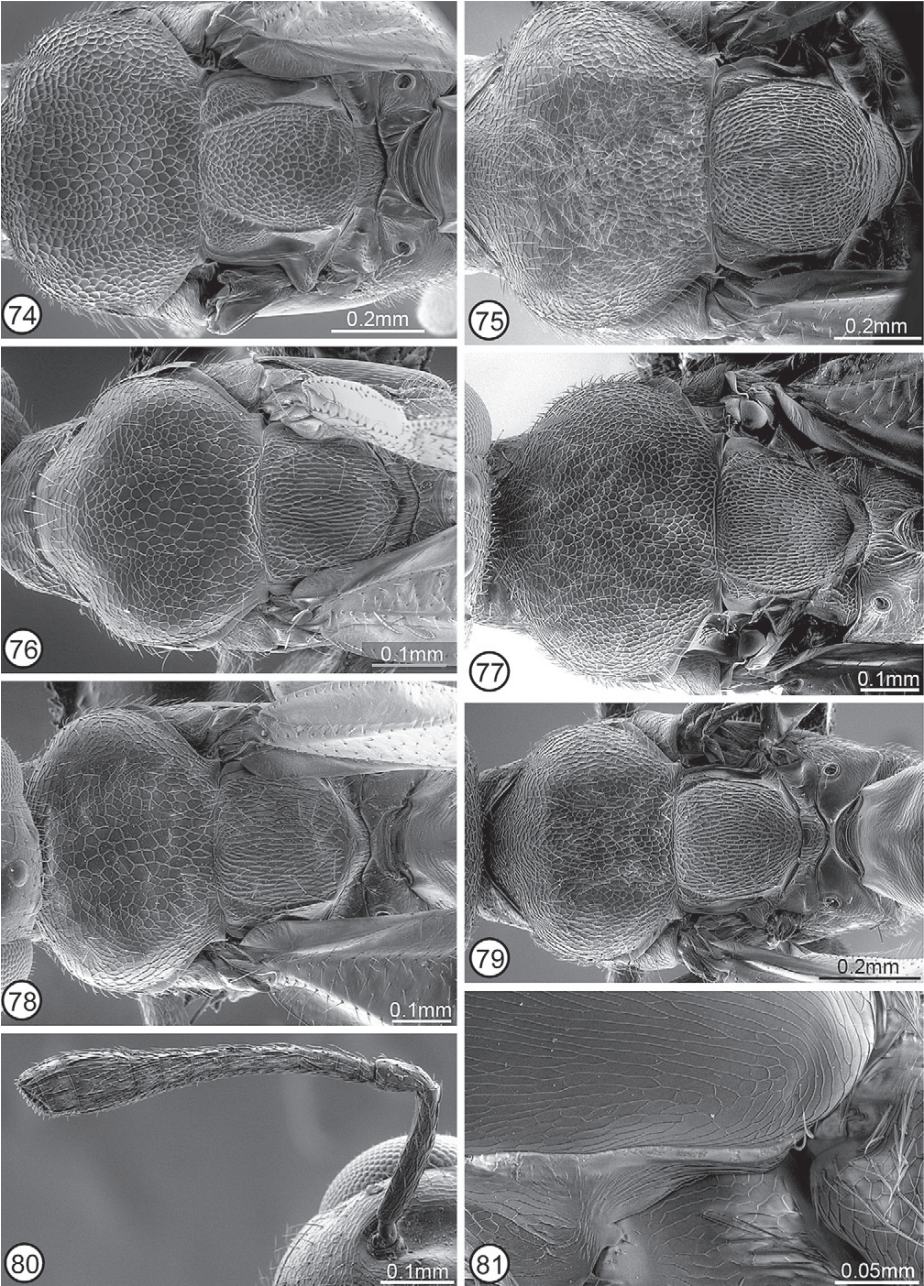
**74−79** dorsal mesosoma ♀: **74** Calosota acron ♀ (34) **75** Calosota bicolorata ♀ (holotype) **76** Calosota albipalpus ♀ (42) **77** Calosota setosa ♂ (40) **78** Calosota speculifrons ♀ (37) **79** Calosota metallica ♀ (32) **80** Calosota metallica, ♀ antenna (30) **81** Calosota albipalpus ♀, posteroventral portion of acropleuron (36). Numbers in parentheses = CNC SEM 2009-no.

Additional features than those used in the key to differentiate Calosota vernalis relative to Calosota aestivalis are provided by Graham (1969), including comparatively narrower axillae and the acropleuron anteriorly being obliquely coriaceous-alutaceous (Fig. 54) rather than at least shallowly meshlike reticulate (Fig. 55). If eventually collected in North America, individuals of Calosota vernalis are most likely to be misidentified as one of the four regional species having a conspicuously sculptured scrobal depression, but unlike any of these species lack an exposed lower mesepimeron (Fig. 54). It also uniquely has an unusual sculpture combination of a finely meshlike coriaceous frontovertex and more coarsely reticulate or at least transversely sculptured scrobal depression (Figs 3, 69). This not only helps differentiate it from Calosota aestivalis, which has both the frontovertex and scrobal depression reticulate (Fig. 2), but also from the nominal European species Calosota agrili Nikol’skaya (1952) (paratype examined, BMNH), Calosota bolivari Askew (2006) (holotype examined, MNCN) and Calosota nitens Askew (2006) (holotype and paratype examined, MNCN), which have the frontovertex coriaceous and the scrobal depression mostly smooth and shiny similar to the New World albipalpus species-group (cf. Figs 9, 10, 66).

Askew and Nieves-Aldrey (2006) noted that the unknown male of Calosota nitens may have the antennal features that they used to differentiate males of Calosota vernalis. The male of Calosota nitens is undescribed and I have not seen any males resembling females of the species. However, based on the two examined type females from Spain and one additional female from Sardinia (BMNH), in addition to having the outer surface of the metacoxa entirely setose, females of Calosota nitens uniquelyhave the propodeal callus setose along the propodeal foramen. Other Calosota typically have the callus setose lateral to the spiracle and sometimes there are 1 or 2 setae behind the spiracle but never along the propodeal foramen. The male of Calosota nitens may also have the propodeum at least sparsely setose along the foramen and if so should be recognized easily.

Although the male of Calosota bolivari is also undescribed, I saw 19 males and 3 females from Spain (CNC, MNCN), 13 males and 3 females from Cyprus (CNC), and 1 male from Corsica (ZSMC) that I identify as this species. The combination of head sculpture (upper part of scrobal depression at most finely coriaceous rather than obviously reticulate) and exposed, lunate lower mesepimeron differentiate the males from those of Calosota aestivalis and Calosota vernalis, respectively. Flagellar structure of Calosota bolivari males is somewhat similar to that of Calosota vernalis, but the funiculars are typically shorter and the setae are conspicuously longer and more distinctly curved, the setae projecting at an obtuse angle and curved so as to be subparallel with the funicular distally. Using Askew and Nieves-Aldrey (2006), the putative males of Calosota bolivari key to Calosota obscura and Calosota aestivalis, but the flagellum is comparatively more gracile with abutting funiculars unlike that described for Calosota obscura, and the acropleuron is finely coriaceous. Of the six females examined, one (El Ventorrillo, 14.VII.91, A. Garrido) was identified as Calosota dusmeti in Askew and Nieves-Aldrey (2006). This apparent misidentification likely was in part because it has about the basal two-thirds of the scape yellowish-orange, unlike the holotype of Calosota bolivari which has an entirely dark scape, but similar to specimens of Calosota dusmeti. However, scape color of other females from Spain and Cyprus, which vary in length from about 2.4−5 mm, varies from entirely or virtually entirely dark to variably extensively yellowish. These females are also very similar to the paratype of Calosota agrili examined, which has about the basal half of the right scape yellowish and the left scape quite dark with only some indication of yellow in its basal half. Another female from Voronezhskaya Oblast, Russia (CNC), identified as Calosota agrili by A. Sharkov has both scapes dark. All the females have the marginal vein about 2.8× as long as the stigmal vein and the carina along the propodeal foramen not quite extending to the anterior margin of the propodeum so that there is a very short medial plical region similar to the holotype of Calosota bolivari. The holotype was described as having the medial length of the propodeum (including plical region and smooth and shiny lunate region posterior to carina extending anteriorly from along foramen) as long as the dorsellum, but this is partly an artifact because the mesonotum is arched and the anterior margin of the propodeum extends over the apex of the dorsellum to reduce its apparent length. Based on the above, I suspect that Calosota bolivari is a junior synonym of Calosota agrili, but hesitate to formalize the synonymy prior to a more comprehensive revision of Palaearctic Calosota.

## Supplementary Material

XML Treatment for Calosota
